# Slick K^+^ channels contribute to cardiac remodeling, fibrosis, and dysfunction in postinfarction hearts

**DOI:** 10.1172/jci.insight.195805

**Published:** 2026-03-17

**Authors:** Jiaqi Yang, Lin Zhu, David Spähn, Melanie Cruz Santos, Sophia Schanz, Selina Maier, Lena Birkenfeld, Helmut Bischof, Anna Roslan, Nina Wettschureck, Oliver Borst, Lucas Matt, Robert Lukowski

**Affiliations:** 1 Department of Pharmacology, Toxicology and Clinical Pharmacy, Institute of Pharmacy, University of Tübingen, Tübingen, Germany.; 2 Max-Planck-Institute for Heart and Lung Research, Department of Pharmacology, Bad Nauheim, Germany.; 3 DFG Heisenberg Group Thrombocardiology, Tübingen, Germany, and Department of Cardiology, Angiology and Cardiovascular Medicine, University of Tübingen, Tübingen, Germany.

**Keywords:** Cardiology, Cell biology, Fibrosis, Heart failure, Potassium channels

## Abstract

Resident cardiac fibroblast–derived (RCF-derived) cardiac myofibroblasts (CMFs) contribute to myocardial repair but also drive adverse ventricular remodeling and contractile dysfunction after myocardial infarction (MI). The sodium-activated potassium channel Slick (Slo2.1) has been described in cardiomyocyte (CM) mitochondria; however, transcriptomic analyses indicate higher Slick expression in RCFs/CMFs. Here, we investigated the role of Slick in cardiac fibroblast function and post-MI remodeling. Using live-cell imaging and whole-cell patch-clamp recordings, we found that plasma membrane Slick channels in RCFs and CMFs regulated potassium (K^+^) efflux and modulated store-operated calcium entry (SOCE), particularly in CMFs. Global Slick KO and conditional CMF-specific KO hearts exhibited reduced fibrosis and preserved left ventricular function after ischemia/reperfusion injury. This cardioprotection was associated with diminished CMF activation and proliferation, reduced inflammation, and improved CM survival after MI. Collectively, these findings identify fibroblast Slick channels as regulators of SOCE-dependent fibrogenesis and demonstrate that their deletion mitigates maladaptive remodeling and functional decline after MI.

## Abstract 2

Slick, a sodium-activated potassium channel present in different cardiac cell compartments, promotes the hyperfunction of scar-forming fibroblasts and consequently heart muscle stiffness and dysfunction after myocardial infarction.

## Introduction

Acute myocardial infarction (MI) remains a leading cause of cardiovascular mortality worldwide (1, 2). Although advances in reperfusion strategies and pharmacotherapy have substantially reduced short-term mortality, long-term heart failure driven by adverse cardiac remodeling and fibrosis continues to pose a major clinical challenge (3). Approximately 20%–30% of MI survivors develop heart failure within 1 year, with a median survival of only 4 years after diagnosis (4, 5).

Resident cardiac fibroblasts (RCFs), contributing to ventricular integrity and electrophysiological conductance among myocardial layers in naive uninjured myocardium, are the primary responding cell population after MI (6, 7). Within the first 3 hours to 3 days after MI, RCFs react to damage-associated molecular patterns released from dead cardiomyocytes (CMs) by producing proinflammatory cytokines and chemokines, thereby promoting debris clearance and driving their proliferation and differentiation into cardiac myofibroblasts (CMFs) (8, 9). CMF activation peaks between days 3 and 14 after MI and is characterized by robust ECM production and upregulation of profibrotic markers, such as α-smooth muscle actin (α-SMA), collagens, and TGF-β (10, 11). Although CMFs arise from multiple cellular origins after MI, RCF-derived CMFs constitute the predominant subpopulation (12). Under injury conditions, these cells uniformly express periostin and represent the principal profibrotic population within the infarcted myocardium (13). As ECM provides structural support to the injured myocardium, balanced scar formation is critical: insufficient repair predisposes to aneurysm and rupture, whereas excessive fibrosis increases ventricular stiffness (14, 15). CMFs, however, persist beyond scar maturation (16–18), sustaining collagen deposition and maladaptive remodeling. Persistent CMF activity promotes CM hypertrophy and loss, ultimately contributing to heart failure with reduced ejection fraction (17). Despite its clinical relevance, current heart failure therapies do not directly target pathological fibrosis (19).

Potassium (K^+^) channels are essential for regulating membrane potential and cellular excitability (20). Emerging evidence implicates multiple K^+^ channel families in cardiac fibroblast activation and fibrotic remodeling (21–26). ATP-sensitive K^+^ (K_ATP_) channels composed of the regulatory sulfonylurea receptor 2 (SUR2) and the inwardly rectifying pore-forming subunit (K_ir_) 6.1 have been identified in RCFs and CMFs, where their upregulation correlates with RCF activation and enhanced ECM production (22). In addition, other types of K^+^ channels have been linked to fibroblast proliferation, collagen synthesis, and myocardial fibrosis across various experimental models (23–26). These findings suggest that K^+^ channel signaling is a critical regulator of fibroblast behavior under pathological conditions.

Sodium-activated potassium (K_Na_) channels comprise Slick (Slo2.1, K_Na_1.2) and Slack (Slo2.2, K_Na_1.1) (27). These channels are activated by intracellular sodium ([Na^+^]_i_) and chloride ([Cl^–^]_i_) levels. Slick is uniquely sensitive to intracellular ATP ([ATP]_i_) depletion, suggesting a role during ischemic conditions characterized by [Na^+^]_i_ influx and rapid [ATP]_i_ depletion (28). Slick has been shown to mediate the cardioprotective effects of anesthetic preconditioning in murine hearts exposed to hypoxia/reperfusion (29). Subsequent studies identified Slick-dependent conductance in murine CM mitochondria, where K^+^ influx is proposed to stabilize mitochondrial membrane potential and maintain ionic homeostasis under ischemic stress (30). Single-cell transcriptomic analyses of murine hearts have, however, revealed that Slick transcripts are more abundant in RCFs and CMFs than in CMs, particularly after MI (31, 32). These observations raise the possibility that Slick may regulate fibroblast function in addition to its established role in CM survival.

We therefore investigated whether and how Slick regulates myo-/fibroblast function and post-MI cardiac remodeling. Primary RCFs, cultured RCF-derived CMFs, and CMF^I/R^ isolated 1 week after ischemia/reperfusion (I_45min_/R_1week_) were analyzed ex vivo. In vivo fibrotic remodeling and cardiac function were assessed 4 weeks after I/R (I_45min_/R_4weeks_) using previously established global Slick KO and newly developed CMF-specific KO (*Postn*i-Cre, mKO) mice. Conditional Slick mutants were generated utilizing the well-established *Postn*i-Cre system because *Postn*^+^ CMFs account for nearly the entire population of RCF-derived CMFs (13, 15, 33).

## Results

### Expression and functional characterization of Slick in RCFs and CMFs in vitro.

**Figure 1:**
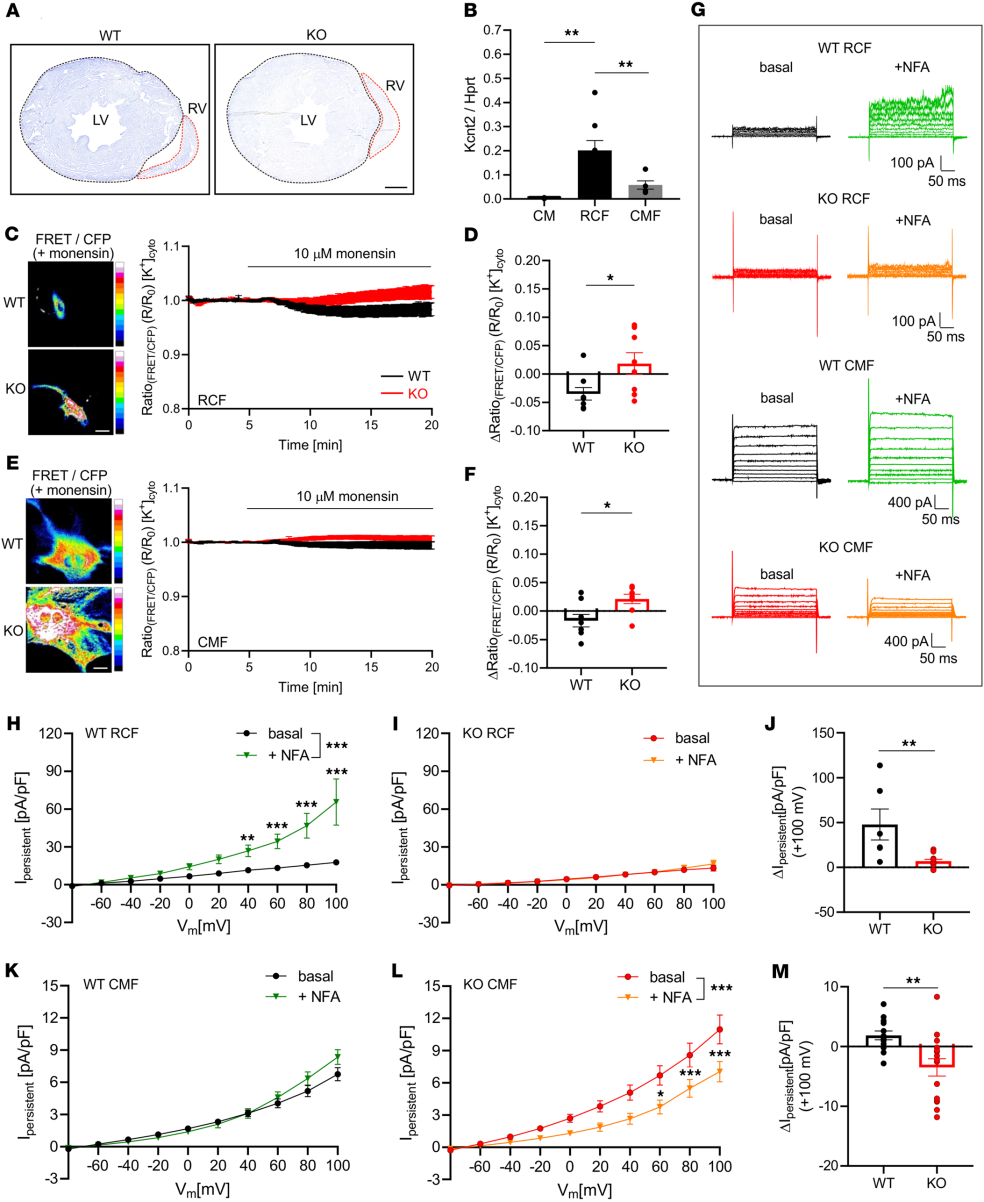
Expression and functional characterization of Slick in RCFs and CMFs. (**A**) Representative alkaline phosphatase staining for Slick protein (blue) in cryosections from WT and KO hearts. Scale bar: 500 μm. (**B**) RT-qPCR analysis of *Kcnt2* transcripts in isolated primary CMs, RCFs, and cultured CMFs, normalized to *Hprt*. One-way ANOVA with Tukey’s multiple-comparison test, **P < 0.01 (*n* = 11 hearts for CMs, *n* = 12 hearts for RCFs, and *n* = 5 hearts for CMFs). (**C**) Representative FRET-based live-cell [K^+^]_cyto_ images in RCFs after 15 minutes of monensin treatment. Higher FRET/CFP ratios appeared as red-white signals. Scale bar: 50 μm. Right panel: normalized average FRET/CFP ratio over time. (**D**) Δ FRET/CFP ratios in WT and KO RCFs. Δ was calculated as R_20min_ – R_5min_, with R_0_ defined as the mean ratio during the first 5 minutes. Mann-Whitney *U* test, *P < 0.05 (*n* = 8 hearts per genotype). (**E**) Representative FRET images and average ratio over time in CMFs during monensin treatment. Scale bar: 50 μm. (**F**) Δ FRET/CFP ratios in WT and Slick KO CMFs. Two-tailed unpaired Student’s *t* test, *P < 0.05 (*n* = 8 hearts per genotype). (**G**) Representative whole-cell currents at baseline and after 5 minutes of NFA. (**H** and **I**) Current-voltage (I-V) relationships of persistent currents in WT and KO RCFs before and after NFA treatment. Two-way ANOVA with Šidák’s multiple-comparison test, ***P < 0.001. (**J**) Δ current density at 100 mV. Nested *t* test, **P < 0.01 (WT: 6 cells from 5 hearts; KO: 11 cells from 4 hearts). (**K** and **L**) I-V relationships of persistent currents in CMFs. Two-way ANOVA with Šidák’s multiple-comparison test, ***P < 0.001. (**M**) Δ current density at 100 mV. Nested *t* test, **P < 0.01 (WT: 13 cells; KO: 15 cells from 4 hearts).

To examine the expression of Slick at the transcript and protein levels, we first performed alkaline phosphatase–based IHC on healthy cardiac tissue sections derived from WT and global Slick KO mice (Figure 1A). After tissue treatment with a specific anti-Slick antibody, WT heart sections developed a strong blue coloration, indicative of Slick expression, while KO heart sections remained unstained.

To further delineate the cell-specific expression profile of Slick channels in the heart, we assessed *Kcnt2* transcript levels, encoding murine Slick, in freshly isolated RCFs and in RCF-derived CMFs after 10 days in vitro. The identity and purity of the isolated populations, as well as their successful transition from RCF to CMF, were confirmed by increased expression of CMF markers *Acta2* (encoding α-SMA) and *Postn* (encoding periostin), in comparison to cell-specific markers identified in endothelial and immune cells or CMs (9, 34) (Supplemental Figure 1A; supplemental material available online with this article; https://doi.org/10.1172/jci.insight.195805DS1). Consistent with single-cell RNA-Seq data from the Tabula Muris consortium (31), RT-qPCR analysis revealed that *Kcnt2* was highly expressed in both RCFs and cultured CMFs, whereas its expression was markedly lower in primary adult CMs (Figure 1B). These data suggest that Slick channels are primarily expressed in fibroblast populations, both at rest and after activation in vitro.

To investigate potential compensatory mechanisms in the absence of Slick, we further analyzed the expression of several other K^+^ channel genes previously reported in healthy and diseased cardiac myo-/fibroblasts (35–37). However, no significant compensatory up- or downregulation was observed in Slick-deficient CMFs (Supplemental Figure 1, B–H). Notably, *Kcnt2* expression levels (Figure 1B) exceeded those of other tested K^+^ channels, underscoring the predominant role of Slick in cardiac fibroblast physiology.

Next, we assessed whether the expected Slick-mediated net K^+^ outflow affects cytosolic K^+^ ([K^+^]_cyto_) in live RCFs/CMFs using the genetically encoded fluorescence resonance energy transfer–based (FRET-based) K^+^ indicator GEPII 1.0 NES lc-LysM (GEPII 1.0) (38). This indicator incorporates a bacterial K^+^-binding protein (Kbp) flanked by mseCFP (cyan) and cpV (yellow) fluorescent protein (YFP). Interaction of [K^+^]_cyto_ with Kbp eventually results in energy transfer from mseCFP to FRET (YFP) signal, which can be visualized under a fluorescent microscope (38). GEPII 1.0 has been demonstrated to function effectively in both primary cells (38–40) and multiple cell lines tested (41, 42). Again, we showed that GEPII 1.0 was functionally expressed in RCFs and CMFs of both genotypes, as evidenced by reciprocal changes of CFP and FRET signals observed in real-time response to increasing extracellular K^+^ ([K^+^]_ex_) concentrations ranging from 1 to 300 mM (Supplemental Figure 1, I–L). Given the [Na^+^]_i_ sensitivity of the Slick channel, we next applied monensin (Na^+^-permeable ionophore) to induce Na^+^ uptake and thereby an increase in intracellular Na^+^ ([Na^+^]_i_) (43). Consequently, a small but significant decline in the normalized average FRET/CFP ratio signal was observed in WT RCFs (Figure 1, C and D) and CMFs (Figure 1, E and F) compared with respective KO cells, suggesting a role of Slick in regulating the Na^+^-induced K^+^ efflux, whereby it functionally affects [K^+^]_cyto_ dynamics in these cells.

To investigate the electrophysiological properties of RCF and CMF in the presence and absence of Slick channels, we performed whole-cell patch-clamp recordings. To this end, niflumic acid (NFA), a previously validated Slick channel opener (44, 45), was applied to evaluate its effect on Slick-mediated outward current components (I_Slick_). In WT RCFs, NFA treatment resulted in a pronounced increase in outward I_Slick_ current density, whereas NFA did not alter current recordings performed in KO RCFs at voltages ranging from −80 mV to 100 mV (Figure 1, G–J). In WT CMFs, we consistently observed a small increase in outward current density in response to NFA, indicative of I_Slick_, while in KO CMFs, the application of NFA unexpectedly repressed whole-cell current density (Figure 1, G and K–M). This phenomenon may be attributed to the lower expression of Slick transcripts in cultured CMFs compared with freshly isolated RCFs (Figure 1B). Alternatively, it might also result from inhibition of NFA-sensitive Cl^–^ currents, potentially mediated by TMEM16A (46, 47), a Ca^2+^-activated Cl^–^ channel known to be highly expressed in myo-/fibroblasts (48, 49). Given that NFA also inhibits TMEM16A, the resulting decrease in outward Cl^–^ current may counteract I_Slick_. This effect might also explain the rather small increase in current density in WT CMFs. Given that KO CMFs lack Slick and thus outward-directed I_Slick_, they demonstrated an unexpected decrease in current density. Furthermore, to validate the role of Slick in Na^+^-induced K^+^ efflux, as previously demonstrated by FRET-based [K^+^]_cyto_ measurements (Figure 1, E and F), we applied monensin during patch-clamp recordings. In KO CMFs, monensin-induced Na^+^ influx led to a marked reduction in outward current amplitude, whereas this decrease was significantly attenuated in WT CMFs, likely due to simultaneous Slick-dependent K^+^ efflux (Supplemental Figure 1, M–Q).

In summary, these live-cell imaging results and patch-clamp recordings provide strong evidence of functional Slick channels at the plasma membrane of RCFs and CMFs that regulate the K^+^ movement from intracellular to extracellular spaces.

### Loss of Slick in RCFs and CMFs disrupts intracellular Ca^2+^ ([Ca^2+^]_i_) dynamics.

**Figure 2:**
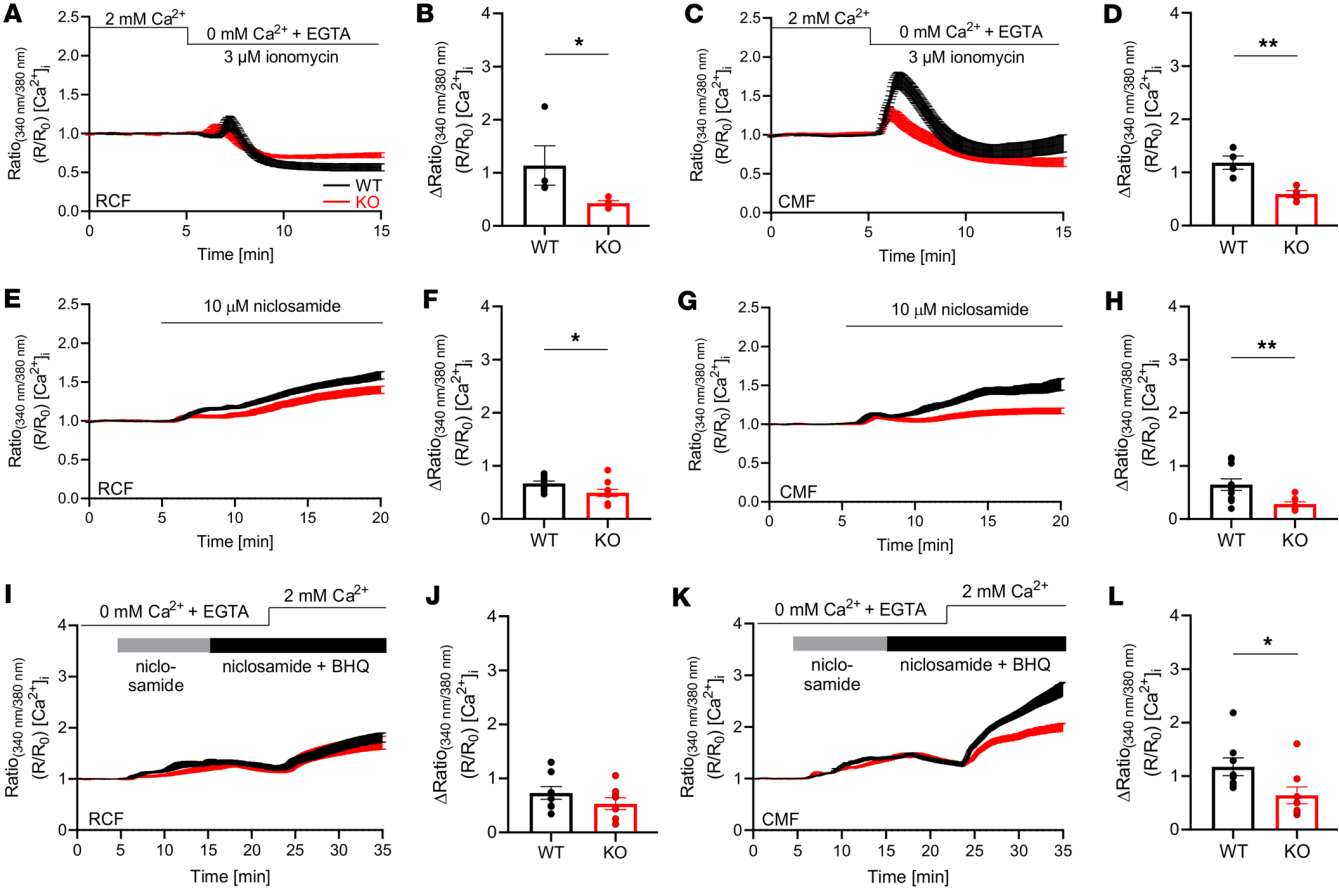
Loss of Slick in RCFs and CMFs disrupts [Ca^2+^]_i_ dynamics. (**A**) Normalized average 340 nm/380 nm ratios over time in RCFs during [Ca^2+^]_ex_ depletion. (**B**) Δ [Ca^2+^]_i_ (R_max_ – R_min_) in WT and KO RCFs. Mann-Whitney *U* test, *P < 0.05 (*n* = 4 hearts per genotype). (**C**) Normalized average 340 nm/380 nm ratios in CMFs during [Ca^2+^]_ex_ depletion. (**D**) Δ [Ca^2+^]_i_ in WT and Slick KO CMFs. Two-tailed unpaired Student’s *t* test, **P < 0.01 (*n* = 4 hearts per genotype). (**E**) Normalized average 340 nm/380 nm ratios in RCFs during niclosamide treatment. (**F**) Δ [Ca^2+^]_i_ (R_20min_ – R_5min_) in WT and KO RCFs. Two-tailed unpaired Student’s *t* test, *P < 0.05 (*n* = 10 hearts per genotype). (**G**) Normalized average 340 nm/380 nm ratios in CMFs during niclosamide treatment. (**H**) Δ [Ca^2+^]_i_ in WT and Slick KO CMFs. Mann-Whitney *U* test, **P < 0.01 (*n* = 10 hearts per genotype). (**I**) Normalized average 340 nm/380 nm ratios during SOCE in RCFs. SOCE was induced by ER Ca^2+^ depletion with BHQ, followed by re-addition of 2 mM [Ca^2+^]_ex_. Niclosamide was applied to open Slick channels 5 minutes after baseline recording. (**J**) Δ SOCE (R_max_ – R_min_ during Ca^2+^ re-addition) in WT and Slick KO RCFs. Two-tailed unpaired Student’s *t* test (*n* = 8 hearts per genotype). (**K**) Normalized average 340 nm/380 nm ratios during SOCE in CMFs. (**L**) Δ SOCE in WT and Slick KO CMFs. Two-tailed unpaired Student’s *t* test, *P < 0.05 (*n* = 8 hearts per genotype).

K^+^ channels have been shown to modulate cellular excitability and [Ca^2+^]_i_ dynamics through multiple mechanisms. These include affecting the influx of extracellular Ca^2+^ ([Ca^2+^]_ex_) and facilitating Ca^2+^ release from intracellular stores, both of which collectively shape [Ca^2+^]_i_ signaling (39, 50, 51). Therefore, we investigated whether and how Slick-dependent K^+^ dynamics affect [Ca^2+^]_i_ in RCFs and CMFs employing Fura-2 (a Ca^2+^-binding fluorescent dye). Initially, we examined the basal [Ca^2+^]_i_ storage content in RCFs and CMFs. We applied ionomycin, a Ca^2+^-permeable ionophore, combined with a gradient change from 2 mM [Ca^2+^]_ex_ buffer to a Ca^2+^-free buffer supplemented by EGTA. Given that this series of experiments was performed with an EGTA-containing Ca^2+^-free buffer, an increase in [Ca^2+^]_i_ reflected the release of Ca^2+^ from intracellular stores rather than entry of Ca^2+^ from the extracellular space. This enabled us to measure the Ca^2+^ content of cardiac fibroblasts’ intracellular stores by calculating the reduction in [Ca^2+^]_i_ following this protocol. Indeed, we observed a transient increase in [Ca^2+^]_i_ in both RCFs and CMFs, likely reflecting Ca^2+^ release from intracellular stores such as the ER. The subsequent drop was indicative of an ionomycin-mediated Ca^2+^ leakage from the cell, reflecting the total [Ca^2+^]_i_ storage capacity, which was significantly higher in WT compared with KO RCFs (Figure 2, A and B) and CMFs (Figure 2, C and D). This indicates that Slick deletion interferes with ER and total [Ca^2+^]_i_ storage in RCFs and CMFs in a reproducible manner.

To investigate [Ca^2+^]_i_ dynamics upon mimicking Slick channel activation, we applied niclosamide, previously described as a selective K_Na_ channel opener (52, 53). Although niclosamide reproducibly activates Slack channels in CMs (40), its effects on Slick channels remain ambiguous. To address this, we employed HEK293 cells cotransfected with *pcDNA3.1-Slick* and *pcDNA3.1-mRuby3*. The inclusion of *pcDNA3.1-mRuby3* not only served as negative control (NC) but also facilitated the identification of successfully Slick-cotransfected cells via its red fluorescence. Immunofluorescence staining confirmed the expression of Slick protein in red fluorescent protein–positive cells within the cotransfected group, whereas Slick was not detectable in the NC group (Supplemental Figure 2A), confirming our Western blot analysis of protein lysates obtained from these cells (Supplemental Figure 2B). At the basal level, cells overexpressing Slick exhibited a relatively lower membrane potential, likely due to an increased K^+^ “leakage” resulting from higher Slick expression (Supplemental Figure 2C). After niclosamide treatment, [K^+^]_cyto_ efflux was significantly increased in Slick-overexpressing cells compared with NC cells, expressing the FRET-based [K^+^]_cyto_-indicator GEPII 1.0 (Supplemental Figure 2, D and E). Consistent with these K^+^ imaging experiments, patch-clamp recordings showed that niclosamide induces a pronounced K^+^ outward current in HEK293 cells overexpressing Slick, and, importantly, that the current amplitudes were significantly higher compared with NC cells (Supplemental Figure 2, F–J). These findings support a role for niclosamide in modulating the activity of Slick.

Upon validating this effect in Slick-proficient HEK293 cells, we applied niclosamide to investigate [Ca^2+^]_i_ dynamics in RCFs and CMFs. This approach allowed us to assess the differential effects of Slick channel activation on [Ca^2+^]_i_ regulation. In WT cells, this treatment resulted in a rather small and transient increment, potentially due to Ca^2+^ released from ER to the cytosol, followed by a steady increase in [Ca^2+^]_i_. The latter response was largely attenuated in KO cells (Figure 2, E–H). This observation prompted us to examine potential origins of the observed Ca^2+^ signals induced by niclosamide in more detail. [Ca^2+^]_i_ in nonexcitable cells, particularly myo-/fibroblasts, rises due to the actions of store-operated calcium entry (SOCE) (54–56). SOCE, mediated by a Ca^2+^ influx through Ca^2+^-release–activated Ca^2+^ (CRAC, aka Orai) channels, is initiated by a preceding ER Ca^2+^ depletion. To determine whether SOCE is the main pathway through which Slick affects Ca^2+^ signaling in RCFs and CMFs, we used 2-Di-t-butyl-1,4-benzohydroquinone (BHQ), an effective inhibitor of sarco-endoplasmic reticulum Ca^2+^-ATPase (SERCA), to deplete ER Ca^2+^ stores (57). Subsequently, cells were superfused with a buffer containing 2 mM [Ca^2+^]_ex_, allowing Ca^2+^ entry via SOCE as observed in different cell types (56, 58, 59). On top of that, we opened Slick with niclosamide to identify the Slick-dependent component of SOCE in both cell types. A schematic illustrating the experimental procedure is shown in Supplemental Figure 3A. Accordingly, Slick-proficient cells exhibited a higher magnitude of Ca^2+^ signals than KO cells via SOCE, an effect that reached significance in CMFs but not in RCFs (Figure 2, I–L). To further characterize the SOCE pathway in CMFs, we utilized GSK7975A, a pan-inhibitor of all 3 Orai protein family members, although it predominantly inhibits Orai1 and has only minor effects on Orai3 (60). Treatment with GSK7975A reduced SOCE signals in WT cells compared with KO levels (Supplemental Figure 3, B and C). Importantly, we excluded the possibility that the observed difference in SOCE levels between WT and KO CMFs originates from altered ER Ca^2+^ release capacity upon activation of Slick by niclosamide. This conclusion is supported by a comparable [Ca^2+^]_i_ increase observed under Ca^2+^-free buffer perfusion in both genotypes (Supplemental Figure 3D). Moreover, transcript levels of the main ER Ca^2+^ release channels, ryanodine receptor and inositol 1,4,5-trisphosphate receptor (IP_3_R) subtypes, did not differ between WT and KO CMFs (Supplemental Figure 3, E–J).

To gain insights into the attenuation of SOCE in KO CMFs, we analyzed the expression profiles of key SOCE regulators, namely stromal interaction molecules (STIM) and Orai channel subtypes. The transcript levels of STIM1 and STIM2 did not differ between genotypes (Supplemental Figure 3, K and L). However, we observed a shift in the expression pattern of Orai subtypes, with an increased Orai3/Orai1 ratio in KO CMFs at both transcript and protein levels (Supplemental Figure 3, M–O). These data imply that the formation of heteromultimeric Orai1-Orai3 is favored in the absence of Slick and that the altered Orai stoichiometry could, in turn, ensure a graded attenuation of SOCE regulation and/or activity in KO CMFs (61).

Lastly, since niclosamide is a known mitochondrial uncoupling agent capable of inducing mitochondrial Ca^2+^ release (62), it remained unclear whether niclosamide-induced mitochondrial dynamics might confound our SOCE results. To address this, we performed oxygen consumption rate (OCR) analysis under basal conditions and after niclosamide treatment. No significant differences in mitochondrial respiration were observed between WT and Slick KO CMFs under baseline or niclosamide-treated conditions (Supplemental Figure 3, P–R). Furthermore, tetramethylrhodamine methyl ester (TMRM) measurements indicated no alterations of mitochondrial membrane potential (Δφ_mito_) upon niclosamide treatment in either genotype (Supplemental Figure 3, S–U). These findings suggest that neither Slick deletion nor niclosamide treatment significantly affects mitochondrial function or Δφ_mito_, ruling out that mitochondrial dynamics contributed to the SOCE readouts.

In summary, Slick deficiency is associated with reduced ER Ca^2+^ content under basal conditions. Pharmacological activation of Slick channels by niclosamide leads to an increased [Ca^2+^]_i_ in both WT RCFs and CMFs. Furthermore, we provide compelling evidence — having systematically excluded multiple confounding factors — that the observed effects stem from alterations in SOCE, primarily driven by changes in Orai channel composition rather than STIM protein expression. Specifically, Slick deletion leads to an increased Orai3-to-Orai1 expression ratio, which is functionally associated with reduced SOCE activity.

### Global Slick deletion attenuates fibrogenesis, stabilizes electrophysiological properties, and preserves left ventricular function after I/R injury.

**Figure 3:**
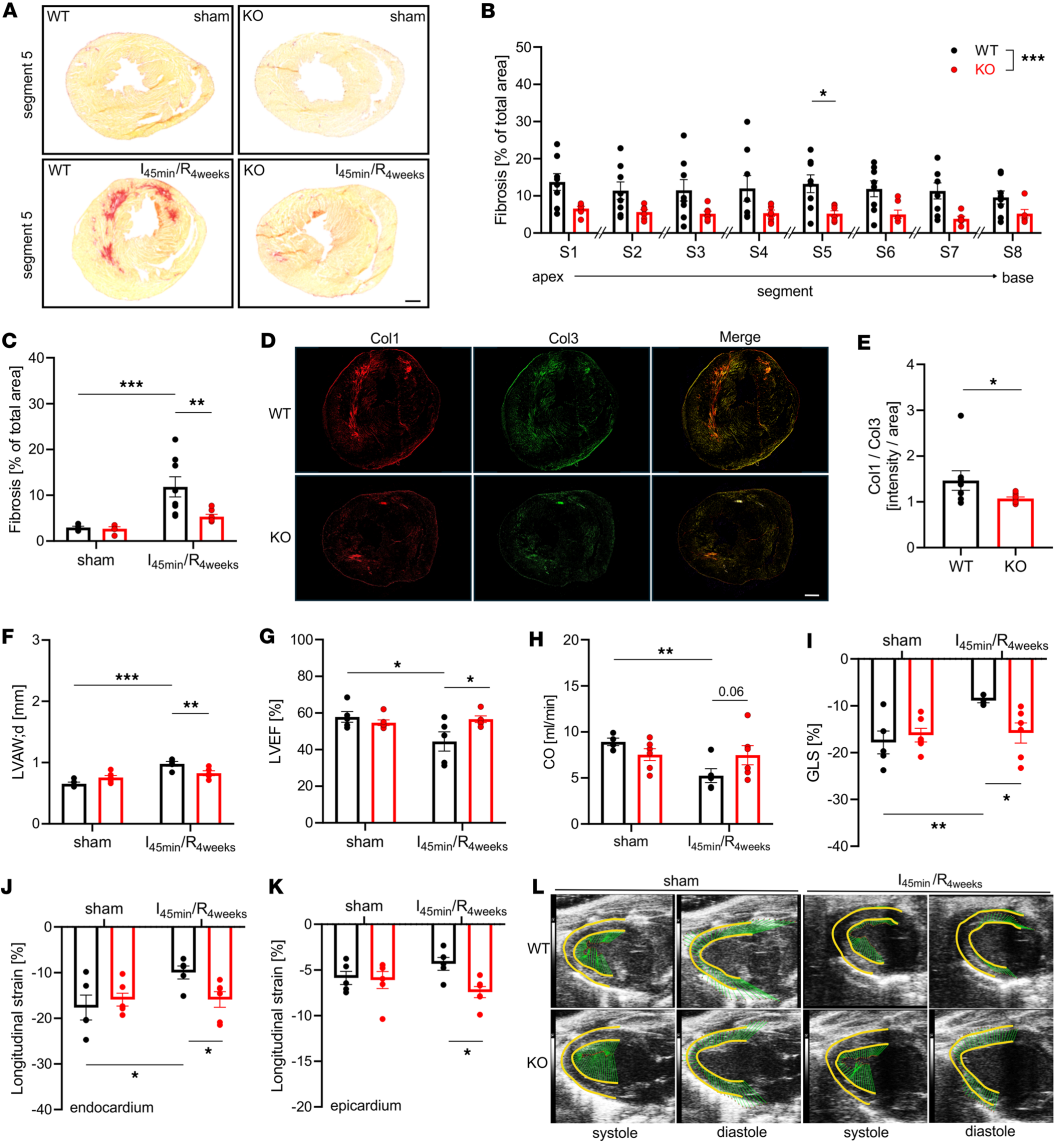
Global Slick deletion attenuates fibrogenesis and preserves LV function after I/R injury. (**A**) Representative Sirius red staining of myocardial fibrosis (red) in WT and KO hearts at segment 5 (S5) after sham or I_45min_/R_4weeks_. Scale bar: 500 μm. (**B**) Analysis of fibrosis (percentage of total area) from S1 to S8 within the affected area (covering all heart tissue affected by the ligature). Two-way ANOVA with Šidák’s multiple-comparison test, ***P < 0.001. (**C**) Average cardiac fibrosis per section. Two-way ANOVA with Tukey’s multiple-comparison test showed increased fibrosis in I_45min_/R_4weeks_ WT compared with sham WT (***P < 0.001) and I_45min_/R_4weeks_ KO (**P < 0.01), with no difference between sham groups (*n* = 5 sham WT, *n* = 5 sham KO, *n* = 8 I_45min_/R_4weeks_ WT, *n* = 7 I_45min_/R_4weeks_ KO). (**D**) Representative polarized light images (S5) from Sirius red–stained sections after I_45min_/R_4weeks_. Red birefringence indicates collagen I, and green indicates collagen III. Scale bar: 500 μm. (**E**) Quantification of the collagen I/III (Col1/Col3) ratio. Mann-Whitney *U* test, *P < 0.05. (**F**) Diastolic left ventricular anterior wall thickness (LVAW;d) measured by M-mode echocardiography (***P < 0.001; **P < 0.01). (**G**) LVEF measured by B-mode imaging, *P < 0.05. (**H**) Cardiac output (CO) measured by B-mode imaging, **P < 0.01. (**I**) Global longitudinal strain (GLS) measured in the parasternal long axis view using speckle-tracking (*P < 0.05; **P < 0.01). (**J** and **K**) Endocardial and epicardial strain analyses in both sham and I_45min_/R_4weeks_ hearts. (**L**) Representative B-mode echocardiography images of sham and I_45min_/R_4weeks_ hearts during systole and diastole. Epicardial and endocardial borders are indicated in yellow. Two-way ANOVA with Tukey’s multiple-comparison test was used for echocardiographic analyses (**F**–**K**) (*n* = 5 sham WT, *n* = 6 sham KO, *n* = 5 I_45min_/R_4weeks_ WT, *n* = 5 I_45min_/R_4weeks_ KO).

To investigate the function of Slick in murine hearts after I_45min_/R_4weeks_ injury, we performed a closed-chest I/R surgery utilizing global Slick KO mice and their littermate controls and assessed chamber remodeling and, in particular, pathological features of fibrosis, (i.e., the accumulation of fibroblasts and the deposition of ECM proteins). A significant fibrotic response of the myocardium was detected in I_45min_/R_4weeks_ groups. Accordingly, collagen fibers, stained by Sirius red, appeared as extensive red areas throughout the myocardium, whereas the respective sham groups remained unstained (Figure 3A). However, the total amount of fibrosis was significantly reduced in KO hearts, indicating that the fibrotic response requires functional Slick channels. Importantly, these apparently profibrotic actions of Slick were consistently observed across each section of the heart distal to the ligature (Figure 3, B and C). In the polarized light images, WT hearts exhibited more collagen I deposition, as reflected by a higher collagen I to collagen III ratio (Figure 3, D and E). This suggests that WT hearts develop a stiffer collagen matrix, which may contribute to reduced left ventricular (LV) compliance and impaired chamber function (63).

Consistent with fibrotic remodeling in post-MI hearts, our ECG recordings during the early reperfusion phase (i.e., I_45min_/R_1week_) revealed features of impaired ventricular electrophysiology (Supplemental Figure 4, A and B). Compared with the basal group, I_45min_/R_1week_-injured hearts exhibited a significantly prolonged QRS interval and a reshaped QRS complex characterized by attenuated R-wave amplitude (Supplemental Figure 4, C and D), indicating slowed ventricular conduction and compromised ventricular depolarization, respectively (13). Moreover, prolonged QTc and JT intervals suggest that ventricular repolarization was impaired as well (64) (Supplemental Figure 4, E and F). Although no significant differences were observed between genotypes in these parameters, WT hearts displayed more pronounced ST-segment depression and greater T-wave inversion than KO hearts (Supplemental Figure 4, G and H). Both alterations are indicative of postischemic electrical remodeling, potentially linked to enhanced fibrotic disruption (13). The observed electrocardiographic abnormalities may in part stem from increased myocardial fibrosis in WT hearts, which likely impairs electrical signal propagation and reduces ventricular compliance, thereby exacerbating electrical instability during the repolarization phase (13).

We next evaluated chamber morphology and function by applying noninvasive echocardiography measurements on I_45min_/R_4weeks_ hearts. Importantly, there was no difference in heart rate between genotypes or between I/R and sham groups during echocardiographic analysis, thus eliminating an important confounder, as heart rate changes affect cardiac function (Supplemental Figure 4I). Using M-mode analysis, WT hearts subjected to I_45min_/R_4weeks_ injury displayed a significantly thickened LV anterior wall (LVAW) compared with sham controls. In the diastolic phase, the thickness of the LVAW was significantly reduced when Slick was deleted (Figure 3F), consistent with a milder progression of muscle fibrosis in KO hearts (Figure 3, A–C). Next, B-mode analysis of cardiac cycles in the parasternal long axis view was combined with speckle tracking to objectively quantify global and local myocardial deformation. Using this feasible and reproducible mode, LV ejection fraction (LVEF) and cardiac output, which represent a central measure of LV function, were significantly impaired in WT hearts after I_45min_/R_4weeks_, whereas these parameters remained largely intact in KO hearts (Figure 3, G and H). Next, the regional damage to LV segments was probed by tracing the wall motion through the cardiac cycle (Supplemental Figure 4J). Global longitudinal strain, which expresses longitudinal shortening as a proportion of baseline length, was improved throughout the entire LV (Figure 3I) in both the endocardial and epicardial surface area analysis (Figure 3, J and K) in the absence of Slick. This comprehensive assessment revealed that WT hearts subjected to I_45min_/R_4weeks_ injury exhibited a diminished longitudinal myocardial shortening (magnitude of deformation) and a regional desynchrony regarding the direction of wall motion, indicative of a significant impairment (Figure 3L). However, radial deformation, that is, the thickening and thinning motion of the myocardium in different segments of the anterior and posterior walls of the LV, was not different between Slick-proficient and Slick-deficient post-I/R hearts (data not shown).

To further assess the potential myocardial growth in response to the I_45min_/R_4weeks_ injury, the heart weight to tibia length ratio (HW/TL) was measured as a hallmark of cardiac hypertrophy (34). There was no significant difference in the HW/TL ratio between the genotypes at baseline and after I_45min_/R_4weeks_ exposure (Supplemental Figure 4K). Consistently, LV mass (Supplemental Figure 4L) and CM cross-sectional area enlargement were similar in both genotypes after I_45min_/R_4weeks_ (Supplemental Figure 4, M and N). Together, these results indicate that Slick, in an adverse manner, affects global LV function and multiple wall deformation parameters, including global longitudinal strain, a long-term risk parameter of cardiovascular morbidity and mortality, while the hypertrophic enlargement of post-I_45min_/R_4weeks_ hearts develops independently of Slick.

### Cell-specific ablation of CMF Slick channels attenuates fibrogenesis and LV dysfunction upon I_45min_/R_4weeks_ injury.

So far, our in vitro experiments have provided substantial evidence for the presence and functionality of Slick channels in RCFs and CMFs. Other studies have previously identified functional Slick channels in cardiac mitochondria (30) and demonstrated a key role for Slick as mediator of anesthetic preconditioning in ex vivo Langendorff-perfused hearts (29). The latter results suggest that cardiac Slick activity confers cardioprotection against an acute and transient I/R insult via anesthetic preconditioning, whereas in our experimental model based on a 4-week post-MI follow-up analysis, we observed an increase in cardiac damage in the presence of Slick. Given these potentially opposing actions of Slick on cardiac injury and to explain these apparently contradictory results, we next aimed to clarify in which cell type Slick function would promote fibrogenesis and chamber dysfunction after cardiac I/R in vivo.

We first subjected global Slick KO mice and their WT littermate controls to an open-chest surgery with I_30min_/R_2h_. This short-term I_30min_/R_2h_ protocol allowed us to evaluate whether Slick modulates the acute myocardial cell death that results in the loss of CMs (40), an important pathological feature driving muscle fibrosis. After I_30min_/R_2h_, neither the area at risk, defined as cardiac tissue within the vascular territory supplied by the infarct-related (ligated) vessel, nor the infarcted area differed between Slick KO and WT mice (Supplemental Figure 4, O–Q), indicating that Slick has no influence on the acute CM death provoked by I_30min_/R_2h_. It is therefore unlikely that the gradually developing differences in cardiac fibrosis between the 2 genotypes are due to a function of Slick in CMs, suggesting instead that Slick contributes to the profibrotic actions of CMFs via cell-intrinsic mechanisms.

**Figure 4:**
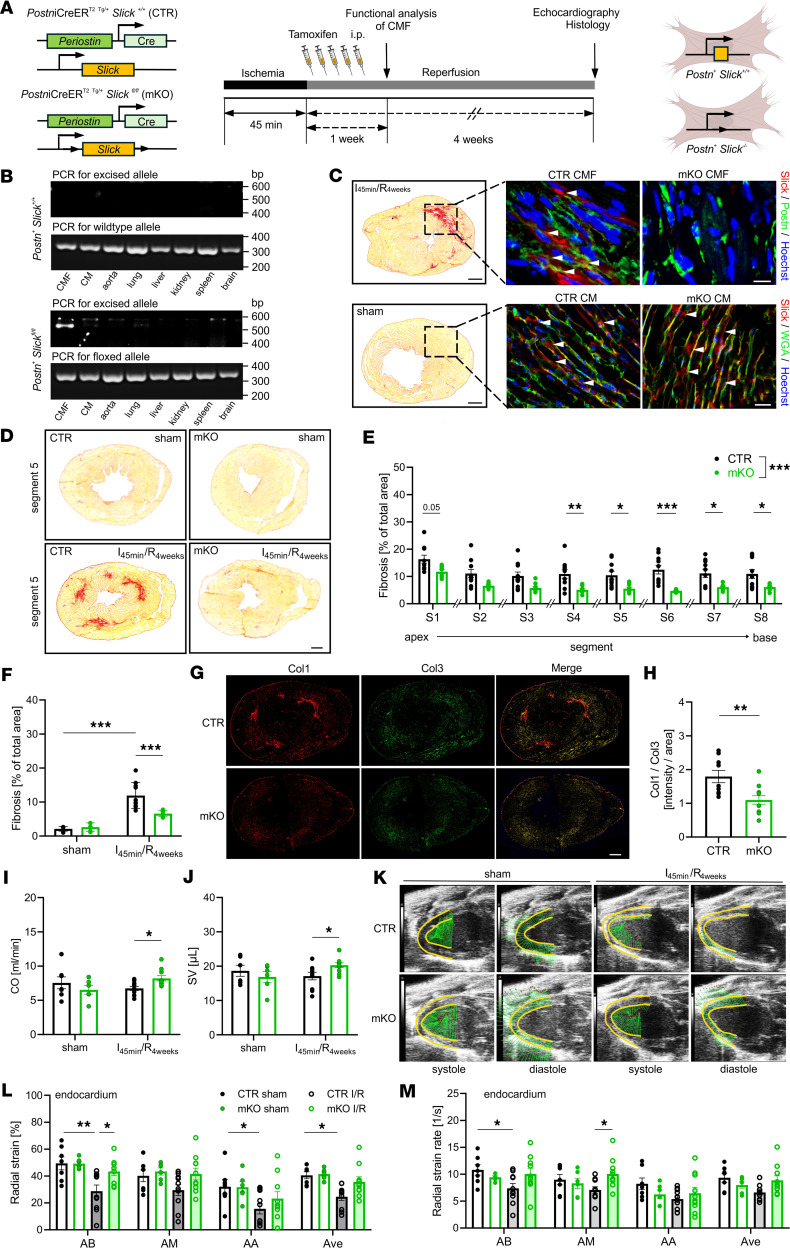
Cell-specific ablation of CMF Slick channels attenuates fibrogenesis and LV dysfunction after I_45min_/R_4weeks_ injury. (**A**) CMF-specific Slick KO mutants were generated utilizing a tamoxifen-activated Cre expressed under control of the *Postn* promoter, ablating Slick in Postn^+^ CMFs. Mice underwent 45 minutes of closed-chest I/R followed by 1 or 4 weeks of reperfusion. Tamoxifen was administered i.p. for 5 consecutive days starting on postoperative day 1. CMFs were isolated after 1 week for functional analyses. Echocardiography and fibrosis quantification were performed after 4 weeks. (**B**) PCR validation of tissue-specific recombination. The excised Slick allele (L1) was detected exclusively in CMFs from mKO mice but not in other tissues or CTR samples. (**C**) Immunofluorescence staining of Slick (red) and periostin (green). Colocalization was observed in fibrotic CTR hearts but absent in mKO hearts after I/R. Scale bars: left, 500 μm; upper right: 10 μm; lower right: 25 μm. (**D**) Representative Sirius red staining of myocardial fibrosis in CTR and mKO hearts after sham or I_45min_/R_4weeks_. (**E**) Segmental fibrosis quantification. Two-way ANOVA analysis with Šidák’s multiple-comparison test, ***P < 0.001. (**F**) Overall average fibrosis across S1–S8. Two-way ANOVA with Tukey’s multiple-comparison test, ***P < 0.001 (*n* = 5 sham CTR, *n* = 5 sham mKO, *n* = 10 I_45min_/R_4weeks_ CTR, and *n* = 9 I_45min_/R_4weeks_ mKO). (**G**) Representative polarized light images (S5). Scale bar: 500 μm. (**H**) Collagen I/III ratio in I_45min_/R_4weeks_ hearts. Two-tailed unpaired Student’s *t* test, **P < 0.01. (**I** and **J**) Cardiac output (CO) and stroke volume (SV), assessed by B-mode echocardiography, *P < 0.05. (**K**) Representative B-mode images during systole and diastole in sham and I_45min_/R_4weeks_ hearts. (**L** and **M**) Radial strain and strain rate analysis in parasternal long axis view. Echocardiographic data (**L** and **M**) were evaluated by Tukey’s multiple-comparison test (*n* = 7 sham CTR, *n* = 6 sham mKO, *n* = 10 I_45min_/R_4weeks_ CTR, *n* = 9 I_45min_/R_4weeks_ mKO).

To further substantiate Slick’s potential role in CMFs in vivo, we generated a CMF-specific Slick KO (mKO) mouse line that relies on a tamoxifen-inducible Cre-recombinase under the control of the periostin (*Postn*) promoter and its recognition (loxP) sites flanking exon 22 of the Slick gene (15, 33). This approach ensured that Slick deletion occurred selectively only in the presence of *Postn*, a matricellular protein predominantly expressed in CMFs under pathophysiological conditions such as I/R injury or angiotensin II infusion (15, 34). On the other hand, nearly all RCF-derived CMFs express *Postn*, ensuring consistency of the CMF subpopulation analyzed between our in vitro and in vivo studies. The mKO mice (*Postni-Cre^Tg/+^* *Slick^fl/fl^*) and their respective littermate controls (*Postni-Cre^Tg/+^* *Slick^+/+^* or CTR) were subjected to the identical I_45min_/R_4weeks_ injury model previously used to assess WT and global Slick KO mice (Figure 4A). After I_45min_/R_4weeks_ surgery, tamoxifen was administered i.p. for 5 consecutive days to induce Cre-recombination in CMFs, which are highly activated during this time of the progressively developing remodeling response (10). The specificity of Cre-recombinase activity was confirmed by detecting the excised Slick DNA allele exclusively in CMFs (Figure 4B). Additionally, by using validated anti-Slick antibodies, we confirmed that the targeted ablation of the Slick channel protein was restricted to fibrotic heart regions, where *Postn*-positive CMFs are particularly prevalent (Figure 4C).

Consistent with previous findings from WT and global Slick KO, a significant increase in fibrosis was observed in CTR hearts, while fibrogenesis provoked in mKO hearts was markedly diminished. This reduction in the amount of fibrosis was evident in all cardiac segments assessed from the apex to the base, that is, close to the ligation site (Figure 4, D–F). mKO hearts exhibited a less rigid collagen matrix compared with CTR hearts, as indicated by polarized light imaging (Figure 4, G and H).

To assess accompanying alterations in cardiac function, we carried out conventional and strain echocardiographic imaging. Again, the heart rate between genotypes did not differ during these measurements (Supplemental Figure 5A). Compared with the sham group, a decline in LVEF was observed in both genotypes that reached significance only for the CTR animals after I_45min_/R_4weeks_ injury (Supplemental Figure 5B). Although differences in post-I/R LVEF between CTR and mKO hearts were not observed, cardiac output and stroke volume in mKO hearts presented with significantly better values (Figure 4, I and J), indicative of a partially recovered cardiac performance in the absence of Slick in CMF. Because the adverse tissue remodeling upon I_45min_/R_4weeks_ is an event with locally varying degrees of impairment, we divided the heart into anterior and posterior walls, which were further subdivided into apex, mid, and base regions (Supplemental Figure 4J). LV motion analysis of the anterior wall in which the fibrotic scar predominantly forms due to left coronary artery ligation (i.e., where *Postn*i-Cre–mediated activity in CMFs led to Slick ablation) was impaired in CTR compared with mKO hearts, with respect to endocardial radial strain and strain rates due to the I/R injury. Similar trends were observed in the epicardial layer (data not shown as radial strain parameters across the myocardial wall were largely consistent). This functional decline in myocardial thickening and thinning, which is visualized by the length and direction of vector arrows, was partially preserved in the absence of Slick (Figure 4, K–M). In contrast to WT and global Slick KO post-MI hearts (Figure 3), local longitudinal strain and strain rates in the anterior or posterior walls were not different between CTR and conditional mKO hearts (data not shown). These findings suggest that targeting Slick in CMFs mitigates fibrosis and partially restores cardiac function after I/R injury.

Consistent with our analyses of WT versus global Slick KO, no significant difference in cardiac hypertrophy between CTR and mKO genotypes, as indicated by the comparable LV mass and cross-sectional area assessed by wheat germ agglutinin staining, could be observed (Supplemental Figure 5, C–E). Despite the excessive fibrosis in CTR hearts (Figure 4, D–F), a small but significant decline in the HW/TL ratio in post-I/R CTR compared with mKO hearts was detected (Supplemental Figure 5F). This decrease in the HW/TL ratio might reflect CM loss resulting from harmful overactivation of fibrotic signaling pathways (65). Indeed, a significantly higher number of TUNEL-positive CMs was observed in CTR versus mKO hearts (Supplemental Figure 5, G and H), suggesting that fibrosis and the provoked apoptotic CM cell death correlated closely with each other. Hence, CM survival was improved when Slick-dependent fibrogenesis was attenuated in I_45min_/R_4weeks_-exposed hearts.

Given the crucial role of immune cells, particularly T cells and macrophages, in chronic cardiac remodeling after MI (66), we sought to determine whether the chronic immune response is altered in mKO hearts. Indeed, we observed a significant reduction in the number and frequency of CD68^+^ macrophages (67) in I_45min_/R_4weeks_ hearts, particularly along the fibrotic scar region. In contrast, CD3^+^ T cells (68) comprised a smaller fraction of the total immune cell population and showed no significant difference between genotypes (Supplemental Figure 5, I–N).

To further explore whether and how Slick-deficient CMFs influence macrophage function, such as recruitment, activation, proliferation, or maintenance, we assessed the expression of cytokine- or chemokine-related transcripts in both CMFs and whole hearts after I/R (Supplemental Figure 5, O–S). Although no significant changes in transcript levels were detected in CMFs, a notable upregulation of *Ccl5* (encoding CCL5), a factor known to mediate the recruitment of proinflammatory macrophages to infarcted myocardium (69), was found in CTR hearts (Supplemental Figure 5R). In addition, CTR hearts exhibited increased expression of proinflammatory cytokines, among which *Il1b* (encoding IL-1β) was significantly elevated (Supplemental Figure 5, T–V).

Together, these findings suggest that Slick ablation in CMFs attenuates the proinflammatory immune response in post-MI hearts by limiting macrophage recruitment and inflammatory cytokine expression. This effect does not appear to arise from direct changes in CMF chemokine output but may instead reflect a broader suppression of profibrotic signaling and fibrotic-mediated cell death in the infarcted myocardium in mKO hearts.

### Loss of Slick attenuates the proliferation capability and diminishes the expression of fibrotic genes in CMF^I/R^ ex vivo.

So far, our data from 2 Slick mutant mouse models have suggested that Slick contributes to the profibrotic actions of CMFs in the post-I_45min_/R_4weeks_ myocardium in vivo. Moreover, loss of Slick in cultured CMFs established from healthy hearts disrupted the [Ca^2+^]_i_ dynamics of both cell types (Figure 2). To further deepen our understanding of how Slick function might influence CMFs upon I/R injury, we isolated and assessed cardiac fibroblasts after I_45min_/R_1week_ and refer to these cells as CMF^I/R^ to distinguish them from CMFs obtained in vitro by cultivating RCFs over time. We chose a reperfusion time of 7 days for the isolation of the cells because at this time, CMFs in the myocardium are usually highly active (9).

**Figure 5:**
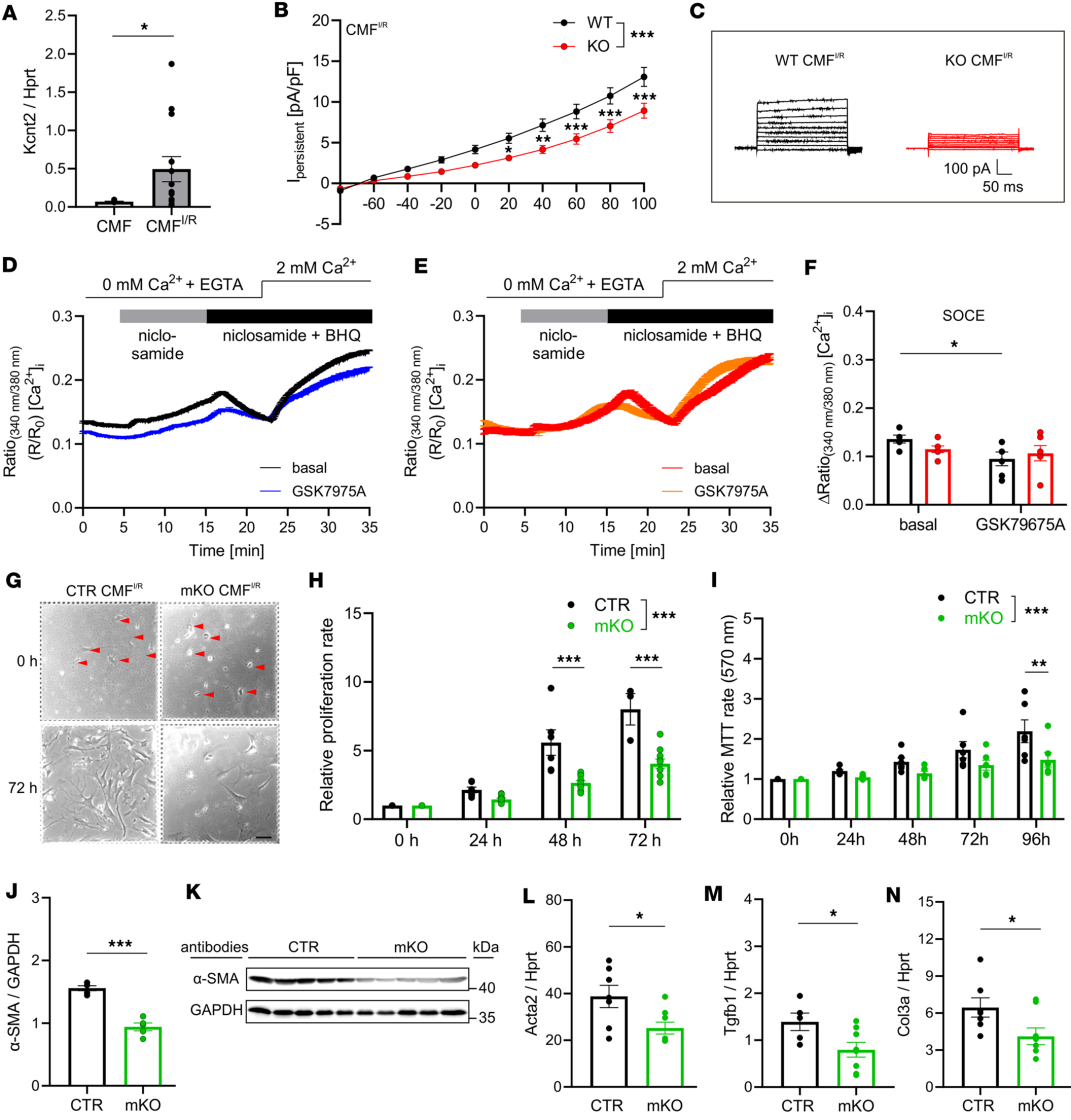
Loss of Slick attenuates proliferation and fibrotic gene expression in CMF^I/R^. (**A**) RT-qPCR analysis of *Kcnt2* expression in cultured CMFs and in CMF^I/R^, normalized to *Hprt*. Mann-Whitney *U* test, *P < 0.05 (*n* = 6 hearts for CMF and *n* = 13 hearts for CMF^I/R^). (**B**) I-V relationships of persistent currents in WT and KO CMF^I/R^. Two-way ANOVA followed by Šidák’s multiple-comparison test, ***P < 0.001 (*n* = 6 hearts per genotype). (**C**) Representative outward current traces in WT and KO CMF^I/R^. (**D** and **E**) SOCE traces in WT and KO CMF^I/R^ treated with DMSO (basal) or GSK7975A. (**F**) ΔSOCE (R_max_ – R_min_ during Ca^2+^ re-addition) in CMF^I/R^. GSK7975A significantly reduced SOCE in WT. Two-way ANOVA followed by Tukey’s multiple-comparison test, *P < 0.05 (*n* = 5 hearts for basal WT; *n* = 6 hearts for all other groups). (**G**) Representative brightfield images (0 h, 72 h) of grid-plate-based proliferation assay. Scale bar: 100 μm. (**H**) Quantitative analysis of proliferation rate over time. Two-way ANOVA with Šidák’s multiple-comparison test, ***P < 0.001 (*n* = 7 CTR and *n* = 11 mKO). (**I**) MTT assay of CMF^I/R^. Two-way ANOVA with Šidák’s multiple-comparison test, ***P < 0.001 (*n* = 6 hearts per genotype). (**J** and **K**) Western blot analysis of α-SMA in CMF^I/R^, normalized to GAPDH. Two-tailed unpaired Student’s *t* test, ***P < 0.001 (*n* = 5 hearts per group). (**L**–**N**) RT-qPCR analysis of profibrotic transcripts, normalized to *Hprt*. (**L**) *Acta2* expression. Mann-Whitney *U* test (*P < 0.05; *n* = 7 CTR, *n* = 8 mKO). (**M**) *Tgfb1* expression. Two-tailed unpaired Student’s *t* test, *P < 0.05 (*n* = 5 CTR, *n* = 8 mKO). (**N**) *Col3a1* expression. Two-tailed unpaired Student’s *t* test (*P < 0.05; *n* = 7 CTR, *n* = 8 mKO).

To confirm the purity of isolated CMF^I/R^, we assessed the expression of established myofibroblast markers as previously mentioned (Supplemental Figure 1A), which revealed robust expression of *Acta2* and *Postn*. Transcripts corresponding to cell-surface and intracellular markers of other cardiac cell types, however, were not detectable (Supplemental Figure 6A). We also examined the transcript levels of relevant K^+^ channels that play critical roles in, for example, controlling the electrical activity under I/R conditions, but could not observe compensatory changes in KO cells (Supplemental Figure 6, B–H). Notably, *Kcnt2* expression in CMF^I/R^ was nearly 10-fold higher compared with CMFs cultured in vitro, which is consistent with the sequencing data of Qian et al. (32), indicating a strong transcriptional upregulation in response to ischemic stress (Figure 5A). Whole-cell patch-clamp recordings confirmed Slick-dependent outward K^+^ currents in WT CMF^I/R^, with KO cells serving as NC (Figure 5, B and C).

To further validate the alterations in SOCE previously observed in vitro, we applied the same Ca^2+^ imaging protocol to CMF^I/R^. Preincubation with the Orai channel inhibitor GSK7975A for 30 minutes significantly reduced basal [Ca^2+^]_i_ levels in WT CMF^I/R^ but not in KO cells (Supplemental Figure 6I). After SOCE stimulation, GSK7975A treatment led to a pronounced attenuation of Ca^2+^ influx in WT CMF^I/R^, while no significant effect was observed in the KO (Figure 5, D–F), supporting a Slick-dependent regulation of SOCE. Importantly, neither ER Ca^2+^ release nor the gene expression of IP_3_R or ryanodine receptor subtypes differed significantly between genotypes (Supplemental Figure 6, J–P), except for *Ip3r2* encoding IP3R2 (Supplemental Figure 6O). The expression level of *Ip3r2*, however, was extremely low, approaching the detection threshold, and thus is likely to be biologically negligible. This suggests that the observed differences in [Ca^2+^]_i_ homeostasis are specific to SOCE alterations. In agreement with the in vitro data, *Stim1* and *Stim2* levels remained unchanged, whereas the *Orai3/Orai1* expression ratio was significantly elevated in Slick-deficient CMF^I/R^.

Taken together, these findings provide strong evidence that Slick deletion modulates SOCE activity in CMF^I/R^, consistent with our in vitro observations. This effect is likely mediated by an altered composition of Orai channel subtypes, rather than by changes in upstream ER Ca^2+^ release mechanisms.

In the next step, we tested the proliferative behavior of Slick-proficient versus Slick-deficient CMF^I/R^ using a grid-based cell-growth assay (34). By tracking individual CMF^I/R^ in the same grid over time, we observed a significant increase in cell numbers. Importantly, at all time points examined, mKO CMF^I/R^ cell counts were markedly reduced compared with CTR (Figure 5, G and H). This difference between both genotypes was further confirmed by evaluating the number of viable CMF^I/R^ over time via the MTT assay (Figure 5I). These findings identified a significant reduction in the proliferative capacity as one possible reason for the attenuated fibrotic response of global and CMF-specific post-I/R hearts lacking Slick.

Additionally, we characterized the activation of CMF^I/R^ by measuring α-SMA. At both transcript and protein levels, α-SMA was increased in CTR versus mKO CMF^I/R^ (Figure 5, J–L). Consistently, we found higher TGF-β and collagen III transcript expression levels in Slick CTR CMF^I/R^ (Figure 5, M and N).

In summary, these findings indicate that Slick function in primary CMF^I/R^ promotes cell proliferation and the fibrotic signaling associated with CMF activation, ultimately leading to accelerated collagen production in vivo. This functional effect is likely mediated through a SOCE-dependent mechanism involving Orai channel remodeling in the presence or absence of Slick.

## Discussion

**Figure 6:**
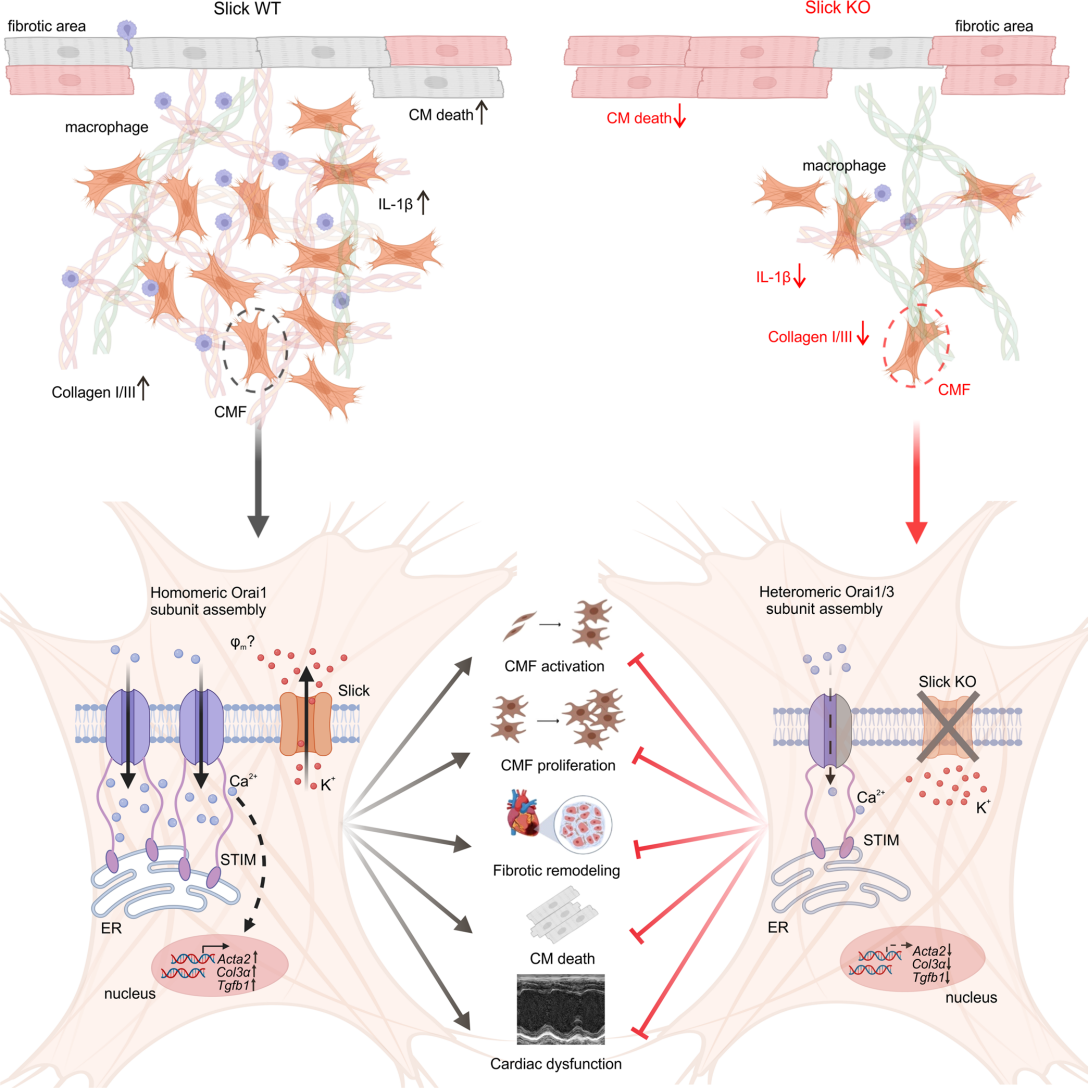
Graphic summary. Slick channels regulate CMF function and post-MI cardiac remodeling. After chronic I/R, WT hearts exhibit increased collagen deposition, inflammatory activity, and CMF activation, contributing to CM loss and LV dysfunction. In contrast, Slick-deficient hearts show reduced fibrosis and inflammation with improved myocardial preservation. At the cellular level, Slick-mediated K^+^ efflux enhances the electrochemical driving force for SOCE and promotes profibrotic transcriptional programs. Loss of Slick reduces Ca^2+^ influx and SOCE efficiency, likely through Orai channel remodeling, thereby limiting CMF activation and proliferation and preserving ventricular structure and function after MI. The figure was partly created using BioRender (license TN29GGWQJZ and OF29GGXG83).

Our study revealed a role of Slick in post-MI cardiac remodeling that we believe was previously unrecognized. As part of our thorough analysis, we demonstrated Slick expression in cultured CMFs and infarction-derived CMF^I/R^ and identified its role in [K^+^]_cyto_ efflux, which may consequently affect SOCE-mediated Ca^2+^ signaling. Furthermore, the absence of Slick in CMFs attenuated cardiac fibrosis, reduced inflammatory activation, limited CM death, and preserved chamber performance after I/R injury. Ex vivo analyses of CMF^I/R^ confirmed diminished proliferation and profibrotic gene expression in Slick-deficient CMFs (Figure 6). Collectively, these findings position myo-/fibroblast Slick channels as key regulators of post-MI fibrogenesis and chamber dysfunction.

Slick channels display pronounced sensitivity to [ATP]_i_, being markedly blocked at 5 mM ATP (28). Coupled with their activation by elevated [Na^+^]_i_, this dual regulation enables integration of metabolic and ionic cues, particularly under ischemic stress characterized by ATP depletion and Na^+^ accumulation, thereby modulating electrical activity (70). Previous work demonstrated that Slick confers anesthetic preconditioning–mediated cardioprotection against transient hypoxia/reperfusion injury (29). Extending this ex vivo finding, our study identified Slick as a regulator of post-MI cardiac remodeling. In contrast to the mitochondria-centered mechanism described for anesthetic preconditioning (30), we demonstrated that Slick modulates profibrotic signaling in CMFs after I/R injury. These results revealed a context-dependent role of Slick in the heart: While Slick in CMs limits acute ischemic injury, its function in RCFs/CMFs promotes maladaptive remodeling and functional decline during prolonged reperfusion. Overall, fibroblast-driven profibrotic effects of Slick appear to outweigh its early cardioprotective actions in CMs.

Our in vitro analysis of Slick-dependent K^+^ dynamics and electrophysiological recordings in RCFs and CMFs confirms functional channel expression and electrophysiological relevance in both cell types. To emulate ischemic-associated [Na^+^]_i_ overload, monensin was applied as a Na^+^ ionophore (43). This intervention elicited a counteractive Slick-dependent outward current in CMFs (Supplemental Figure 1, M–Q), consistent with a compensatory response to Na^+^ influx and supporting a role for Slick in preserving K^+^ homeostasis and maintaining membrane stability under ionic stress. To further probe Slick activity, we applied NFA, a previously reported pharmacological opener of Slick (44). NFA robustly enhanced Slick-mediated outward currents in RCFs, whereas effects in CMFs were modest, and Slick-deficient CMFs displayed reduced outward currents (Figure 1, H–M). This observation may be explained by lower expression of Slick in cultured CMFs compared with RCFs, as indicated by a single-cell RNA-Seq approach (31) and RT-qPCR results (Figure 1B). In addition, NFA inhibits TMEM16A (46, 47), a Ca^2+^-activated Cl^–^ channel highly expressed in cardiac cells (48, 49) and markedly enriched in CMFs relative to RCFs, with expression levels nearly 200-fold higher in CMFs than in RCFs (31). Thus, it is plausible that TMEM16A inhibition may partially offset Slick-mediated K^+^ currents in CMFs. This interpretation remains speculative and requires further validation. In addition to NFA, we employed niclosamide, an anthelmintic drug known to modulate various signaling pathways (71, 72), including K_Na_ channels. Although niclosamide has been shown to activate Slack channels in CM sarcolemma (40), its effect on Slick has not been defined. Here, our data indicate that niclosamide also activates Slick channels (Supplemental Figure 2, D–J). Although off-target activation of Slack in RCFs and CMFs cannot be entirely excluded, this is likely minimal given the predominant expression of Slick over Slack in these cells (31). Consistently, RT-qPCR analysis revealed *Kcnt1* transcripts (encoding Slack) near the detection limit in both RCFs and CMFs (data not shown), supporting the view that Slick represents the principal K_Na_ channel in RCFs and CMFs.

Previous studies have described distinct subcellular localizations for cardiac Slick channels. Smith et al. identified Slick in CM mitochondria and linked it to anesthetic preconditioning–mediated cardioprotection (30). In contrast, our whole-cell patch-clamp recordings in RCFs and CMFs support functional Slick channels at the plasma membrane (Figure 1, H–M). Consistently, OCR measurements and TMRM-based measurements of Δφ_mito_ revealed no differences between Slick-proficient and Slick-deficient CMFs (Supplemental Figure 3, Q–U), arguing against a major mitochondrial contribution in these cells. Moreover, monensin-induced Na^+^ influx (43) elicited a modest but significant Slick-dependent modulation of FRET-based [K^+^]_cyto_ signals (Figure 1, C–F). These results indicate that Slick operates at the plasma membrane of RCFs and CMFs to regulate K^+^ dynamics.

SOCE represents a major source of Ca^2+^ entry pathway in nonexcitable cells and is triggered by ER Ca^2+^ depletion (73), while its amplitude is determined by STIM-Orai coupling and the relative expression of Orai isoforms (Orai1, Orai2, and Orai3) (74). Homomeric Orai1 channels exhibit greater efficiency than heteromeric assemblies (75), and shifts in Orai isoform expression modulate SOCE in cancer cells and macrophages (76, 77). Orai3 overexpression leads to higher formation of Orai1/3 heteromers and attenuates SOCE in pulmonary fibroblasts (61). In diseased human hearts, enhanced SOCE and elevated Orai1 expression correlate with fibrotic remodeling (54), and angiotensin II–mediated upregulation of Orai1 and STIM aggravate maladaptive CMF activation and ECM deposition (78). In our study, Slick deficiency markedly attenuated SOCE in CMFs (Figure 2, K and L), coinciding with an increased Orai3/Orai1 ratio, presumably leading to heteromeric channel formation. However, STIM expression remained unchanged (Supplemental Figure 3, K and L), suggesting that altered Orai composition rather than STIM availability underlies the reduced Ca^2+^ entry and lessened fibrosis after I/R injury.

The reduced SOCE observed in Slick-deficient CMFs may reflect a diminished electrochemical driving force resulting from loss of I_Slick_ current and impaired K^+^ efflux, as both membrane potential and Ca^2+^ gradient critically regulate SOCE (79). Consistent with this concept, measurements of membrane potential based on bis-(1,3-dibutylbarbituric acid)trimethine oxonol (DiBAC_4_(3)) showed that HEK293 cells overexpressing Slick displayed a less depolarized (less positive) plasma membrane than NC cells (Supplemental Figure 2C), supporting this hypothesis. Slick has previously been reported to associate with nonselective voltage-independent Na^+^ leak channels and transient receptor potential Melastatin 3 channels, contributing to ionic homeostasis through spatial proximity (80, 81). Here, we propose that in CMFs, Slick and Orai channels may similarly reside in close spatial proximity, allowing Slick-mediated K^+^ efflux to modulate the local membrane potential and thereby influence SOCE. Loss of Slick could disturb this microdomain regulation, potentially favoring adaptive remodeling of Orai isoform composition in response to altered Ca^2+^ signaling demands. While mechanistically plausible, this hypothesis remains to be experimentally validated.

In vivo, global Slick KO mice subjected to chronic I_45min_/R_4weeks_ exhibited reduced fibrosis and attenuated LV dysfunction compared with controls, indicating that myocardial Slick promotes adverse remodeling. However, it remained unclear whether these effects reflect altered transient CM death and resulting replacement fibrosis or an intrinsic profibrotic role of Slick in CMFs after MI. To clarify this, we performed acute open-chest I_30min_/R_2h_ experiments with 2,3,5-triphenyltetrazolium chloride (TTC) staining and observed comparable infarct size in both genotypes (Supplemental Figure 4, M–O), suggesting that Slick does not influence acute CM death under these conditions. Notably, isoflurane was used during closed-chest I_45min_/R_4weeks_ surgery and has been linked with anesthetic preconditioning (29). In line with this, our unpublished observations indicate reduced anesthetic preconditioning–mediated protection in global KO hearts after transient I/R. However, acute CM damage and chronic fibrotic remodeling involving RCF/CMF represent distinct pathophysiological processes. Our finding suggests that during prolonged reperfusion, the profibrotic actions of Slick in CMFs outweigh any early cardioprotective effects in CMs.

To directly interrogate CMF-specific effects and avoid confounding by CM Slick deletion, we employed a tamoxifen-inducible *Postn*i-Cre mouse line. Periostin (encoded by *Postn*) serves as a highly specific marker in injury-activated CMFs, being minimally expressed in healthy myocardium yet upregulated nearly 200-fold 7 days after MI (14, 15, 34). This injury-restricted and robust induction makes the *Postn* gene promoter a suitable driver for CMF-targeted recombination. CMF-specific Slick ablation significantly reduced fibrosis after I_45min_/R_4weeks_, supporting a CMF-intrinsic role of Slick in post-MI remodeling. Functional analyses revealed partial preservation of LV performance, particularly within the anterior wall, consistent with the region predominantly affected by left coronary artery ligation.

Because CMFs are highly active during the early reparative phase, when tamoxifen-induced recombination occurred, regional preservation of chamber function likely reflects early suppression of CMF activity in this area (Figure 4, L and M). Given the limited 5-day tamoxifen treatment window (14), incomplete recombination of Slick-proficient CMFs may have contributed to residual fibrosis during prolonged reperfusion, explaining the more region-restricted improvement in mKO compared with global KO hearts. Specifically, the mKO hearts exhibited greater improvement in radial chamber function, whereas global Slick KO hearts showed a broader improvement in overall cardiac motion (i.e., longitudinal contractility). Examination of CMF^I/R^ after 1 week revealed reduced α-SMA expression and diminished proliferative capacity in mKO cells. These in vitro features align with attenuated fibrotic remodeling and the improved functional outcome observed in the absence of global or CMF-specific Slick.

CMFs in post-MI hearts represent a heterogeneous population of diverse origins. The majority arise from RCF-derived CMFs, originating from embryonic epicardium (12); additionally contributions from bone marrow–derived fibroblast-like cells and endothelial-to-mesenchymal transition must be taken into account (11). To define Slick expression and function in a controlled setting, we employed cultured RCF-derived CMFs, representing the dominant injury-responsive subpopulation. This approach minimized lineage heterogeneity and enabled more precise interpretation of Slick-dependent mechanisms in these cells. Indeed, Slick was predominantly expressed in RCF-derived CMFs relative to other K^+^ channels (Figure 1B and Supplemental Figure 1, B–H) and exerted a clear modulatory effect on SOCE dynamics (Figure 2).

Although RCF-derived CMFs constitute the predominant subpopulation in post-MI hearts in vitro, the in vivo MI milieu entails complex and integrated profibrotic signaling pathways that cannot be fully recapitulated in vitro. We therefore isolated CMFs from I/R-injured hearts and observed higher expression of *Kcnt2* together with coordinated upregulation of profibrotic genes (Figure 5A and Supplemental Figure 6A), strengthening the association between Slick and post-MI fibrogenesis. Notably, the SOCE regulation and Orai remodeling identified in cultured CMFs were reproducible in CMF^I/R^ (Figure 5, D–F), supporting a conserved mechanism under I/R conditions. To delineate CMF-intrinsic effects, we further employed *Postn*i-Cre mice, in which nearly all RCF-derived CMFs express *Postn* (13). The substantial overlap between RCF-derived and *Postn*^+^ CMFs enabled interpretation of Slick function within the dominant CMF lineage analyzed in this study.

Finally, cardiac K^+^ channels can perturb action potential configuration and thereby modulate arrhythmogenic risk (36). Given the electrical coupling between fibroblasts and CMs, Slick activity residing in RCFs/CMFs could potentially alter adjacent sarcolemmal depolarization and predispose to arrhythmias. However, ECG recordings revealed no electrophysiological differences between WT and global KO mice. After I/R injury, both genotypes exhibited prolonged LV repolarization and impaired conduction, reflected by extended QRS duration, reduced R-wave amplitude, and prolonged QT and JT intervals (Supplemental Figure 4, C–F), yet these changes were comparable between groups. Thus, the profibrotic effects of Slick in post-MI remodeling are unlikely to be primarily mediated by electrical abnormalities.

In conclusion, our study identifies Slick as a key driver of RCF/CMF-mediated fibrogenesis after MI. CMF-specific Slick deletion attenuates profibrotic activation and maladaptive remodeling following chronic I/R injury. Mechanistically, Slick-dependent K^+^ efflux enhances SOCE-mediated Ca^2+^ signaling, thereby promoting key fibroblast functions. Notably, although Slick confers protection against acute CM death during early I/R injury, its profibrotic actions in CMFs dominate during chronic remodeling. These findings highlight the importance of temporal precision when considering Slick as a potential therapeutic target (Figure 6).

This study has several limitations. Although both male and female mice were used for in vitro experiments, the in vivo I/R model was restricted to males. Thus, the profibrotic role of Slick in females remains to be defined. Given the lower incidence of ischemic events in females and the confounding effect of tamoxifen on estrogen receptor signaling (82), we focused on male mice to ensure experimental consistency and interpretability. Future studies using female mouse models and tamoxifen-independent recombination systems will be required to address potential sex-specific effects. In addition, our findings are currently limited to murine models. Validation in human samples will be necessary to determine the clinical relevance of Slick expression and potential genetic variation in ischemic heart disease.

## Methods

Detailed in vivo and in vitro experimental procedures are provided in the Supplemental Methods.

### Sex as a biological variable.

For in vitro experiments, cells were isolated from both male and female mice. For in vivo I/R studies, experiments were performed exclusively on male mice.

### Animals.

Mice were kept on a standard 12-hour light/12-hour dark cycle and had unlimited access to food and water and were housed in a standardized cage system with continuous monitoring of temperature and humidity, maintaining a defined room temperature and humidity conditions. Slick-deficient (*Slick^–/–^*, KO) mice and their corresponding age-matched control littermates (*Slick^+/+^*, WT) were generated as previously described (33). Additionally, conditional mutants specifically lacking Slick in CMFs (*Postn*i-Cre*^Tg/+^* *Slick^fl/fl^*, mKO) and controls from the same litters (*Postn*i-Cre*^Tg/+^ Slick^+/+^*, CTR) were established by crossing transgenic mice expressing Cre-recombinase under the control of the *Postn* promoter (*Postn*i-Cre*^Tg/+^*) with mice carrying floxed Slick gene alleles (*Slick^fl/fl^*). In offspring deriving from these matings, Cre-mediated recombination of the loxP-flanked Slick gene was induced by tamoxifen i.p. (Sigma-Aldrich, T5648) at a dose of 1 mg per day for 5 consecutive days postoperatively (15). In general, I/R surgeries (and sham operations) were carried out in male animals aged 10–16 weeks.

### Isolation of primary RCFs and CMFs.

Murine hearts were isolated after euthanasia. Aorta and atria were carefully removed in ice-cold PBS, and ventricles were dissected into small pieces for further digestion. The digestion buffer consisted of 1 mM collagenase type II (Worthington, LS004177) in a Ca^2+^-free buffer (in mM: 85 Na-glutamate, 60 NaCl, 10 HEPES, 5.6 KCl, 1 MgCl_2_, and 1 BSA; Carl Roth, 8076.2). Cells were separated from undigested heart pieces and purified using a 40 μm strainer (Greiner Bio-One, 542040). After centrifugation at 17*g* for 5 minutes, a uniform single-cell suspension was obtained. Subsequently, cell pellets were collected by centrifugation at 37*g* for 7 minutes and resuspended in culture media consisting of DMEM GlutaMAX (Gibco, 31966-021) supplemented with 10% FBS, 1% insulin-transferrin-selenium (Sigma-Aldrich, I3146), and 1% penicillin-streptomycin (Gibco, 15140-122). Cells were collected immediately after isolation or cultured in an incubator containing 5% CO_2_ and 21% O_2_ at 37°C, pH 7.4, for subsequent experiments.

### Statistics.

Statistical analyses were conducted using GraphPad Prism 10.0. Data are presented as mean ± SEM. The normality of data distribution was assessed using either the Shapiro-Wilk or Kolmogorov-Smirnov test. For comparisons between 2 groups, a 2-tailed unpaired Student’s *t* test (α = 0.05) was applied for normally distributed data (Figure 1F, Figure 2, D, F, J, and L, Figure 4H, Figure 5, J, M, and N, Supplemental Figure 1, B, C, E, and G, Supplemental Figure 2, C and H, Supplemental Figure 3, E, F, H–K, M, and O, Supplemental Figure 4, O and P, Supplemental Figure 5, T–V, and Supplemental Figure 6, B–E, G, H, and K–S). The Mann-Whitney *U* test was used as a nonparametric test (Figure 1D, Figure 2, B and H, Figure 3E, Figure 5, A and L, Supplemental Figure 1, D and H, Supplemental Figure 2E, Supplemental Figure 3, G and L, and Supplemental Figure 5H). Figure 1, K and N, and Supplemental Figure 1O were analyzed using the nested *t* test. When comparing more than 2 groups on a single variable, 1-way ANOVA followed by Tukey’s multiple-comparison test was employed as a parametric test (Figure 1B). In cases where comparisons involved 2 independent variables in multiple groups, 2-way ANOVA was used, followed by either Šidák’s (Figure 1, H, I, K, and L, Figure 3B, Figure 4E, Figure 5, B, H, and I, Supplemental Figure 1, M and N, and Supplemental Figure 2, F and G) or Tukey’s multiple-comparison tests (Figure 3, C, F–K, Figure 4, F, I, J, L, and M, Figure 5F, Supplemental Figure 3, C and R, Supplemental Figure 4, C–I, K, L, and N, Supplemental Figure 5, A–D, F, I–L, Q, and R, and Supplemental Figure 6, I, and J) as a parametric test and the Kruskal-Wallis test with Dunn’s multiple-comparison test as a nonparametric test (Supplemental Figure 3, D and U, and Supplemental Figure 5, O, P, and S). A *P* value of less than 0.05 was considered statistically significant.

### Study approval.

All animal experiments were conducted in compliance with the German Animal Welfare Act and the European Directive 2010/63/EU on the protection of animals used in scientific research and were approved by the local ethics committee for animal experiments (Regierungspräsidium Tübingen).

### Data availability.

All numerical data underlying the graphs are provided in the Supporting Data Values file. Any additional data generated or analyzed during this study that are not included in this article or its supplemental materials are available from the corresponding author upon reasonable request.

## Author contributions

RL initiated the study. JY and RL designed experiments. JY, SS, LZ, LM, SM, and DS performed experiments. JY, SS, MCS, AR, LM, DS, and RL analyzed data. MCS, AR, LM, SM, DS, LB, HB, and NW contributed resources and protocols. JY, AR, MCS, HB, LM, OB, and RL contributed to discussions. JY and RL wrote the manuscript. SS, LZ, SM, DS, LB, LM, HB, MCS, AR, NW, and OB edited the manuscript. RL obtained funding and supervised the project and the personnel. All authors approved the content and submission of the paper.

## Conflict of interest

The authors have declared that no conflict of interest exists.

## Funding support

- Deutsche Forschungsgemeinschaft (DFG) individual grant LU 1490/8-1 and LU 1490/13-1 (to RL).
- Chinese Scholarship Council grants 202008110200 and 202308330048 (to JY and LZ).
- DFG grant 335549539: “cGMP: From Bedside to Bench” (to MCS, LB, DS, and RL).
- Austrian Science Fund (Erwin Schrödinger Program) 10.55776/J4457 (to HB).

## Supplementary Material

Supplemental data

Unedited blot and gel images

Supporting data values

## Version 1 03/17/2026

In-Press Preview

## Version 2 07/08/2026

Electronic publication

## References

[1] Flora GD, Nayak MK (2019). A brief review of cardiovascular diseases, associated risk factors and current treatment regimes. Curr Pharm Des.

[2] Reed GW (2017). Acute myocardial infarction. Lancet.

[3] Ozaki Y (2022). CVIT expert consensus document on primary percutaneous coronary intervention (PCI) for acute myocardial infarction (AMI) update 2022. Cardiovasc Interv Ther.

[4] Jenca D (2021). Heart failure after myocardial infarction: incidence and predictors. ESC Heart Fail.

[5] Velagaleti RS (2008). Long-term trends in the incidence of heart failure after myocardial infarction. Circulation.

[6] Camelliti P (2005). Structural and functional characterisation of cardiac fibroblasts. Cardiovasc Res.

[7] Porter KE, Turner NA (2009). Cardiac fibroblasts: at the heart of myocardial remodeling. Pharmacol Ther.

[8] Shinde AV, Frangogiannis NG (2014). Fibroblasts in myocardial infarction: a role in inflammation and repair. J Mol Cell Cardiol.

[9] Humeres C, Frangogiannis NG (2019). Fibroblasts in the infarcted, remodeling, and failing heart. JACC Basic Transl Sci.

[10] Fu X (2018). Specialized fibroblast differentiated states underlie scar formation in the infarcted mouse heart. J Clin Invest.

[11] Travers JG (2016). Cardiac fibrosis: the fibroblast awakens. Circ Res.

[12] Tallquist MD (2020). Cardiac fibroblast diversity. Annu Rev Physiol.

[13] Gehrmann J (2001). Electrophysiological characterization of murine myocardial ischemia and infarction. Basic Res Cardiol.

[14] Snider P (2009). Origin of cardiac fibroblasts and the role of periostin. Circ Res.

[15] Kaur H (2016). Targeted ablation of periostin-expressing activated fibroblasts prevents adverse cardiac remodeling in mice. Circ Res.

[16] Van den Borne SW (2010). Myocardial remodeling after infarction: the role of myofibroblasts. Nat Rev Cardiol.

[17] Talman V, Ruskoaho H (2016). Cardiac fibrosis in myocardial infarction-from repair and remodeling to regeneration. Cell Tissue Res.

[18] Desmoulière A (1995). Apoptosis mediates the decrease in cellularity during the transition between granulation tissue and scar. Am J Pathol.

[19] McDonagh TA (2023). 2023 focused update of the 2021 ESC guidelines for the diagnosis and treatment of acute and chronic heart failure. Eur Heart J.

[20] Morth JP (2007). Crystal structure of the sodium-potassium pump. Nature.

[21] Grandi E (2017). Potassium channels in the heart: structure, function and regulation. J Physiol.

[22] Benamer N (2009). Molecular and functional characterization of a new potassium conductance in mouse ventricular fibroblasts. J Mol Cell Cardiol.

[23] Benamer N (2013). Fibroblast KATP currents modulate myocyte electrophysiology in infarcted hearts. Am J Physiol Heart Circ Physiol.

[24] Wu CT (2014). Disease and region-related cardiac fibroblast potassium current variations and potential functional significance. Cardiovasc Res.

[25] She G (2019). K_Ca_3.1 channels promote cardiac fibrosis through mediating inflammation and differentiation of monocytes into myofibroblasts in angiotensin II -treated rats. J Am Heart Assoc.

[26] Abraham DM (2018). The two-pore domain potassium channel TREK-1 mediates cardiac fibrosis and diastolic dysfunction. J Clin Invest.

[27] Kameyama M (1984). Intracellular Na+ activates a K+ channel in mammalian cardiac cells. Nature.

[28] Kaczmarek LK (2013). Slack, slick and sodium-activated potassium channels. ISRN Neurosci.

[29] Wojtovich AP (2016). Cardiac Slo2.1 is required for volatile anesthetic stimulation of K+ transport and anesthetic preconditioning. Anesthesiology.

[30] Smith CO Cardiac metabolic effects of K_Na_1.2 channel deletion and evidence for its mitochondrial localization. FASEB J.

[31] Tabula Muris Consortium (2018). Single-cell transcriptomics of 20 mouse organs creates a Tabula Muris. Nature.

[32] Qian L (2012). In vivo reprogramming of murine cardiac fibroblasts into induced cardiomyocytes. Nature.

[33] Martinez-Espinosa PL (2015). Knockout of Slo2.2 enhances itch, abolishes KNa current, and increases action potential firing frequency in DRG neurons. Elife.

[34] Santos MC (2025). Angiotensin II-induced cardiac fibrosis and dysfunction are exacerbated by deletion of cGKI in periostin+ myofibroblasts. Clin Sci (Lond).

[35] Jeevaratnam K (2018). Cardiac potassium channels: physiological insights for targeted therapy. J Cardiovasc Pharmacol Ther.

[36] Schmitt N (2014). Cardiac potassium channel subtypes: new roles in repolarization and arrhythmia. Physiol Rev.

[37] Tamargo J (2004). Pharmacology of cardiac potassium channels. Cardiovasc Res.

[38] Bischof H (2017). Novel genetically encoded fluorescent probes enable real-time detection of potassium in vitro and in vivo. Nat Commun.

[39] Skrabak D (2023). Slack K^+^ channels limit kainic acid-induced seizure severity in mice by modulating neuronal excitability and firing. Commun Biol.

[40] Roslan A (2024). Slack K+ channels confer protection against myocardial ischaemia/reperfusion injury. Cardiovasc Res.

[41] Gross D (2022). IK_Ca_ channels control breast cancer metabolism including AMPK-driven autophagy. Cell Death Dis.

[42] Bischof H (2024). mitoBK_Ca_ is functionally expressed in murine and human breast cancer cells and potentially contributes to metabolic reprogramming. Elife.

[43] Tsuchida K (2021). Monensin-induced increase in intracellular Na+ induces changes in Na+ and Ca2+ currents and regulates Na+-K+ and Na+-Ca2+ transport in cardiomyocytes. Pharmacology.

[44] Dai L (2010). Activation of Slo2.1 channels by niflumic acid. J Gen Physiol.

[45] Garg P, Sanguinetti MC (2012). Structure-activity relationship of fenamates as Slo2.1 channel activators. Mol Pharmacol.

[46] Suzuki T (2020). TMEM16A Ca^2+^-activated cl^-^ channel regulates the proliferation and migration of brain capillary endothelial cells. Mol Pharmacol.

[47] Bradley E (2014). Pharmacological characterization of TMEM16A currents. Channels (Austin).

[48] Mitrokhin V (2023). Transcriptomic profile of the mechanosensitive ion channelome in human cardiac fibroblasts. Exp Biol Med (Maywood).

[49] El Chemaly A (2014). ANO1 contributes to angiotensin-II-activated Ca2+-dependent Cl- current in human atrial fibroblasts. J Mol Cell Cardiol.

[50] Catacuzzeno L, Franciolini F (2018). Role of KCa3.1 channels in modulating Ca^2+^ oscillations during glioblastoma cell migration and invasion. Int J Mol Sci.

[51] Pham T (2023). BK channels sustain neuronal Ca^2+^ oscillations to support hippocampal long-term potentiation and memory formation. Cell Mol Life Sci.

[52] Smith CO (2017). The Slo(w) path to identifying the mitochondrial channels responsible for ischemic protection. Biochem J.

[53] Biton B (2012). The antipsychotic drug loxapine is an opener of the sodium-activated potassium channel slack (Slo2.2). J Pharmacol Exp Ther.

[54] Ross GR (2017). Enhanced store-operated Ca^2+^ influx and ORAI1 expression in ventricular fibroblasts from human failing heart. Biol Open.

[55] Chen JB (2010). Multiple Ca2+ signaling pathways regulate intracellular Ca2+ activity in human cardiac fibroblasts. J Cell Physiol.

[56] Chen PH (2021). Lithium reduces migration and collagen synthesis activity in human cardiac fibroblasts by inhibiting store-operated Ca^2+^ entry. Int J Mol Sci.

[57] García-Casas P (2018). Inhibition of sarco-endoplasmic reticulum Ca^2+^ ATPase extends the lifespan in C. elegans worms. Front Pharmacol.

[58] Bird GS (2008). Methods for studying store-operated calcium entry. Methods.

[59] Nelson HA (2018). Interplay between ER Ca^2+^ binding proteins, STIM1 and STIM2, is required for store-operated Ca^2+^ entry. Int J Mol Sci.

[60] Rubaiy HN (2023). ORAI calcium channels: regulation, function, pharmacology, and therapeutic targets. Pharmaceuticals (Basel).

[61] Yu C (2022). Orai3 mediates Orai channel remodelling to activate fibroblast in pulmonary fibrosis. J Cell Mol Med.

[62] Kumar R (2018). Mitochondrial uncoupling reveals a novel therapeutic opportunity for p53-defective cancers. Nat Commun.

[63] Xie Y (2012). Immunohistochemical detection of differentially localized up-regulation of lysyl oxidase and down-regulation of matrix metalloproteinase-1 in rhesus monkey model of chronic myocardial infarction. Exp Biol Med (Maywood).

[64] Korte T (2002). In-vivo electrophysiological study in mice with chronic anterior myocardial infarction. J Interv Card Electrophysiol.

[65] Suthahar N (2017). From inflammation to fibrosis-molecular and cellular mechanisms of myocardial tissue remodelling and perspectives on differential treatment opportunities. Curr Heart Fail Rep.

[66] Kumar V, Bansal SS (2025). Immunological regulation of fibrosis during heart failure: it takes two to tango. Biomolecules.

[67] Choudhary M, Malek G (2023). CD68: potential contributor to inflammation and RPE cell dystrophy. Adv Exp Med Biol.

[68] Liu D (2024). Key links in the physiological regulation of the immune system and disease induction: T cell receptor -CD3 complex. Biochem Pharmacol.

[69] Zhang J (2023). CXCL16 promotes Ly6Chigh monocyte infiltration and impairs heart function after acute myocardial infarction. J Immunol.

[70] Butwell NB (1993). Effect of lidocaine on contracture, intracellular sodium, and pH in ischemic rat hearts. Am J Physiol.

[71] Singh Y (2024). Repurposing of niclosamide, an anthelmintic, by targeting ERK/MAPK signaling pathway in the experimental paradigm of autism spectrum disorders. Eur J Pharmacol.

[72] Hu N (2021). Chemical mitochondrial uncouplers share common inhibitory effect on NLRP3 inflammasome activation through inhibiting NFκB nuclear translocation. Toxicol Appl Pharmacol.

[73] (1986). A model for receptor-regulated calcium entry. Cell Calcium.

[74] Mercer JC (2006). Large store-operated calcium selective currents due to co-expression of Orai1 or Orai2 with the intracellular calcium sensor, Stim1. J Biol Chem.

[75] Frischauf I (2011). Cooperativeness of Orai cytosolic domains tunes subtype-specific gating. J Biol Chem.

[76] Inayama M (2015). Orai1-Orai2 complex is involved in store-operated calcium entry in chondrocyte cell lines. Cell Calcium.

[77] Wang L (2022). ORAI3 is dispensable for store-operated Ca2+ entry and immune responses by lymphocytes and macrophages. J Gen Physiol.

[78] Zhang B (2016). Store-operated Ca^2+^ entry (SOCE) contributes to angiotensin II-induced cardiac fibrosis in cardiac fibroblasts. J Pharmacol Sci.

[79] Prakriya M, Lewis RS (2015). Store-operated calcium channels. Physiol Rev.

[80] Ferreira JJ (2021). SLO2.1/NALCN a sodium signaling complex that regulates uterine activity. iScience.

[81] Engel P (2024). Slick potassium channels limit TRPM3-mediated activation of sensory neurons. Front Pharmacol.

[82] Javed A (2023). The relationship between myocardial infarction and estrogen use: a literature review. Cureus.

