# Supplementary material for: Slick K+ channels contribute to cardiac remodeling, fibrosis, and dysfunction in postinfarction hearts
Source: JCI Insight. 2026 Mar 17;11(13):e195805. doi: 10.1172/jci.insight.195805 (PMC13460849; doi:10.1172/jci.insight.195805)
Supplement: Supplementary file 1 [file jciinsight-11-195805-s137.pdf]

Research Article

# Slick $K^+$ channels contribute to cardiac remodeling, fibrosis and dysfunction in post-infarction hearts

Jiaqi Yang[1], Lin Zhu[1], David Spähn[1], Melanie Cruz Santos[1], Sophia Schanz[1], Selina Maier[1], Lena Birkenfeld[1], Helmut Bischof[1], Anna Roslan[1], Nina Wettschureck[2], Oliver Borst[3], Lucas Matt[1], Robert Lukowski[1, §]

[1]Department of Pharmacology, Toxicology and Clinical Pharmacy, Institute of Pharmacy, University of Tübingen, Tübingen, Germany

[2]Max-Planck-Institute for Heart and Lung Research, Department of Pharmacology, Bad Nauheim, Germany

[3]DFG Heisenberg Group Thrombocardiology; Department of Cardiology, Angiology and Cardiovascular Medicine, University of Tübingen, Tübingen, Germany

[§]Corresponding author:

Robert Lukowski
Experimental Pharmacology
Department of Pharmacology, Toxicology and Clinical Pharmacy
Institute of Pharmacy
University of Tübingen
Tel. +49 7071 29 74550
Fax +49 7071 29 2476
E-mail: robert.lukowski@uni-tuebingen.de

- Supplemental methods and materials
- Supplemental figures and Legends
- Supplemental table
- Supplemental references

## Supplemental methods and materials

### Open-chest I/R surgery and determination of myocardial cell death

*Slick*$^{-/-}$ mice and their control littermates (*Slick*$^{+/+}$) were subjected to an open-chest I/R surgery model, which allows assessment of myocardial cell death following previously described protocols (1, 2). Briefly, mice were anesthetized by pentobarbital sodium (70 mg/kg, i.p.) diluted in 0.9% saline, then placed on a heating plate and connected via an anal temperature detector to maintain body temperature at 37°C. Intubation and oxygen ($O_2$) ventilation were performed using a volume-controlled small animal ventilator (Hugo Sachs Electronic, type 845). A thoracotomy was made between the third and fifth ribs to expose the heart. LCA was occluded for 30 min using a 7-0 polyimide suture with a plastic tube and then reopened for 2 h ($I_{30min}/R_{2h}$) (2). Thereafter, the suture was firmly tied before 1% Evans Blue solution (AppliChem, #A4388) in PBS (Gibco, #14190-094) was injected through the thoracic aorta to stain the area not at risk (ANAR), i.e., the myocardium proximal to the ligation. The heart was then quickly weighed, frozen at -20°C for 20 min before sectioning into 1 mm slices. Sections obtained were incubated in 1% TTC (Sigma-Aldrich, #93140) solution at 37°C for 30 min to distinguish viable myocardium (stained red) from infarcted regions. Following incubation in 10% formaldehyde solution (Sigma-Aldrich, #F8775), heart slices were photographed with a camera (Canon EOS M5) in the presence of a scale bar to convert pixels into centimeters, and the AAR and infarcted area were determined using AxioVision software (Zeiss). Infarction was expressed as a percentage of the AAR,

while AAR was calculated in a similar manner from the total area of the heart slice.

**Closed-chest I/R surgery**

Cardiac remodeling and imaging were evaluated in response to a chronic I/R surgery protocol (1). Therefore, experimental mice were anesthetized using a combination of midazolam (5 mg/kg), medetomidine (500 µg/kg), and fentanyl (50 µg/kg). Body temperature was maintained at 37°C by a controlled heating plate. Upon intubation, animals were ventilated with 1.0-2.0 Vol% isoflurane (CP Pharma, #400806.00.00)/$O_2$ to maintain anesthetic depth. A minimally invasive thoracotomy was performed between the third and fourth ribs to gain access to the LCA, which was closed with a 7-0 polypropylene suture (ETHICON, #EH7813E) for 45 min before being reopened to allow reperfusion. After closing the chest, anesthesia was terminated using a combination of flumazenil (0.5 mg/kg) and atipamezole (2.5 mg/kg). For pain management during and after the operation, mice received buprenorphine (0.1 mg/kg) s.c. and metamizole (1.25 mg/ml in drinking water). Further analyses were carried out after either 1 or 4 weeks ($I_{45min}/R_{1week}$ or $I_{45min}/R_{4weeks}$), depending on the purpose of the experiment. For the sham operation, mice underwent the same procedure as described above, except for the LCA ligation. Instead, a 7-0 polypropylene suture was placed underneath the vessel without tightening it. After 45 min to mimic the period of ischemia, the suture was removed, and the chest was closed following an identical procedure as described for the chronic I/R injury model.

**ECG measurement**

ECG was performed at baseline and $I_{45min}/R_{1week}$-exposed mice derived from global Slick KO and male littermate controls by employing a non-invasive small animal physiological monitoring system (Harvard Apparatus). To minimize the influence of circadian rhythm on HR, all measurements were conducted between 8:00 and 9:00 am (3). Mice were anesthetized with 1-2% isoflurane/$O_2$ and positioned prone on the ECG monitoring platform. Limb electrodes were attached using conductive gel (PARKER, #TB-060-0244L), and body temperature was maintained at 37 °C via a rectal thermoprobe-controlled heating system. Once a stable anesthetic depth was achieved, as indicated by a consistent respiratory rate and HR, ECG signals were recorded continuously for 5 min at a sampling frequency of 1 kHz (4). Surface ECGs were acquired in lead II configuration, as this lead provides the clearest representation of the QRS complex, T wave, and ST segment, which are key indicators of ventricular electrophysiology and particularly susceptible to I/R-induced alterations (5). Data were analyzed using LabChart 8.0 (ADInstruments) with the ECG analysis module, applying the mouse-specific preset parameters. Quantitative measurements included wave amplitudes (P, Q, R, S, T), intervals (PR, QRS, QT, JT), and ST segment height. Corrected QT intervals (QTc) were calculated using the formula described by Mitchell et al., which is validated for murine ECG analysis (6).

**Echocardiographic measurement**

Non-invasive echocardiography was performed using a 30 MHz transducer in conjunction with the Vevo® 2100 imaging system for cardiovascular applications

(FUJIFILM Visual Sonics). Mice that underwent sham or $I_{45min}/R_{4weeks}$ operations were subjected to echocardiographic examination as previously described (1, 2). Prior to the measurements of cardiac dimensions and functions, the left thoracic region was depilated in mice anesthetized with 1.0-2.0 Vol% isoflurane/$O_2$. Next, serial M-mode and B-mode images were captured in the PLAX view, recording 3 cardiac cycles in each measurement and in total 3 times per mouse. Ultrasound data were then analyzed using the Vevo® LAB ultrasound analysis software (FUJIFILM Visual Sonics). B-mode images were analyzed using a speckle-tracking algorithm. The operator manually placed 10-15 tracking points along the LV to accurately delineate the endocardial and epicardial borders (1). Cardiac functional parameters, including LVEF, CO, and SV, were calculated automatically by the software. Myocardial velocity and deformation were assessed using strain analysis in both longitudinal and radial directions. Strain parameters were evaluated separately for the endocardial and epicardial layers to distinguish regional myocardial function.

**Sirius Red fibrosis staining**

Hearts subjected to chronic $I_{45min}/R_{4weeks}$ or sham operations were isolated and perfused with ice-cold PBS using a Langendorff perfusion system, followed by fixation with 4% Paraformaldehyde (PFA; Carl Roth, #0335.2) at 4°C for 4 h. After fixation, hearts were washed with PBS and stepwise dehydrated using 5%, 10%, and 20% sucrose solutions, respectively, then embedded in NEG medium (Epredia, #6502), and frozen at -80°C. Using a microtome (Thermo Scientific, HM 560), hearts were cut into

8-10 sets separated by a gap of 250 μm. For each set, 10 μm-thick slices were collected for the whole consecutive 100 μm. This sampling covered the entire myocardium distal to the LCA ligation. After cryosectioning, samples were stored at -20°C for subsequent staining.

To highlight connective fibers by Sirius Red staining, cryosections were thawed at RT, fixed in Bouin's solution (Sigma-Aldrich, #HT101126) for 24 h. Subsequently, they were stained with Sirius Red solution containing 0.1% direct red 80 (Sigma-Aldrich, #365548) in aqueous saturated picric acid (Sigma-Aldrich, #36011) for 1 h. After staining, sections were washed and acidified using 0.01 M HCl to remove unbound dye, followed by dehydration through a graded ethanol series (50%, 75%, 95%, and 100%) and sample clearing by using xylene. Finally, specimens were mounted using DPX (Sigma-Aldrich, #1.00579). After drying overnight, heart sections were scanned using a PANNORAMIC™ Digital Slide Scanner, and the fibrosis area in relation to the total area of the heart sections was quantified using ImageJ software.

To differentiate between mature and immature collagen fibers, Sirius Red–stained heart sections were imaged under polarized light using a Nikon Ni-U microscope equipped with a 20x Plan Fluor objective and NIS-Elements D software (v5.21.00, 64-bit). Whole-heart slice images were acquired as merged composites using a 1-second exposure time. Image analysis was performed with ImageJ software. Mature collagen fibers (predominantly type I) were quantified based on their red birefringence, whereas immature fibers (mainly type III) were identified from the green birefringence. Col1/Col3

ratio was calculated as an indicator of collagen network maturity and tissue stiffness (7).

**Detection of cell death by TUNEL staining**

To assess the apoptotic CM death, heart cryosections from CTR and mKO mice following $I_{45min}/R_{4weeks}$ operation were employed. Therefore, cryosections were thawed at RT for 30 min and then fixed with 4% PFA. After washing, sections were permeabilized with 0.3% Triton X-100 (Carl Roth, #9002-93-1) in PBS for 30 min at RT. To reduce auto-fluorescence, with CMs as the primary source, sections were treated twice with a chemical cocktail containing 3% hydrogen peroxide ($H_2O_2$) and 50 mM sodium hydroxide (NaOH) for 45 min each under LED light, as previously described (2). Following additional washing steps, a blocking buffer consisting of 2% glycerol, 50 mM $NH_4Cl$, 10% normal goat serum (NGS; Biozol, #ENG-9010-10), 2% BSA (Carl Roth, #0163.4), and 0.3% Triton X-100 in PBS was applied for 1 h at RT to prevent non-specific binding. Sections were then incubated overnight at 4°C with a primary antibody targeting Troponin I (Cell Signaling Technology, #4002S, diluted 1:100 in blocking buffer). The next day, the primary antibody solution was removed, and sections were washed with PBS containing 0.01% Triton X-100 and 1.5% NGS. Alexa 488-conjugated secondary antibody, diluted 1:2000 in blocking buffer, was applied for 2 h at RT.

For TUNEL staining, a positive control slide was treated with 10,000 units of recombinant DNase I (Roche, #04716728001) at RT for 15 min to expose DNA fragments. Labeling solutions with and without enzyme were applied (50 µl per heart

slice) for 1 h at 37°C to serve as sample and NC, respectively. After thorough washing with PBS, sections were stained and mounted with Hoechst 33342 (Sigma-Aldrich, #94403) in PermaFluor (Epredia, #TA-030-FM). Images were captured using a ZEISS LSM 980 confocal microscope. Apoptotic CMs were identified by co-staining of Hoechst 33342, TUNEL, and Troponin I. Analysis was performed using ImageJ software. The number of TUNEL-positive cells was expressed as a percentage of all Troponin I and Hoechst-positive cells within an area of interest.

**Quantification of CSA**

To evaluate the hypertrophic response of CMs following $I_{45min}/R_{4weeks}$ injury, we assessed CM enlargement by measuring CSA. Alexa Fluor 555-conjugated WGA (Thermo Scientific, #W32464), which specifically binds to glycoproteins of cell membranes, was applied to heart cryosections for labeling CM sarcolemma. In brief, slides were thawed at RT for 30 min and treated with a chemical cocktail under LED light for 90 min to reduce tissue autofluorescence. Subsequently, sections were incubated in blocking buffer for 1 h at RT, as described above. Heart cryosections were stained with WGA (1:1000 dilution in PBS) for 30 min in the dark at RT. After brief washing steps, slides were mounted using PermaFluor mounting medium (Epredia, #TA-030-FM) containing Hoechst 33342 for nuclear staining. Two adjacent heart sections per heart were stained and imaged using a ZEISS LSM 980 confocal microscope. Only non-deformed endocardial CMs were included in the analysis. A minimum of 80 CMs per heart were analyzed using Zeiss Blue Edition software, and

the average CSA was calculated to quantify hypertrophic remodeling in response to MI or sham.

**IF staining of immune cells**

Cryosections of $I_{45min}/R_{4weeks}$ hearts were permeabilized with 0.3% Triton X-100 in PBS, followed by an autofluorescence reduction step as described above. After blocking, sections were incubated overnight at 4°C with primary antibodies against CD3 (Abcam, ab135372) to label T cells, and CD68 (Abcam, #ab283654) to label macrophages (8, 9). The following day, slides were washed and incubated with Alexa Fluor 555-conjugated goat anti-rabbit secondary antibody (Thermo Scientific, #A-21428). Fluorescent images were acquired using a ZEISS LSM 980 confocal microscope. The number and frequency of $CD3^+$ T cells and $CD68^+$ macrophages were quantified in both fibrotic and non-fibrotic regions of the heart tissue using ImageJ software.

**Immunohistochemical detection of Slick channels by AP staining**

Heart cryosections designated for immunohistochemical Slick detection were thawed at RT for 30 min prior to tissue permeabilization, which was achieved using 0.3% Triton X-100 in PBS. To prevent non-specific binding, slides were blocked with 10% NGS for 1 h at RT. Endogenous biotin was reduced by incubation with avidin solution (VectorLabs, #SP-2001) for 15 min and then biotin solution (VectorLabs, #SP-2001) for another 15 min. Following washes with PBS, slides were incubated overnight at 4°C with primary anti-Slick antibody (1:250, Neuromab, #75-055). The next day, primary antibody was removed, and the heart slices were washed with PBS containing

1.5% NGS. A biotinylated secondary antibody (VectorLabs, #BA-9200) was then applied for 1 h at RT. Subsequently, slides were incubated with ABC-AP reagent (VectorLabs, #AK-5000) for 30 min at RT in the dark to visualize antigen-antibody complexes. For the final step, slides were incubated with AP-substrate solution, prepared by mixing 5 ml of 100 mM Tris-HCl (pH 8.2) with 200 µl each of reagent 1, reagent 2, reagent 3, and levamisole (VectorLabs, #SK-5300), an inhibitor of endogenous AP activity. The NC specimen, which was prepared in parallel by exclusion of the primary antibody, was used to determine the optimal incubation time for the AP substrate solution. Staining was terminated once the NC slides developed a blue color. Finally, sections were mounted with Aquatex covering medium (VWR, #363123S). PANNORAMIC™ Digital Slide Scanner was used for scanning.

**Overexpression of recombinant Slick channels in HEK293 cells**

An agar stab containing *E. coli* harboring a Slick (i.e., murine Slo2.1 sequence) encoding plasmid (Addgene, #197317) was streaked onto a lysogeny broth (LB) agar plate supplemented with 100 µg/ml ampicillin (Roth, #K029.2). After overnight incubation, a single colony was selected for further expansion in 500 ml of LB medium containing 100 µg/ml ampicillin. *pcDNA3.1-Slick* was extracted using the NucleoBond Xtra Maxi kit (Macherey-Nagel, #740414.10), following the manufacturer's protocol for low-copy plasmids. The identity of the extracted pcDNA3.1-Slick vector was confirmed by using the following primers for sequencing: *pcDNA3.1*$^{for}$ 5'-CTC TGG CTA ACT AGA GAA C-3', *pcDNA3.1*$^{rev}$ 5'-CCA GGG TCA AGG AAG GCA C-3'.

HEK293 cells were cultured in Dulbecco's Modified Eagle Medium (DMEM; Gibco, #42430-025) supplemented with 10% FBS (Gibco, # A5256701) and 1% Penicillin-Streptomycin solution (PenStrep; Gibco, #15140-122). HEK293 cells were passaged using 0.5% Trypsin-EDTA (Gibco, #15400-054), and subcultures 8 to 15 passages were used for subsequent experiments. For transfection, cells were seeded in 10 cm culture dishes and cultured until they reached 70–80% confluency. According to the manufacturer's recommendations, culture medium was refreshed 30 min prior to transfection using PolyJet transfection reagent (SignaGen, #SL100688). To facilitate visualization of transfection efficiency, cells were co-transfected with *pcDNA3.1-Slick* and *pcDNA3.1-mRuby3* plasmids, whereby the latter encodes RFP. NC cells were transfected with *pcDNA3.1-mRuby3* alone unless otherwise specified.

**Electrophysiology**

To characterize Slick-mediated currents, we performed whole-cell patch-clamp recordings on freshly isolated RCF, cultured CMF, and $CMF^{I/R}$ from both untreated and $I_{45min}/R_{1week}$-injured hearts. To this end, 12 mm diameter coverslips (VWR, #ECN631-1577) seeded with 400,000 cells were placed in a submerged-type recording chamber (Warner Instruments, #RC-26G) connected to a perfusion system (Gilson, minipuls2) supplying extracellular buffer (in mM: 2 $CaCl_2$, 1 $MgCl_2$, 138 NaCl, 5 KCl, 10 HEPES, and 10 glucose with the pH adjusted to 7.6). Cells were visualized using a Nikon Ts2R, equipped with a Nikon S Plan Fluor 40x/0.6 objective with EMBOSS contrast (Nikon Instruments Inc.) and a DFK 33Ux174 camera (The Imaging Source). Micropipettes

(5-8 MΩ) pulled from borosilicate glass (Science Products GmbH, #BM150-10P) using a P-1000 micropipette puller (Sutter Instruments) and polished by an MF-830 microforge (Narishige International Ltd.) were filled with intracellular buffer (in mM: 130 K-Gluconate, 10 KCl, 5 $MgCl_2$, 0.6 EGTA, 5 HEPES, 0.1 $CaCl_2$, 2 $Na_2$-ATP, 0.2 $Na_2$-GTP). Whole-cell recordings were sampled at 5 kHz using an EPC10 amplifier and PatchMaster software (HEKA Elektronik GmbH). Whole-cell capacitance and series resistance were compensated. To identify Slick-mediated outward currents in a physiologically relevant range, RCF and CMF were held at -70 mV and subjected to 300 ms voltage steps from -80 mV to +100 mV in 20 mV increments, both before and 5 min after the perfusion of $K_{Na}$ openers (e.g., 1 mM NFA (Sigma-Aldrich, #N0630-10)), or the $Na^+$ ionophore monensin (10 µM), the latter promotes Slick channel activation *via* its $Na^+$-sensitive gating mechanism (10-12). Amplitudes of persistent (steady state) currents were measured as the mean amplitude within the last 30 ms of the pulse using FitMaster software (HEKA Elektronik GmbH). Representative currents were exported using Nest-O-Patch software (Glorfin).

**FRET-based $[K^+]_{cyto}$ imaging**

To visualize the $[K^+]_{cyto}$ dynamics in live RCF and CMF, viral transduction was performed using a replication-incompetent gutless adenovirus (AV)-based vector with an MOI of 50 for 48 h. The virus was encoded by the cytosolic-targeted GEPII 1.0 NES lc-LysM $[K^+]_{cyto}$-indicator under control of a CMV promoter (VectorBuilder). Culture medium was replaced with pre-warmed cell equilibration buffer 30 min before imaging

(in mM: 2 $CaCl_2$, 10 D-glucose, 10 HEPES, 5 KCl, 2 L-glutamine, 0.44 $KH_2PO_4$, 1 $MgCl_2$, 135 NaCl, 2.6 $NaHCO_3$, 0.34 $Na_2HPO_4$, 1x MEM amino acids, 1x MEM vitamins, 1x PenStrep) at RT for cell pre-equilibration. Afterward, cells were perfused using a gravity-based perfusion system (NGFI GmbH) with imaging buffer containing 2 $CaCl_2$, 1 $MgCl_2$, 138 NaCl, 5 KCl, 10 HEPES, and 10 glucose in mM, with the pH adjusted to 7.4. Live-cell imaging was performed using a Zeiss microscope (Axiovert 200m) equipped with a Plan-Neofluar 40x/1.30 oil immersion objective, an Optosplit II emission image splitter (Cairn Research Ltd.) with a T505lpxr filter (AHF Analysentechnik) and supplemented with the High-Power LED Light Engine (Omicron Laserage, #2200114 LED-Hub®). Images were captured using a pco.panda 4.2 bi sCMOS camera (pco.) with VisiView software (Visitron Systems). Coverslips containing transduced cells were placed in a PC30 perfusion chamber (NGFI GmbH). Cells were measured in imaging buffer for 5 min to establish a baseline. Next, monensin sodium salt (10 µM; Sigma-Aldrich, #46468) was added for an additional 15 min to cause a massive influx of $Na^+$ for opening $Na^+$-activated Slick channels (12). A setup-specific threshold intensity was defined to ensure sufficient transduction efficiency for statistical analysis. Nearly all cells survived the measurements and were included in the final analysis. Intensity of FRET and cyan fluorescent protein (CFP) was recorded in real-time, and the FRET / CFP ratio was calculated following background subtraction and bleaching correction using Prism 10.0.

For FRET-based $[K^+]_{cyto}$ measurements in HEK293 cells overexpressing Slick, cells were co-transfected with pcDNA3.1-Slick and pcDNA3.1-GEPII 1.0 NES lc-LysM 48 h

before the experiment. NC cells were transfected with pcDNA3.1-GEPII 1.0 only. During the measurement, niclosamide (10 µM; Sigma-Aldrich, #N3510-50), a $K_{Na}$ channel opener (11, 13), was perfused for 15 min, and Slick activation was calculated following a 5 min baseline stabilization period.

**$[Ca^{2+}]_i$ measurement**

RCF and CMF were loaded with 2 µM Fura-2 fluorescent dye (Sigma-Aldrich, #47989) in cell equilibration buffer (as described above) for 30 min in the dark. Cells were then excited using the CoolLED pE-340Fura illumination system (CoolLED). Emissions resulting from excitation at 340 nm and 380 nm, which represent the $Ca^{2+}$-bound and $Ca^{2+}$-unbound forms of the Fura-2 dye, respectively, were captured using the Zeiss setup previously described. To investigate the basal $[Ca^{2+}]_i$ storage, the $Ca^{2+}$ ionophore ionomycin (3 µM) was applied to induce $Ca^{2+}$ leak by transitioning from a 2 mM $Ca^{2+}$ extracellular buffer to a $Ca^{2+}$-free buffer (0 mM $Ca^{2+}$ + 0.1 mM EGTA) after 5 min of baseline measurement (14). Niclosamide (10 µM) was employed to facilitate the opening of the Slick channel in RCF and CMF for 10 min following the baseline measurement. To induce SOCE, $Ca^{2+}$ from the ER was depleted by applying a $Ca^{2+}$-free extracellular buffer together with BHQ (15 µM), an inhibitor of SERCA (15). Subsequently, replacement of the extracellular buffer with 2 mM $Ca^{2+}$ initiated SOCE. This protocol is commonly used to address SOCE in multiple cell types, including RCF and CMF (16-18). Additionally, the Orai inhibitor GSK7975A (10 µM) was employed to block SOCE following the aforementioned protocol (19). The ratio of the intensity at

340 nm / 380 nm after background subtraction and bleaching correction was employed for statistical analysis.

**Cytoplasmic/mitochondrial membrane potential measurement**

$DiBAC_4(3)$ (Thermo Scientific, #B438) was used to measure cytoplasmic membrane potential ($\varphi_m$) in HEK293 cells overexpressing Slick. Cells were preincubated for 30 min in equilibration buffer containing 0.25 µg/ml $DiBAC_4(3)$ before being analyzed using a Zeiss fluorescent microscope (20). Fluorescence excitation was performed at 485 nm, and emission was captured at 525 nm.

To assess $\Delta\varphi_{mito}$ in CMFs, cells were incubated with 200 nM TMRM (Invitrogen, T668) for 20 min in the dark using imaging buffer composed of (in mM): 138 NaCl, 5 KCl, 2 $CaCl_2$, 1 $MgCl_2$, 10 HEPES, and 10 glucose (pH 7.4). Live-cell imaging was performed using a Zeiss fluorescence microscope with excitation at 575 nm and emission collected at wavelengths ≥600 nm (20). After 5 min of baseline recording, cells were perfused for another 5 min with either 10 µM niclosamide or vehicle control. Subsequently, 1.5 µM carbonyl cyanide-p-trifluoromethoxyphenylhydrazone (FCCP; Santa Cruz Biotechnology, #SC-203578) was applied to fully depolarize the mitochondrial membrane. Fluorescence intensities of mitochondria ($F_{mito}$) were measured in regions of interest (ROI) and corrected by the corresponding nuclear fluorescence ($F_{nuc}$) to account for background signal and dye distribution. Data were further normalized to the ratio obtained after complete depolarization with FCCP to allow comparison across cells and treatment conditions.

**OCR assay**

CMFs were seeded at a density of 40,000 cells per well in Seahorse XFe24 FluxPak microplates (Agilent, #02342-100) and cultured for 10 days. One day before the measurement, the sensor cartridge was hydrated with Seahorse XF Calibrant (Agilent, #100840-000) at 37°C under ambient air conditions overnight. At the same time, the Seahorse XFe24 Analyzer (Agilent, #30916332) and the associated Wave Controller software (Agilent) were switched on and left running overnight for system stabilization. On the day of the measurement, cells were washed 3 times with pre-warmed Seahorse assay medium (Agilent, #103575-100) supplemented with 1 mM pyruvate (Gibco, #11360-070), 2 mM GlutaMAX (Gibco, #35050-038), 10 mM glucose (Gibco, #A24940-01), and 1% ITS supplement (Sigma, #I3146). Cells were then equilibrated for min at 37 °C in ambient air. Meanwhile, the hydrated sensor cartridge was placed in the analyzer for a 30 min equilibration period before assembly with the cell culture plate. Immediately before the start of the assay, niclosamide (10 µM) or vehicle (DMSO) was added to the cells. Following a 20 min baseline measurement, the mitochondrial stress test was performed through sequential injections of 2 µM oligomycin (Oligo; Santa Cruz Biotechnology, #SC-203342), 1.5 µM FCCP (Santa Cruz Biotechnology, #SC-203578), and 2.5 µM antimycin A (Santa Cruz Biotechnology, #SC391459) plus 1 µM rotenone (Sigma-Aldrich, #R8875) (Anti/Rot). Each injection cycle included 3 measurement points recorded every 8 min. Data were analyzed using the Wave Analyzer software (Agilent), and OCR values were normalized to total protein content per well, determined by the Bradford assay.

**Grid-plate-based proliferation assay**

$CMF^{I/R}$ was isolated from CTR and mKO mice following $I_{45min}/R_{1week}$ injury. The cells were seeded in Grid-500 chambers (ibidi, #80826-G500) at a density of 40,000 cells per well. After 24 h of culture, the medium was deprived of FBS and ITS for 24 h to synchronize the cell cycle. At 48 h in vitro, the culture medium containing FBS was reintroduced, and images were captured using a bright-field microscope with VisiView software (Visitron Systems) as the starting point (0 h). Subsequent images were taken at the same locations at 24 h, 48 h, and 72 h, respectively. The relative proliferation rate at each time point was calculated relative to the 0 h baseline. Each experiment included 3 technical replicates per mouse heart.

**MTT-based proliferation assay**

CMFs from CTR and mKO hearts following $I_{45min}/R_{1week}$ injury were isolated for an MTT assay. CMFs (2,000 cells per well) were seeded into 96-well plates (SARSTEDT, #83.3924), with 3 technical replicates performed for each genotype at each time point (i.e. 0 h, 24 h, 48 h, 72 h, and 96 h). Serum deprivation was carried out for 24 h in DIV1, followed by reintroduction of 10% FBS at 0 h in DIV2. MTT (Thermo Scientific, #6494) dissolved in DMSO with a concentration of 12 mM was diluted 1:10 in culture media and applied to the wells at the designated time points. Plates were incubated at 37°C for 4 h to enable MTT interaction with viable cell mitochondria. Post-incubation, MTT-containing media were carefully removed, and violet formazan crystals were dissolved in DMSO by incubating at 37°C for 10 min. OD values were measured using a plate

reader (TECAN, Infinite F200 PRO) at 570 nm. The relative proliferation rate was normalized to the baseline (0 h).

**RT-qPCR analysis**

Cardiac cells (including RCF, CMF, $CMF^{I/R}$, and CM) and heart tissue exposed to I/R injury were subjected to RNA extraction using TRI reagent (Sigma Aldrich, #T9424) according to the manufacturer's instructions. DNase I treatment was performed to eliminate genomic DNA contamination (Roche, #04716728001). Total RNA (0.5 µg) was used for cDNA synthesis with 4 µl iScript™ (Bio-Rad, #170-8896), with and without 1 µl reverse transcriptase, to generate experimental (+RT) and control probes (-RT). The synthesized cDNA samples were then diluted 1:10 with DEPC water (Carl Roth, #1143). For RT-qPCR, 3.5 µl of diluted cDNA (or -RT probes) were mixed with forward and reverse primers (100 nmol/l) and 7.5 µl of SSoAdvanced Universal SYBR Green Supermix (Bio-Rad, #1725274). Reactions were performed in triplicate using the CFX Connect Real-Time PCR Detection System (Bio-Rad). Target gene expression levels were normalized to hypoxanthine-guanine phosphoribosyl transferase (*Hprt*) using the $2^{-\Delta Ct}$ method. Primer pairs used for the detection of the respective genes are shown in **STable 1**. For electrophoresis, the RT-qPCR products and corresponding -RT controls were mixed with gel-loading dye (New England Biolabs, #B7025S). The samples were then loaded onto a 2% agarose gel along with a DNA ladder (New England Biolabs, #N3200S) to confirm predicted DNA fragment sizes. Electrophoresis was performed, and the resulting gel was visualized using UVP Gel Studio PLUS, with VisionWorks

software (AnalytikJena) controlling image acquisition and analysis.

**Western blot analysis**

Proteins from either HEK293 cells or murine cardiac cells were extracted using RIPA lysis buffer (Sigma-Aldrich, #52332) supplemented with 1x protease inhibitor cocktail (Roche, #11836170001), 0.8 mM PMSF (Sigma-Aldrich, #52332), and 1x phosphatase inhibitor cocktails II (Sigma-Aldrich, #P5726) and III (Sigma-Aldrich, #P0044). The samples were incubated at 4°C on a shaking platform for 30 min to facilitate extraction and then centrifuged at 12,000 rpm for 30 min to further purify the proteins. Protein concentration was determined using the BCA assay kit (Thermo Scientific, #23225) according to the manufacturer's instructions, with the results read using a scanning device (TECAN, Infinite F200 PRO). Protein samples were then mixed with 1x Laemmli buffer containing 250 mM DTT and heated to 95°C for 15 min before storage. For Slick detection, samples were incubated under milder conditions at 37°C for 30 min. Approximately 50 µg of proteins per sample were loaded onto SDS-PAGE for electrophoresis using a buffer containing 248 Tris-HCl, 1920 glycine in mM, and 1% SDS, adjusted to pH 8.3. After electrophoresis, proteins were transferred to a 0.45 µm PVDF-FL membrane (Sigma-Aldrich, #IPFL00010) using a semi-dry transfer method at 60 mA for 60 min, followed by 90 mA for 15 min for high molecular weight proteins. Transfer buffers were prepared as follows: anode 1 (300 mM Tris-HCl, 20% methanol, pH 10.4), anode 2 (30 mM Tris-HCl, 20% methanol, pH 10.4), and cathode buffer (25 Tris-HCl, 44.2 6-aminocaproic acid in mM, 20% methanol, pH 7.6). Blocking was

performed with 5% milk (Carl Roth, #T145.3) or BSA in 1x Tris-buffered saline with Tween 20 (TBST; 10 Tris-HCl, 140 NaCl in mM, 0.005% Tween-20 purchased from Carl Roth). Membranes were incubated overnight at 4°C with primary antibodies diluted in 1 x TBST containing 1% BSA and 0.05% sodium azide ($NaN_3$). Primary antibodies used were Slick (1:250, Neuromab, #75-055), α-SMA (1:1000, Abcam, #EPR5368), Orai1 (1:500, Abcam, #AB244352), Orai3 (1:500, Abcam, #AB254260). The following day, membranes were washed with 1x TBST and incubated with HRP-conjugated secondary antibodies (Sigma-Aldrich, #18111932 and #18174475), in a 1:5000 dilution with 1x TBST, for 1 h at RT. After washing with 1x TBST, membranes were developed using an HRP substrate solution (Sigma-Aldrich, #232653) and imaged using a scanning system (Gelifesciences, #AI600). If necessary, a strong stripping buffer (Sigma-Aldrich, #2504) was used to remove the primary antibody and secondary antibody from the membrane. The membrane was incubated with 1x stripping buffer diluted in TBST for 15 min at RT. Following the stripping step, the membrane was thoroughly washed, re-blocked under the same conditions as the initial incubation and subsequently probed with a different primary antibody. Band intensities were analyzed using ImageJ software.

## Supplemental Figures and Legends

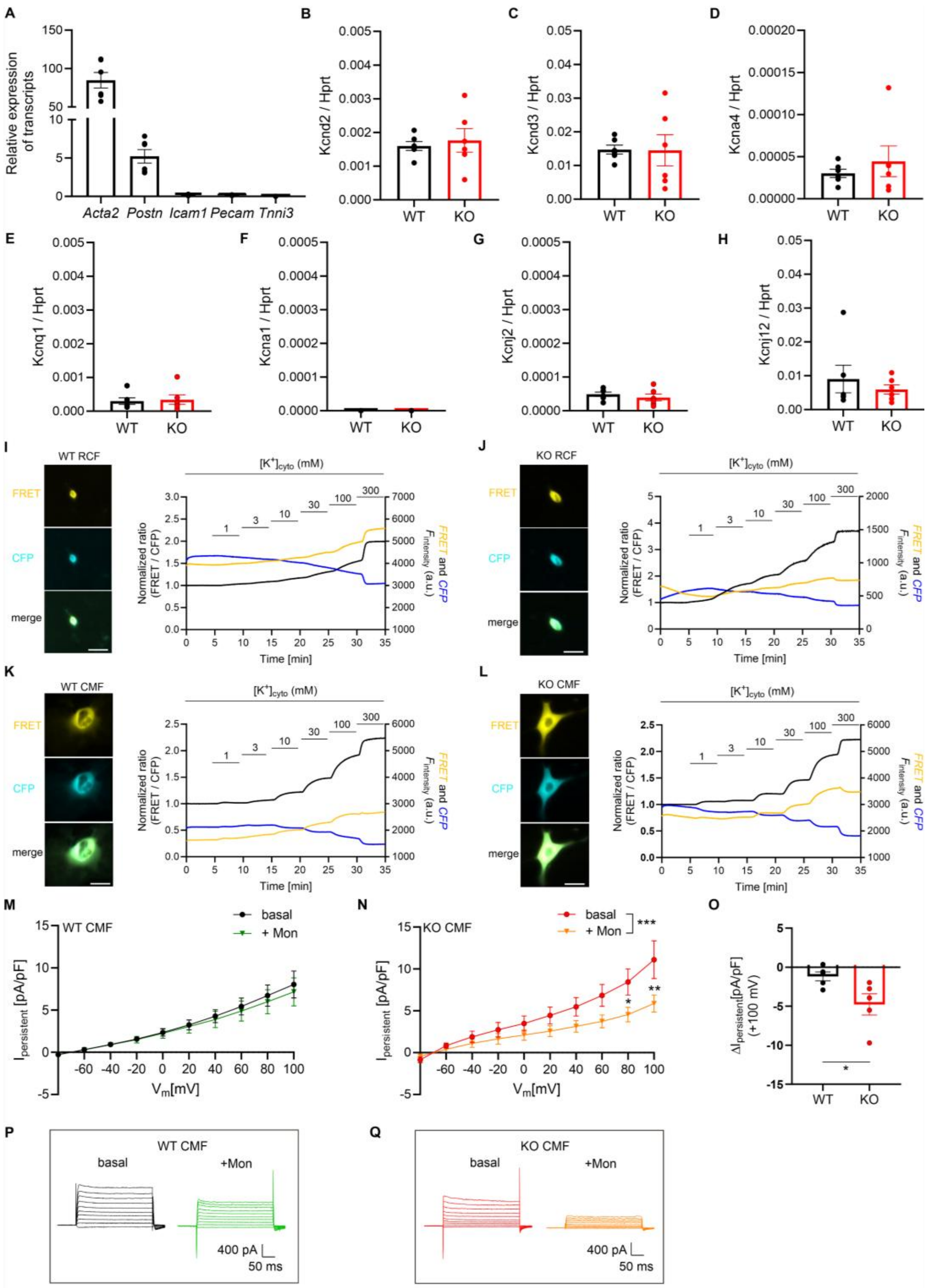


**Supplemental Figure 1:** *FRET imaging and electrophysiological characterization of Slick-mediated K*$^+$ *currents*

(**A**) RT-qPCR analysis of *Acta2* and *Postn* in RCF-derived CMFs (DIV10); *Icam1*, *Pecam*, and *Tnni3* were minimally detected. n = 6 hearts. (**B-H**) RT-qPCR analysis of K$^+$ channel transcripts in WT and KO CMFs. n = 6 hearts. (B, C, E, G) were analyzed with two-tailed unpaired Student's t-test and (D, H) were analyzed

with the Mann-Whitney *U* test. (**I-L**) Representative $[K^+]_{cyto}$ measurements in WT and KO RCFs and CMFs using GEPII 1.0 during stepwise increase of $[K^+]_{ex}$ (1-300 mM) with gramicidin permeabilization. Scale bar: 50 µm. (**M, N**) I-V relationship of persistent currents following monensin compared to baseline in WT and KO CMFs. Two-way ANOVA followed by Šídák's comparisons test, ***$P$ < 0.001 (**O**) Quantification of the Δ current at +100 mV. Nested t-test, *$P$ < 0.01 (n = 23 WT cells, n = 16 KO cells from 5 hearts for both genotypes). (**P**) Representative WT and (**Q**) KO current traces before and after monensin treatment.

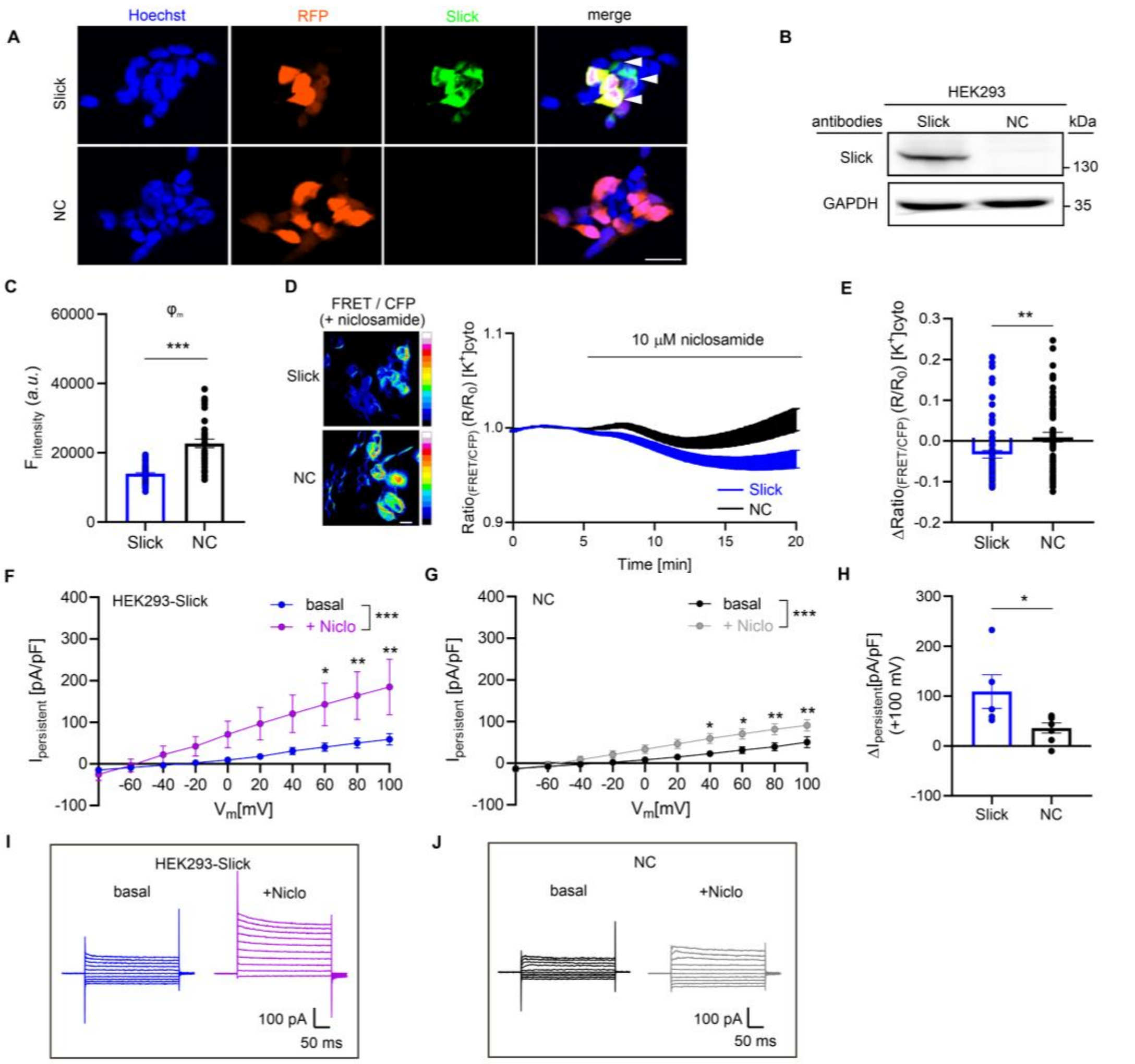


**Supplemental Figure 2:** *Overexpression of Slick in HEK293 cells*

(**A**) Representative IF images of HEK293 cells co-transfected with *pcDNA3.1-Slick* and *pcDNA3.1-mRuby3* or *pcDNA3.1-mRuby3* alone (NC). Scale bar: 25 µm. (**B**) Western blot analysis of Slick protein expression in HEK293 cells. GAPDH served as a loading control. (**C**) $\varphi_m$ measurements assessed using $DiBAC_4(3)$. Two-tailed unpaired Student's t-test, ****P* < 0.001 (n = 79 cells for the Slick-overexpressing group, n = 36 cells for NC group). (**D**) Representative FRET-based $[K^+]_{cyto}$ measurements in HEK293 cells with niclosamide perfusion. Scale bar: 25 µm. Normalized average FRET / CFP ratio over time is shown at right. (**E**) Quantification of Δ FRET / CFP ratios. Mann-Whitney *U* test, ***P* < 0.01 (n = 67 cells for Slick-overexpressing group, n = 61 cells for NC). (**F, G**) I-V relationships of HEK293-Slick and NC cells before and after niclosamide treatment. Two-way ANOVA followed by Šídák's multiple comparisons test. (**H**) Δ current density at + 100 mV (post-treatment minus baseline). Two-tailed unpaired Student's t-test, **P* < 0.05 (n = 5 cells for Slick-overexpressing HEK293, n = 7 cells for NC, from 4 independent preparations). (**I, J**) Representative I-V traces before and after niclosamide treatment in HEK293-Slick and NC cells.

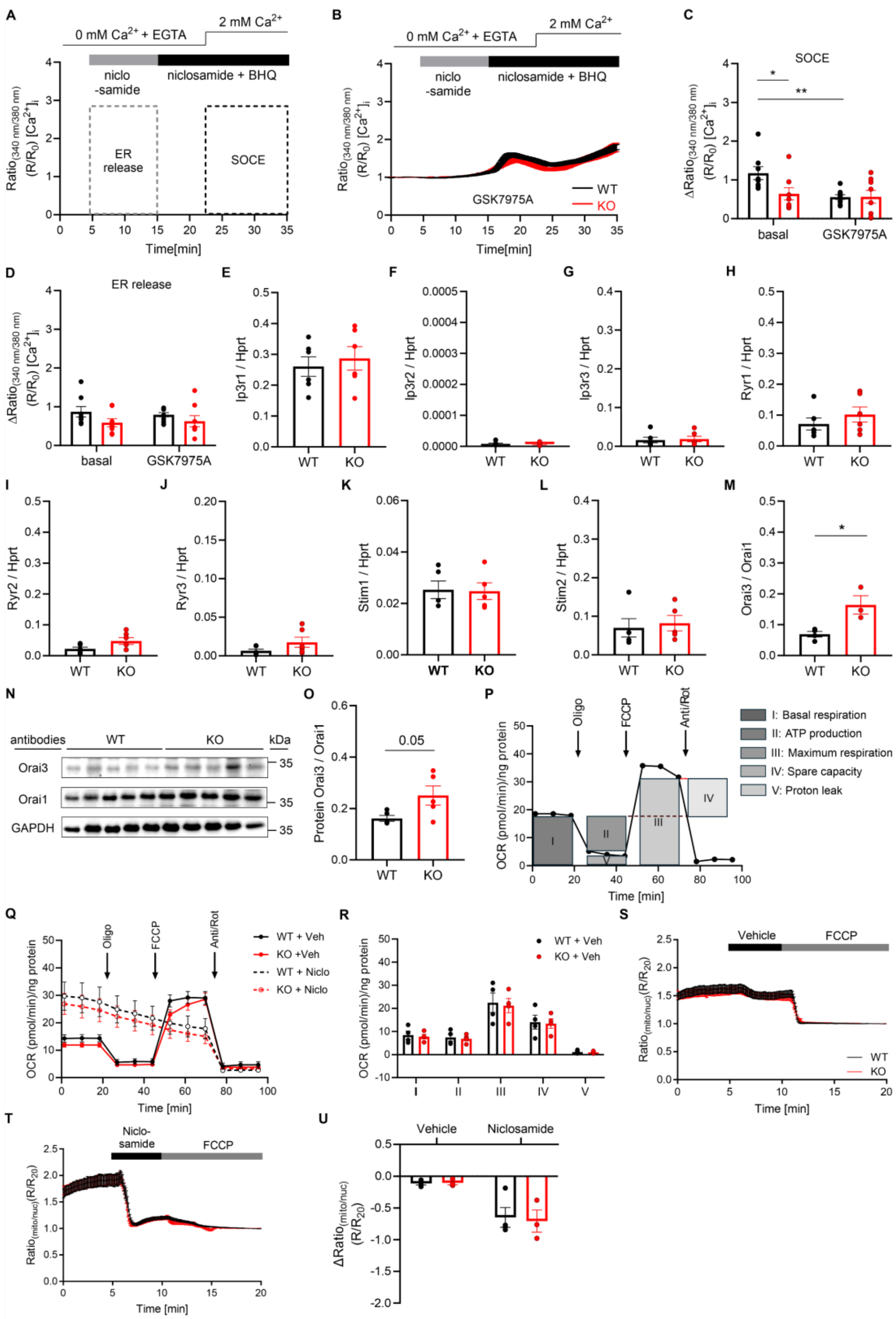


**Supplemental Figure 3:** *Orai remodeling and SOCE analysis in WT and KO CMFs*

(**A**) Schematic protocol for SOCE measurements. (**B**) Normalized average 340 nm / 380 nm ratio illustrating SOCE in WT and KO CMFs during GSK7975A treatment. (**C**) Quantification of $\Delta[Ca^{2+}]_i$ after GSK7975A treatment relative to basal measurement (Figure 2L). Two-way ANOVA with Tukey's multiple comparisons, ***P* < 0.01. (**D**) ER $Ca^{2+}$ release before or after GSK7975A treatment. Kruskal-Wallis test

with Dunn's multiple comparisons test. (n = 9 hearts for WT and n = 8 hearts for KO). (**E-J**) RT-qPCR analysis of *Ip3r* and *Ryr* isoforms in CMFs. (E, F, H, I, J) were analyzed by two-tailed unpaired Student's t-test and (G) by Mann-Whitney *U* test (n = 6 hearts per genotype). (**K, L**) RT-qPCR analysis of *Stim1* (two-tailed unpaired Student's t-test) and *Stim2* (Mann-Whitney *U* test) (n = 5 hearts per genotype). **(M**) Orai3 / Orai1 mRNA ratio. Two-tailed unpaired Student's t-test, $*P < 0.05$ (n = 4 hearts for WT, n = 3 hearts for KO). (**N**) Representative Western blot images of Orai3 and Orai1. After detecting Orai3 and Orai1 proteins, the membranes were stripped for GAPDH identification and quantification. (**O**) Orai3 / Orai1 protein ratio. Two-tailed unpaired Student's t-test. $P = 0.05$ (n = 5 hearts per genotype). (**P**) Schematic OCR protocol including application of oligomycin at 20 min, FCCP at 44 min, and antimycin A with rotenone at 68 min. (**Q**) OCR curves in WT and KO CMFs with vehicle or niclosamide treatment, normalized to the total protein. (**R**) Vehicle-treated comparison of OCR. Two-way ANOVA followed by Tukey's multiple comparisons test (n = 4 hearts per genotype). (**S-U**) Normalized intensity ratio of mitochondria to nucleus ($F_{mito}/F_{nuc}$) under niclosamide or DMSO treatment. Δ values ($R_{10}$-$R_0$) analyzed by Kruskal-Wallis test with Dunn's multiple comparisons test (n = 4 hearts per group).

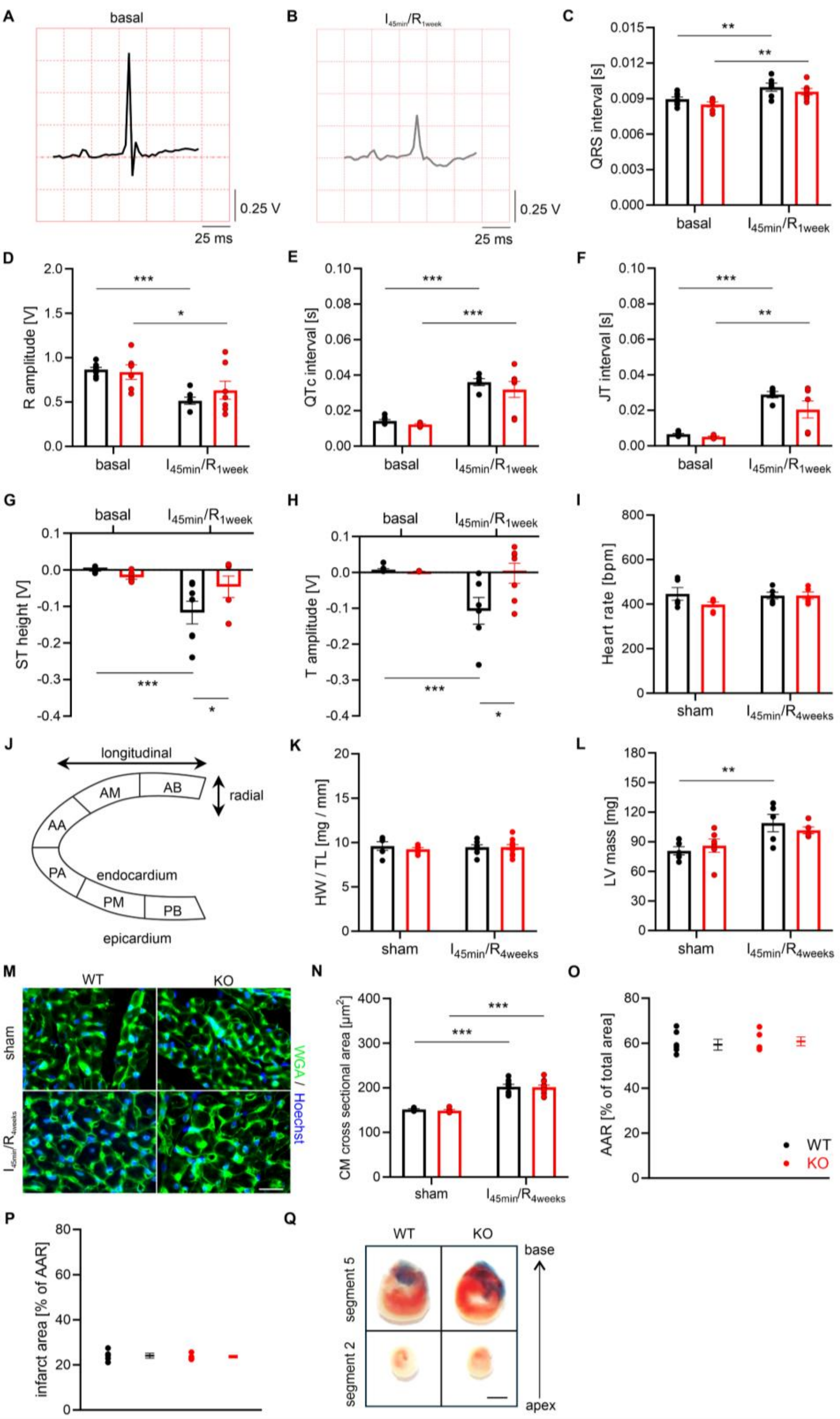


**Supplemental Figure 4:** *Characterization of WT and KO hearts following acute/chronic I/R injuries*

(**A-H**) Non-invasive ECG recordings in basal and $I_{45min}/R_{1week}$-exposed mice. Representative traces shown in (A, B). QRS interval was prolonged after I/R (**$P$ < 0.01), with no genotype difference. R-wave amplitude, QTc, and JT intervals showed similar changes. ST-segment height and T-wave amplitude differed between genotypes (*$P$ < 0.05). Two-way ANOVA with Tukey's multiple comparisons test (n = 8

for WT basal and n = 6 for KO basal hearts; and n = 7 for I/R hearts per genotype). (**I**) HR assessed by echocardiography. (**J**) Schematic of LV segmentation for B-mode analysis. (**K**) HW / TL ratio in sham and $I_{45min}/R_{4weeks}$ groups (n = 5 hearts for sham WT, n = 6 hearts for sham KO, n = 8 hearts for $I_{45min}/R_{4weeks}$ WT, and n = 10 hearts for $I_{45min}/R_{4weeks}$ KO). (**L**) LV mass assessed by M-mode echocardiography. Two-way ANOVA with Tukey's multiple comparisons test, $^*P < 0.05$. **(M, N)** Representative WGA staining and quantification of CSA in sham and $I_{45min}/R_{4weeks}$ hearts. Two-way ANOVA with Tukey's multiple comparisons test, $^*P < 0.05$ (n = 6 for sham WT; n = 5 for sham KO; n = 8 hearts for $I_{45min}/R_{4weeks}$ WT and n = 11 for $I_{45min}/R_{4weeks}$ KO). Scale bar: 20 µm. (**O, P**) Quantification of AAR and infarct area in acute I/R settings ($I_{30min}/R_{2h}$). No significant difference between WT and KO was observed. Two-tailed unpaired Student's t-test (n = 6 hearts for WT, n = 5 hearts for KO). (**Q**) Representative Evans Blue and TTC staining of $I_{30min}/R_{2h}$-exposed hearts. Scale bar: 2 mm.

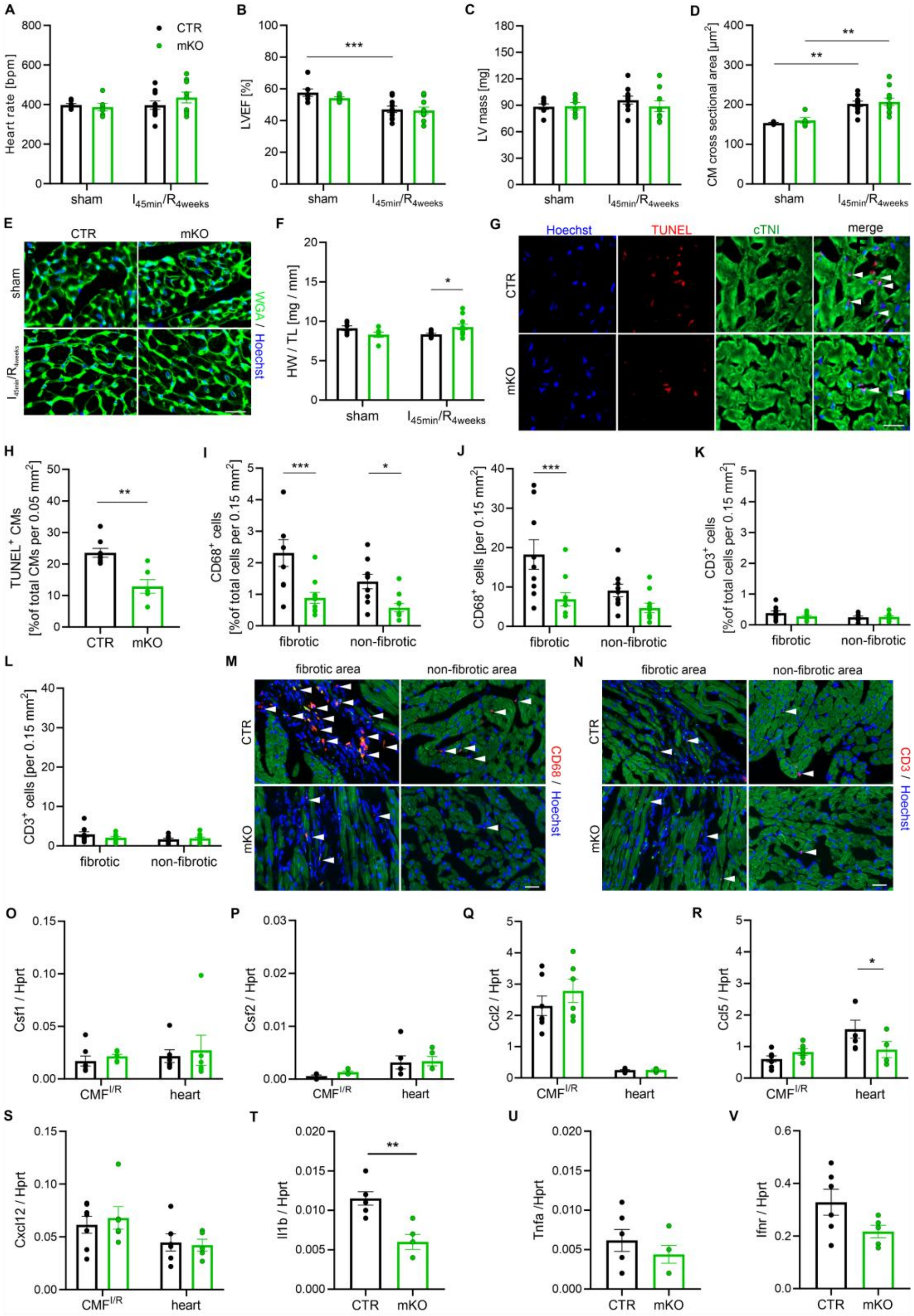


**Supplemental Figure 5:** *Characterization of CTR and mKO hearts and CMFs upon I/R injury*

(**A**) HR assessed by echocardiography. (**B**) LVEF measured by B-mode echocardiography, CTR hearts exhibited reduced LVEF after I/R. Two-way ANOVA with Tukey's multiple comparisons, ***$P < 0.001$. (**C**) LV mass assessed by M-mode echocardiography. (**D, E**) CSA quantification and representative WGA staining (n = 4 hearts for sham CTR, n = 5 hearts for sham mKO, n = 10 hearts for $I_{45min}/R_{4weeks}$ CTR, and n = 11 hearts for $I_{45min}/R_{4weeks}$ mKO). Scale bar: 20 µm. (**F**) HW / TL ratio. Two-way ANOVA with Tukey's

multiple comparisons, *$P < 0.05$ (n = 6 hearts for sham CTR, n = 6 hearts for sham mKO, n = 9 hearts for $I_{45min}/R_{4weeks}$ CTR, n = 11 hearts for $I_{45min}/R_{4weeks}$ mKO). (**G, H**) Representative TUNEL staining and quantification of TUNEL-positive cells. Mann-Whitney *U* test, **$P < 0.01$ (n = 8 hearts for CTR, n = 6 hearts for mKO). Scale bar: 20 µm. (**I-L**) Frequency of and absolute counts of $CD68^+$ macrophages and $CD3^+$ T cells in fibrotic and non-fibrotic regions of I/R hearts. Two-way ANOVA with Tukey's multiple comparisons. (**M, N**) Representative IF images of CD68 and CD3. CMs exhibit green autofluorescence, distinguishing fibrotic regions and viable myocardium. Positively stained cells indicated by white arrowheads. Scale bar: 50 µm. (**O-S**) RT-qPCR analysis of cytokine and chemokine transcripts in $CMF^{I/R}$ and whole-heart lysates. *Ccl5* was increased in CTR hearts. Two-way ANOVA with Tukey's multiple comparisons, *$P < 0.05$ (n = 7 for CTR $CMF^{I/R}$, n = 6 for mKO $CMF^{I/R}$, and n = 6 for both CTR and mKO heart lysates). **(T-V)** RT-qPCR analysis of pro-inflammatory cytokine transcripts in whole heart lysates. *Il1b* was elevated in CTR hearts. Two-tailed unpaired Student's t-test, **$P < 0.01$. (n = 6 per genotype).

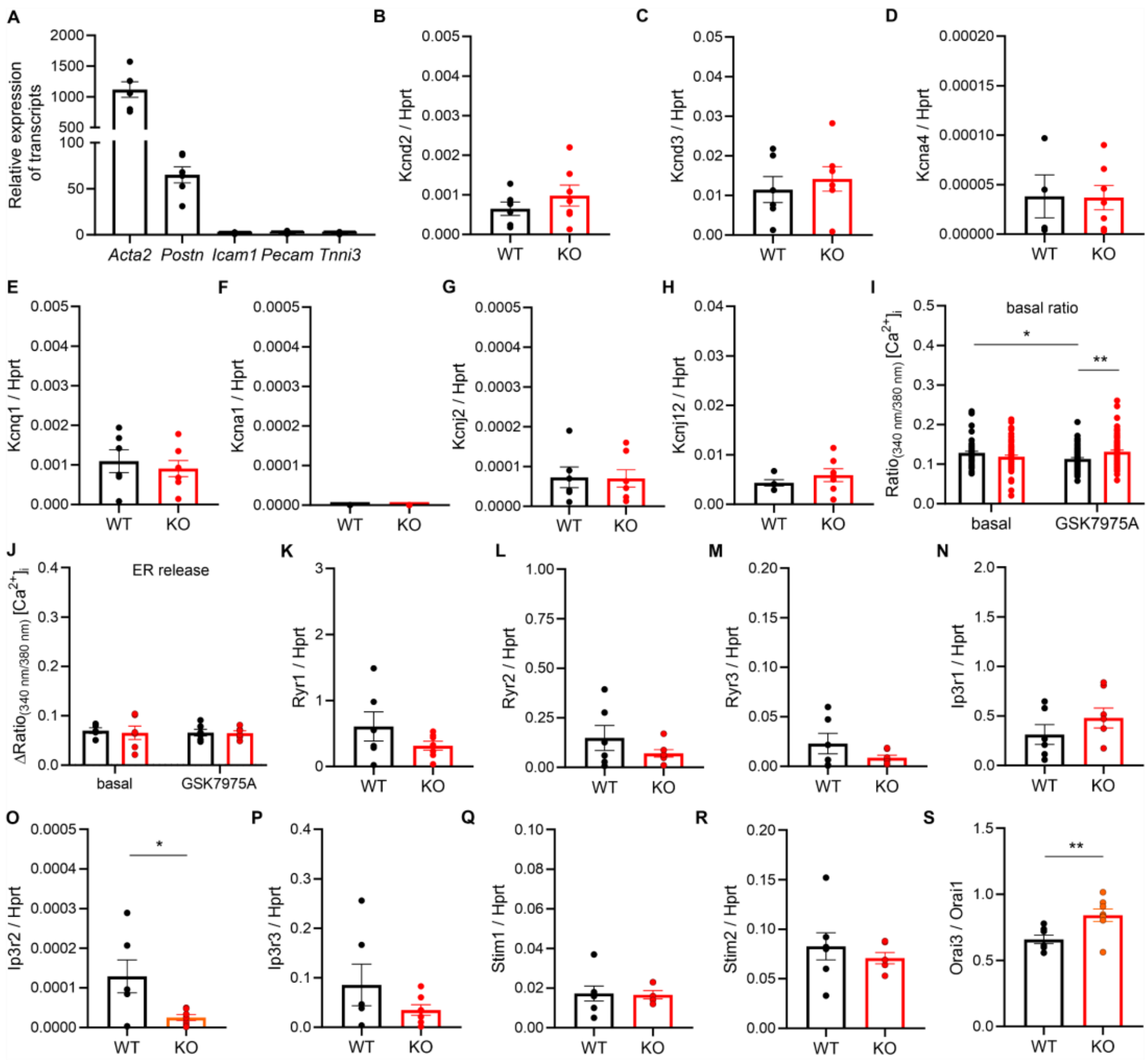


**Supplemental Figure 6:** *$K^+$ channel expression profile and $Ca^{2+}$ dynamics in $CMF^{I/R}$*

(**A**) RT-qPCR analysis of fibrotic transcripts in $CMF^{I/R}$. $CMF^{I/R}$ highly expressed *Acta2* and *Postn*, whereas *Icam1*, *Pecam*, and *Tnni3* were minimally detected. (**B-H**) RT-qPCR analysis of $K^+$ channel transcripts in $CMF^{I/R}$ revealed no difference (n = 6 for WT $CMF^{I/R}$ and n = 7 for KO $CMF^{I/R}$). (**I**) Basal $[Ca^{2+}]_i$ ratio ($R_0$) corresponding to SOCE measurements shown in Figure 5D, E. GSK7975A reduced basal $[Ca^{2+}]_i$ in WT (**P* < 0.05) $CMF^{I/R}$. WT exhibited lower basal $[Ca^{2+}]_i$ than KO after GSK7975A treatment (***P* < 0.01). (**J**) ER $Ca^{2+}$ levels did not differ between genotypes with or without GSK7975A. Two-way ANOVA with Tukey's multiple comparisons. (**K-M**) RT-qPCR analysis of *Ryr1-3* transcripts in WT and KO $CMF^{I/R}$. (**N-P**) *Ip3r1* and *Ip3r3* expression did not differ between genotypes. *Ip3r2* was higher in WT than KO $CMF^{I/R}$, **P* < 0.05 (n = 6 hearts for WT $CMF^{I/R}$ and n = 7 for KO $CMF^{I/R}$). (**Q, R**) *Stim1* and *Stim2* expression remained unchanged (n = 7 hearts for WT $CMF^{I/R}$ and n = 6 for KO $CMF^{I/R}$). (**S**) *Orai3*/*Orai1* transcript ratio was increased in KO $CMF^{I/R}$. Two-tailed unpaired Student's t-test, **P* < 0.01 (n = 7 hearts for WT $CMF^{I/R}$ and n = 8 for KO $CMF^{I/R}$).

**Supplemental Table 1: Primer sequences of the detected transcripts**

| **Primers** | **Sequences** |
|---|---|
| *Acta2*$^{for}$ | 5’-AGA GGC ACC ACT GAA CCC TA-3’ |
| *Acta2*$^{rev}$ | 5’-GCA TAG AGG GAC AGC ACA GC-3’ |
| *Ccl2*$^{for}$ | 5'-TGA CCC CAA GAA GGA ATG GG-3' |
| *Ccl2*$^{rev}$ | 5'-GAC CTT AGG GCA GAT GCA GTT-3' |
| *Ccl5*$^{for}$ | 5'-TTC CCT GTC ATT GCT TGC TCT-3' |
| *Ccl5*$^{rev}$ | 5'-CCC ATT TTC CCA GGA CCG AG-3' |
| *Col3a*$^{for}$ | 5’-GGT CCT CCA GGA GAA AAT GG-3’ |
| *Col3a*$^{rev}$ | 5’-GTG CAC CAG AAT CAC CCT TG-3’ |
| *Csf1*$^{for}$ | 5'-TCC CAT AAA CCA CAT GCC CC-3' |
| *Csf1*$^{rev}$ | 5'-GAG CAC CGA GGC AAA CTT TC-3' |
| *Csf2*$^{for}$ | 5'-GCC AGG AGA TTC CAC AAC TCA-3' |
| *Csf2*$^{rev}$ | 5'-CTG GGC TCA CTG CAA AAG AG-3' |
| *Cxcl12*$^{for}$ | 5'-CCT TCA GAT TGT TGC ACG GC-3' |
| *Cxcl12*$^{rev}$ | 5'-CTT GCA TCT CCC ACG GAT GT-3' |
| *Hprt*$^{for}$ | 5’-CTG GTG AAA AGG ACC TCT CGA AG-3’ |
| *Hprt*$^{rev}$ | 5’-CCA GTT TCA CTA ATG ACA CAA ACG-3’ |
| *Icam1*$^{for}$ | 5’-CGG ACT TTC GAT CTT CCA GCT ACC-3’ |
| *Icam1*$^{rev}$ | 5’-CGA GCT TCA GAG GCA GGA AAC A-3’ |
| *Ifnr*$^{for}$ | 5'-ACA GCT CTC CGT CCT CGT AT-3' |
| *Ifnr*$^{rev}$ | 5'-CAC TCC GGT TAT GCT CCA CA-3' |

| | |
|---|---|
| *Ip3r1*$^{for}$ | 5'-GAG AGT TAC GTG GCA GAG ATG ATC-3' |
| *Ip3r1*$^{rev}$ | 5'-GGC CAG AAA GAT TGG TGA CC-3' |
| *Ip3r2*$^{for}$ | 5'-TAG TCC TGG TGA AAG TTA AAG ACC C-3' |
| *Ip3r2*$^{rev}$ | 5'-CAG ACT CAT GGT CGA TTC CAA CT-3' |
| *Ip3r3*$^{for}$ | 5'-TGT ACT TCA TTG TGC TGG TCC G-3' |
| *Ip3r3*$^{rev}$ | 5'-CGA ATC TCA TTC TGC TCC CC-3' |
| *Kcna1*$^{for}$ | 5'-AGT GAC TCA TGT CAC GCT TTG T-3' |
| *Kcna1*$^{rev}$ | 5'-CTG GTT CAC CAC CCC CAA AA-3' |
| *Kcna4*$^{for}$ | 5'-GAA CCT TGA CTT CCG TTC CCA-3' |
| *Kcna4*$^{rev}$ | 5'-AGG GGG CTA GGG GGT ATT G-3' |
| *Kcnd2*$^{for}$ | 5'-TGC TCA AGG AGT TCA CGG TC-3' |
| *Kcnd2*$^{rev}$ | 5'-TCT GTT AGG CGA GCA CCA AG-3' |
| *Kcnd3*$^{for}$ | 5'-GTG ACA ATC CCA CTG TCT CCA-3' |
| *Kcnd3*$^{rev}$ | 5'-ATG GCC TAG GCA TGT TTG GTG-3' |
| *Kcnj2*$^{for}$ | 5'-CGG CGA GAG TCG GAG ATA TG-3' |
| *Kcnj2*$^{rev}$ | 5'-CGC TTG CTT GAC CCA TCT TG-3' |
| *Kcnj12*$^{for}$ | 5'-GGT CAC TTG GTG CTG GTG TA-3' |
| *Kcnj12*$^{rev}$ | 5'-GCT GGG AGG TAA GAG CAC AG-3' |
| *Kcnq1*$^{for}$ | 5'-GGG TCT CCA TCT ACA GTG CG-3' |
| *Kcnq1*$^{rev}$ | 5'-TGA GGA AGA CGG TGA AGT GG-3' |
| *Kcnt2*$^{for}$ | 5’-TCT ATT TGA AAC AAT ACT CCT TGG-3’ |
| *Kcnt2*$^{rev}$ | 5’-GAA CAA ATA GAT TTC TTA AGG TGG-3’ |

| | |
|---|---|
| *Orai1*[for] | 5'-AAC GAG CAC TCG ATG CAG G-3' |
| *Orai1*[rev] | 5'-GGG TAG TCA TGG TCT GTG TCC-3' |
| *Orai3*[for] | 5'-GGC TAC CTG GAC CTT ATG G-3' |
| *Orai3*[rev] | 5'-GGT GGG TAT TCA TGA TCG TT-3' |
| *Pecam*[for] | 5'-TTG CAG TCA GAG TCT TCC TTG CC-3' |
| *Pecam*[rev] | 5'-GGG TTT CTG TTT GGC CTT GGC T-3' |
| *Postn*[for] | 5'-CCA TTG GAG GCA AAC AAC TCC-3' |
| *Postn*[rev] | 5'-TTG CTT CCT CTC ACC ATG CA-3' |
| *Ryr1*[for] | 5'-CCA AGG CAG GAG ACG TAC AG-3' |
| *Ryr1*[rev] | 5'-CAA GGA TGT CTG CAC GGA GT-3' |
| *Ryr2*[for] | 5'-CTG CCC TCT GAA GAC CTG AC-3' |
| *Ryr2*[rev] | 5'-TAC CTC AGG GAC AGG GAC AG-3' |
| *Ryr3*[for] | 5'-GAC AGG ACC AGG AAC GGA AG-3' |
| *Ryr3*[rev] | 5'-TTC AGA CCA ATG GGC AGC AT-3' |
| *Stim1*[for] | 5'-TGT GGA GCT GCC ACA GTA TG-3' |
| *Stim1*[rev] | 5'-AAG AGA GGA GGC CCA AAC AG-3' |
| *Stim2*[for] | 5'-TAG TCA CCT GCA CAG AGA AG-3' |
| *Stim2*[rev] | 5'-AGT TTC ATG AAC TGC TAT CC-3' |
| *Tcf21*[for] | 5'-TTT CCA CTT TTT GGG GTG CG-3' |
| *Tcf21*[rev] | 5'-GTG TAC CGC TAG GTC CAA CA-3' |
| *Tgfb1*[for] | 5'-TGG AGC AAC ATG TGG AAC TC-3' |
| *Tgfb1*[rev] | 5'-CAG CAG CCG GTT ACC AAG-3' |

| | |
|---|---|
| *Tnfa*$^{\text{for}}$ | 5'-GTA GCC CAC GTC GTA GCA AA-3' |
| *Tnfa*$^{\text{rev}}$ | 5'-ACA AGG TAC AAC CCA TCG GC-3' |
| *Tnni3*$^{\text{for}}$ | 5’-AGA GCT TCA GGA CTT ATG CCG ACA-3’ |
| *Tnni3*$^{\text{rev}}$ | 5’-TGG TGA CTT TTG CTT CCA CGT C-3’ |

**Supplemental Table 1**: *Primer pairs used in this study to detect murine genes*
Superscription of “for” stands for the forward primer, and “rev” indicates the reverse primer.

**Supplemental references**

1. Frankenreiter S, et al. cGMP-Elevating Compounds and Ischemic Conditioning Provide Cardioprotection Against Ischemia and Reperfusion Injury via Cardiomyocyte-Specific BK Channels. *Circulation.* 2017;136(24):2337-55.

2. Roslan A, et al. Slack K+ channels confer protection against myocardial ischemia/reperfusion injury. *Cardiovasc Res.* 2024.

3. Oestereicher MA, et al. Comprehensive ECG reference intervals in C57BL/6N substrains provide a generalizable guide for cardiac electrophysiology studies in mice. *Mamm Genome.* 2023;34(2):180-99.

4. Köhler D, et al. Targeting Gα(i2) in neutrophils protects from myocardial ischemia reperfusion injury. *Basic Res Cardiol.* 2024;119(5):717-32.

5. Merentie M, et al. Mouse ECG findings in aging, with conduction system affecting drugs and in cardiac pathologies: Development and validation of ECG analysis algorithm in mice. *Physiol Rep.* 2015;3(12).

6. Mitchell GF, et al. Measurement of heart rate and Q-T interval in the conscious mouse. *Am J Physiol.* 1998;274(3):H747-51.

7. Xie Y, et al. Immunohistochemical detection of differentially localized up-regulation of lysyl oxidase and down-regulation of matrix metalloproteinase-1 in rhesus monkey model of chronic myocardial infarction. *Exp Biol Med (Maywood).* 2012;237(7):853-9.

8. Alarcón B, et al. The CD3-gamma and CD3-delta subunits of the T cell antigen receptor can be expressed within distinct functional TCR/CD3 complexes.

*Embo j.* 1991;10(4):903-12.

9. Choudhary M, and Malek G. CD68: Potential Contributor to Inflammation and RPE Cell Dystrophy. *Adv Exp Med Biol.* 2023;1415:207-13.

10. Dai L, et al. Activation of Slo2.1 channels by niflumic acid. *J Gen Physiol.* 2010;135(3):275-95.

11. Biton B, et al. The antipsychotic drug loxapine is an opener of the sodium-activated potassium channel slack (Slo2.2). *J Pharmacol Exp Ther.* 2012;340(3):706-15.

12. Tsuchida K, et al. Monensin-Induced Increase in Intracellular Na+ Induces Changes in Na+ and Ca2+ Currents and Regulates Na+-K+ and Na+-Ca2+ Transport in Cardiomyocytes. *Pharmacology.* 2021;106(1-2):91-105.

13. Smith CO, et al. The Slo(w) path to identifying the mitochondrial channels responsible for ischemic protection. *Biochem J.* 2017;474(12):2067-94.

14. Gross D, et al. IK(Ca) channels control breast cancer metabolism including AMPK-driven autophagy. *Cell Death Dis.* 2022;13(10):902.

15. Huang JK, et al. Effect of thapsigargin on $Ca^{2}$+ fluxes and viability in human prostate cancer cells. *J Recept Signal Transduct Res.* 2011;31(3):247-55.

16. Nelson HA, et al. Interplay between ER Ca(2+) Binding Proteins, STIM1 and STIM2, Is Required for Store-Operated Ca(2+) Entry. *Int J Mol Sci.* 2018;19(5).

17. Chen PH, et al. Lithium Reduces Migration and Collagen Synthesis Activity in Human Cardiac Fibroblasts by Inhibiting Store-Operated Ca(2+) Entry. *Int J Mol Sci.* 2021;22(2).

18. Yu C, et al. Orai3 mediates Orai channel remodelling to activate fibroblast in pulmonary fibrosis. *J Cell Mol Med.* 2022;26(19):4974-85.

19. Bird GS, et al. Methods for studying store-operated calcium entry. *Methods.* 2008;46(3):204-12.

20. Bischof H, et al. mitoBK(Ca) is functionally expressed in murine and human breast cancer cells and potentially contributes to metabolic reprogramming. *Elife.* 2024;12.
