# Supplementary material for: Slick K+ channels contribute to cardiac remodeling, fibrosis, and dysfunction in postinfarction hearts
Source: JCI Insight. 2026 Mar 17;11(13):e195805. doi: 10.1172/jci.insight.195805 (PMC13460849; doi:10.1172/jci.insight.195805)

Unedited blot of Figure 5K

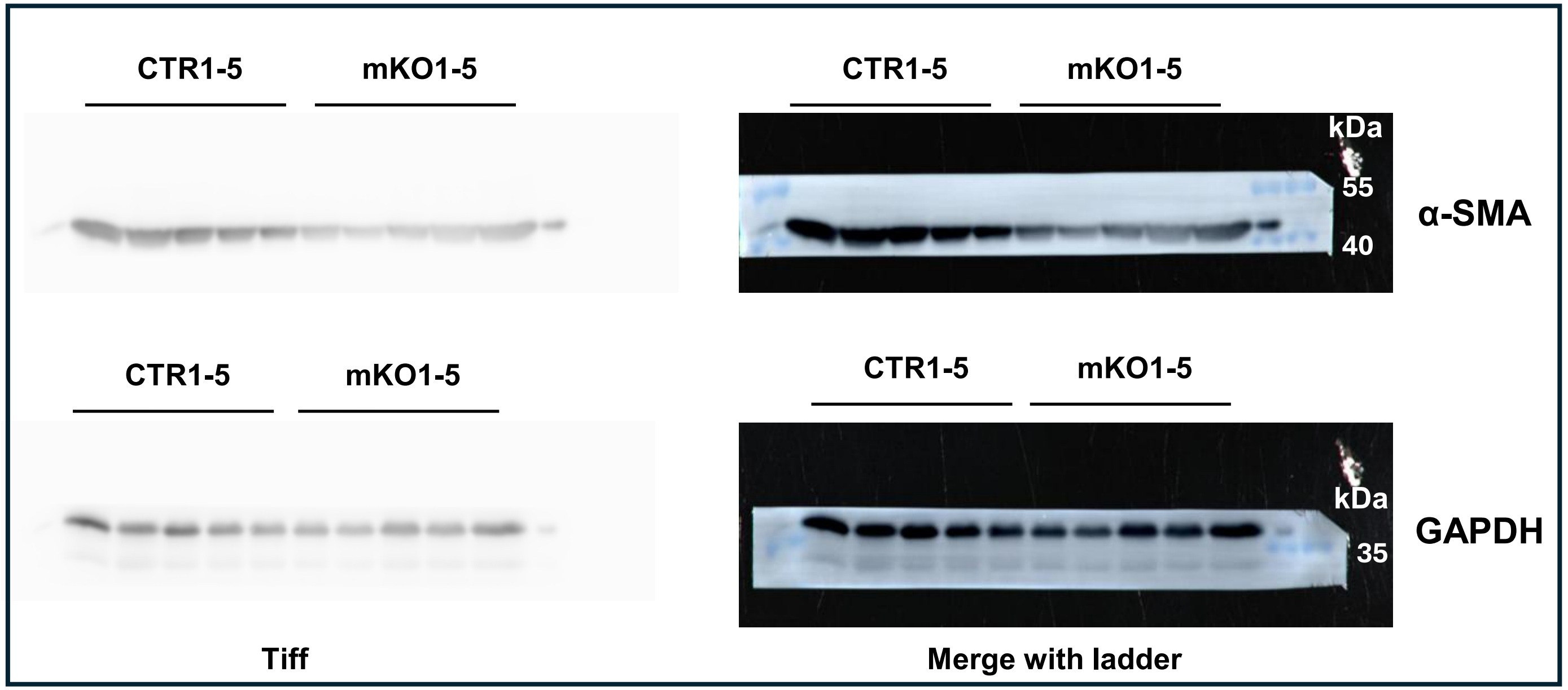

Unedited blot of SFigure 2B

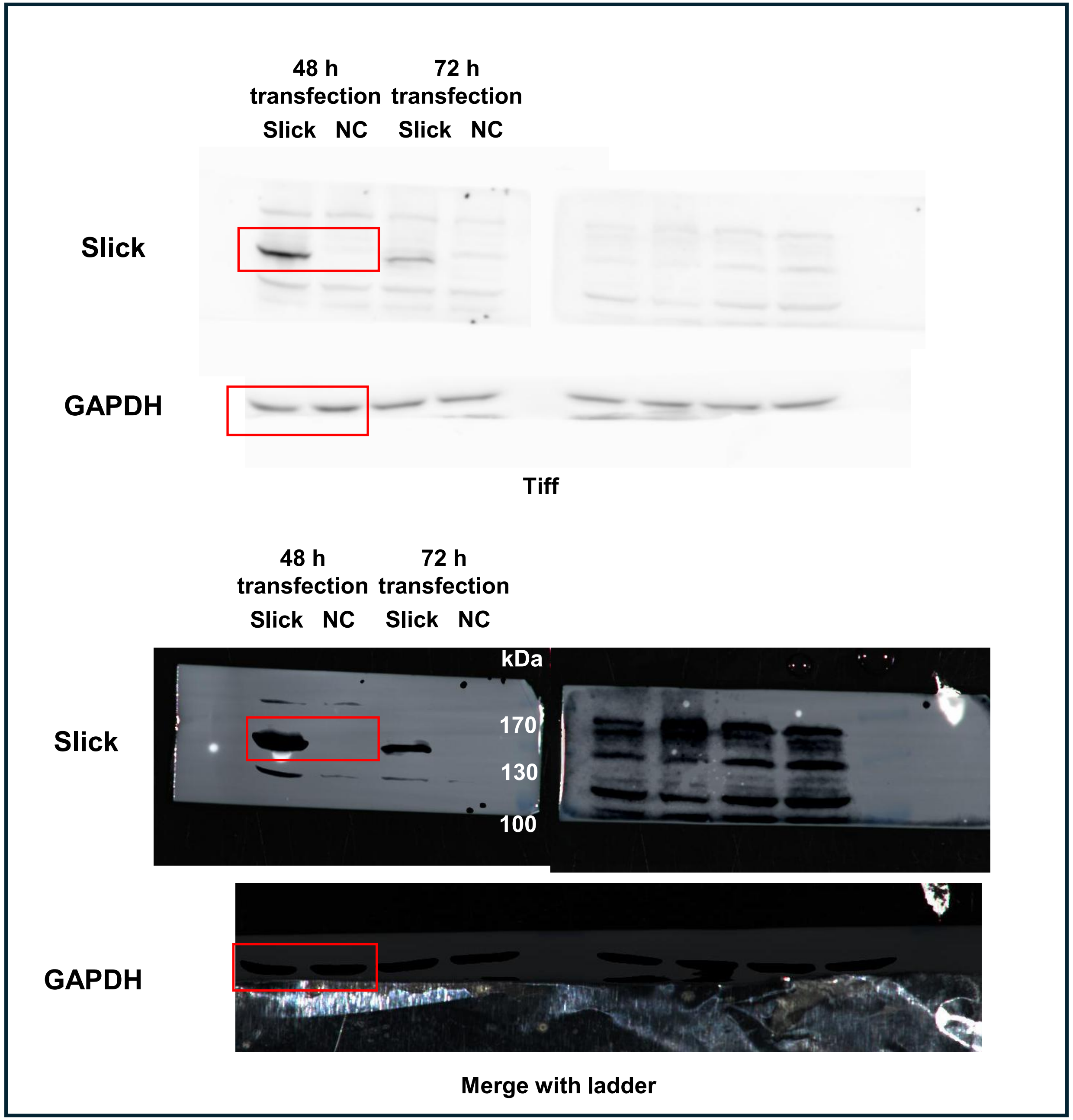

*: The upper membrane was cropped because the right section was used for a different primary antibody, which is not relevant to the results presented in this manuscript

**: We covered the lower part of the membrane with aluminum foil due to strong background signal resulting from the use of an 8% SDS-PAGE gel, which was necessary to separate high-molecular-weight proteins (Slick). However, this led to the accumulation of intense nonspecific signals in the lowest region of the membrane, which interfered with GAPDH detection. To optimize exposure and enhance GAPDH signal clarity, this area was masked during imaging. The image shown below represents the original membrane prior to masking; the red arrow indicates the nonspecific signal.

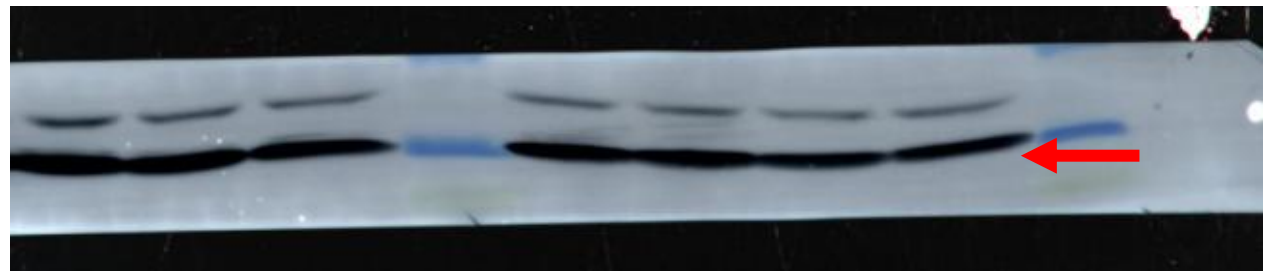

Unedited blot of SFigure 3N

Orai1

WT1-5 KO1-5

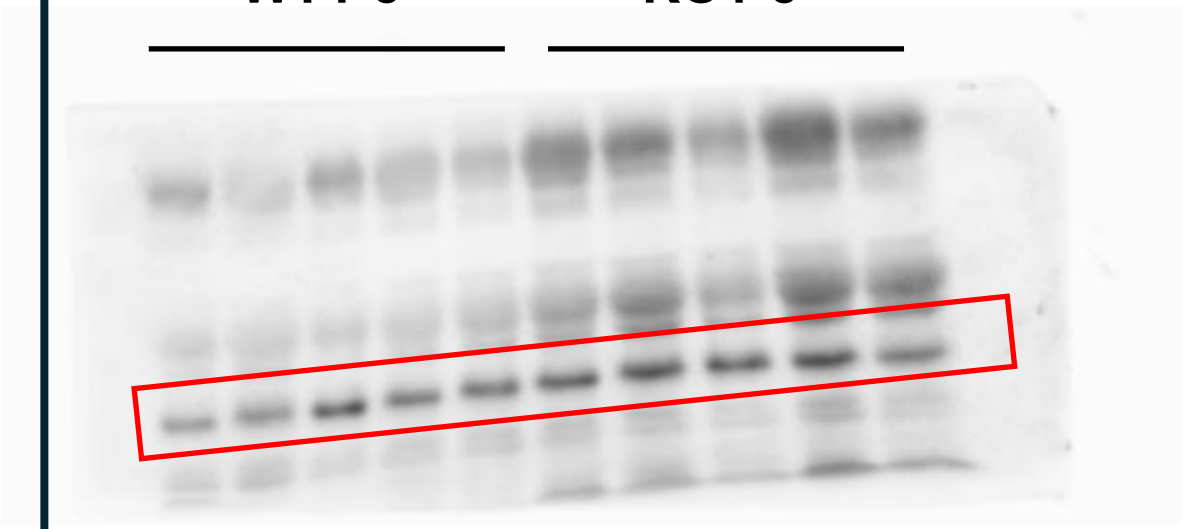

Tiff

WT1-5 KO1-5

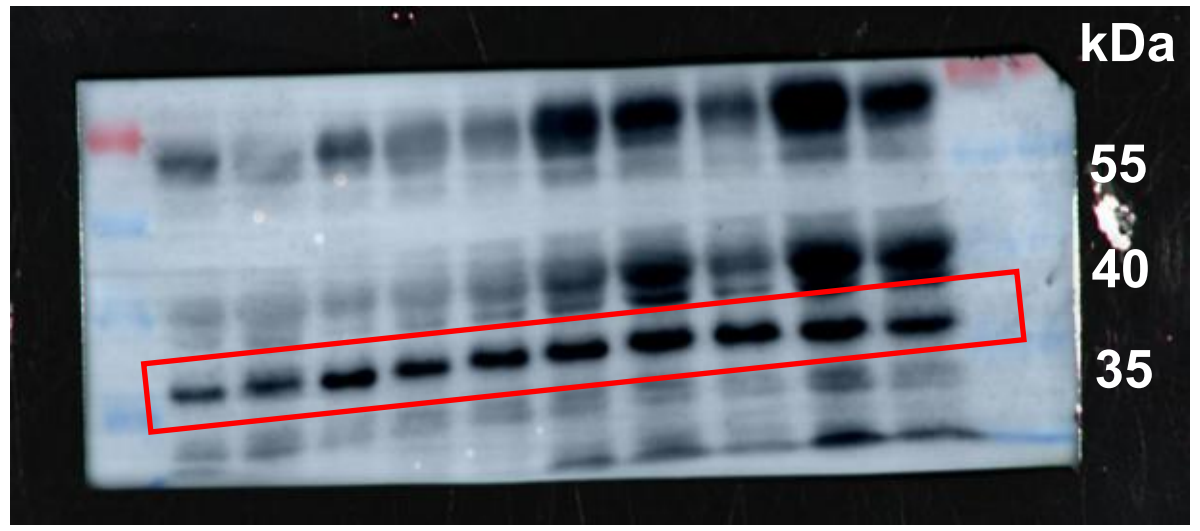

Merge with ladder

GAPDH

WT1-5 KO1-5

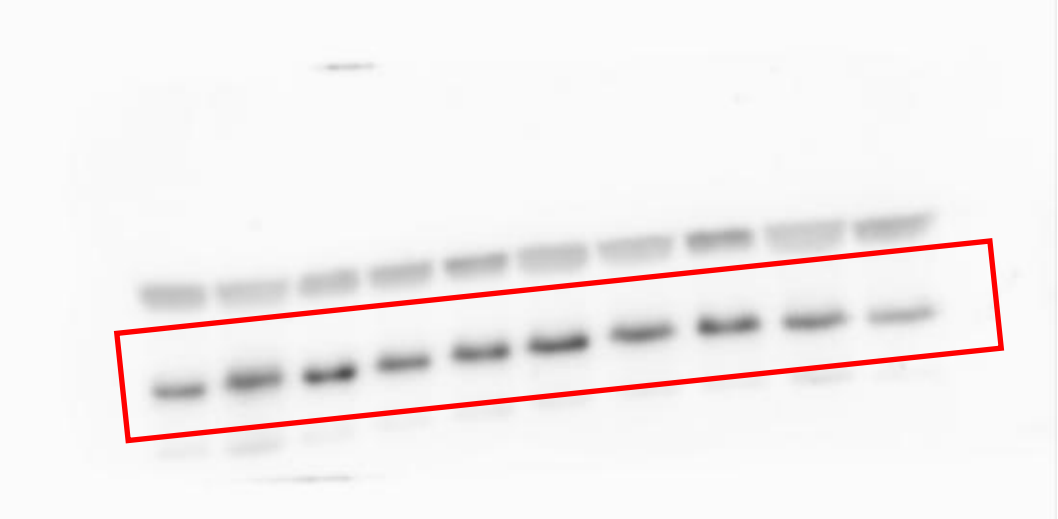

Tiff

WT1-5 KO1-5

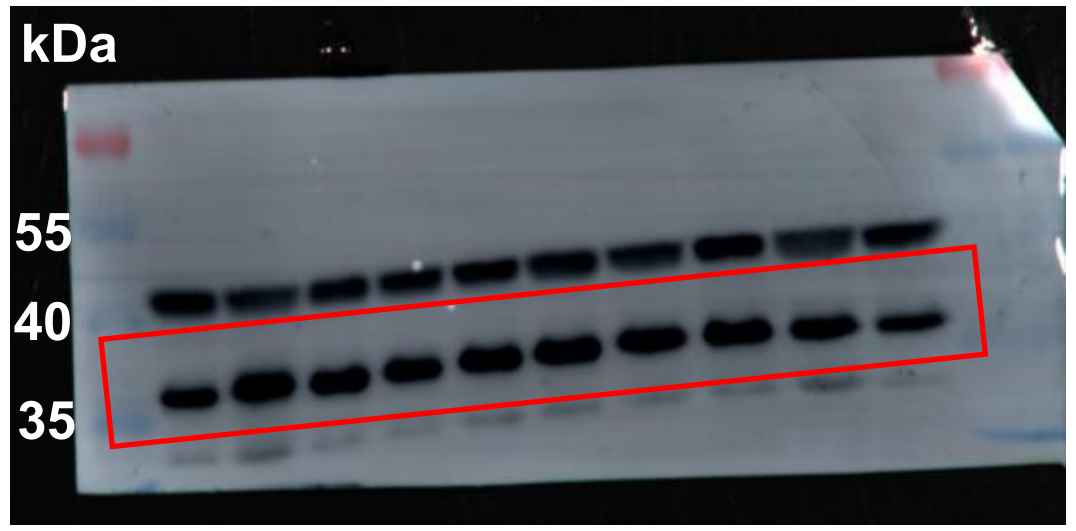

Merge with ladder

Orai3

KO1-5 WT1-5

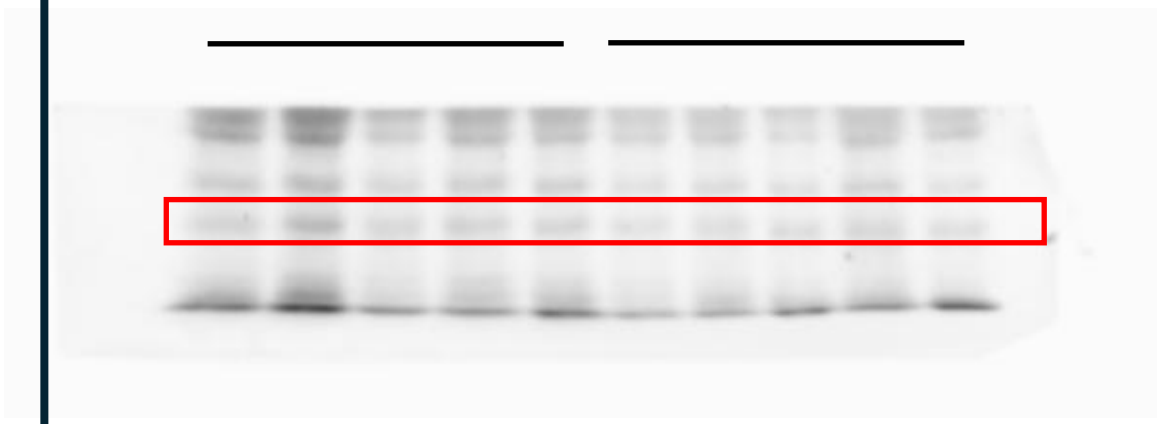

Tiff

KO1-5 WT1-5

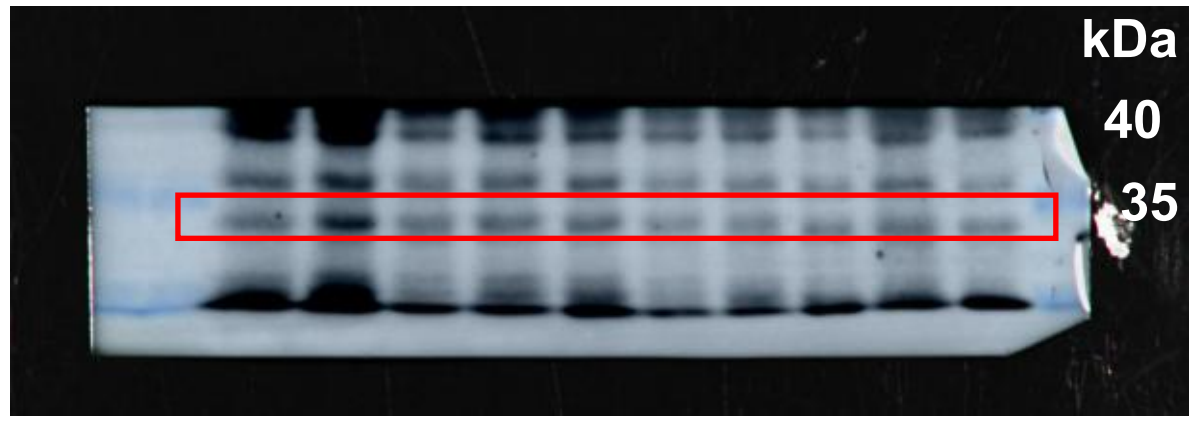

Merge with ladder

GAPDH

KO1-5 WT1-5

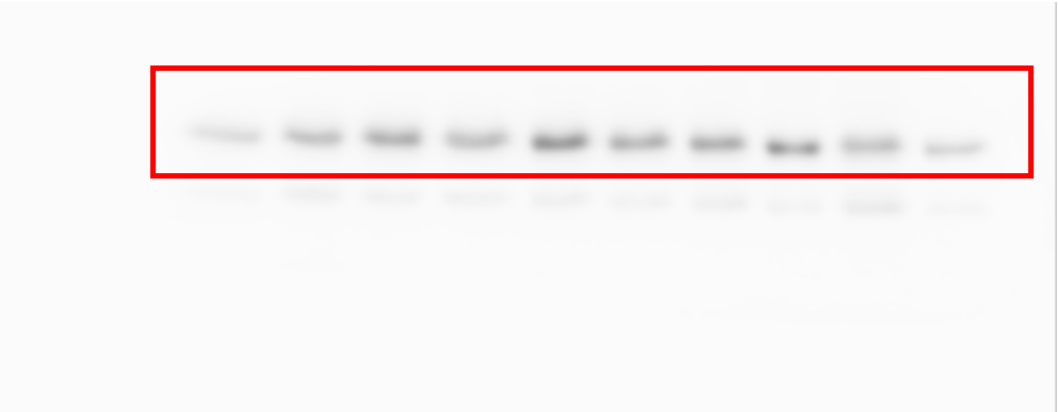

Tiff

KO1-5 WT1-5

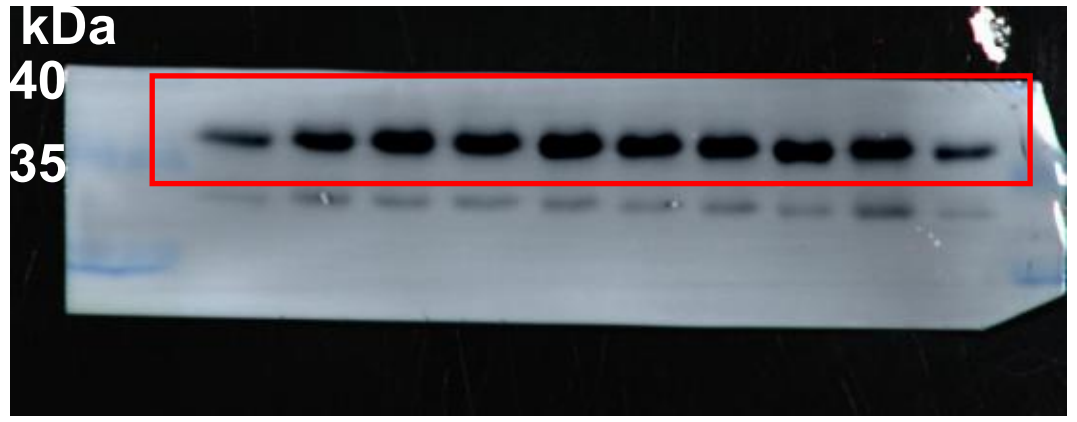

Merge with ladder

Unedited gel image of Figure 4B

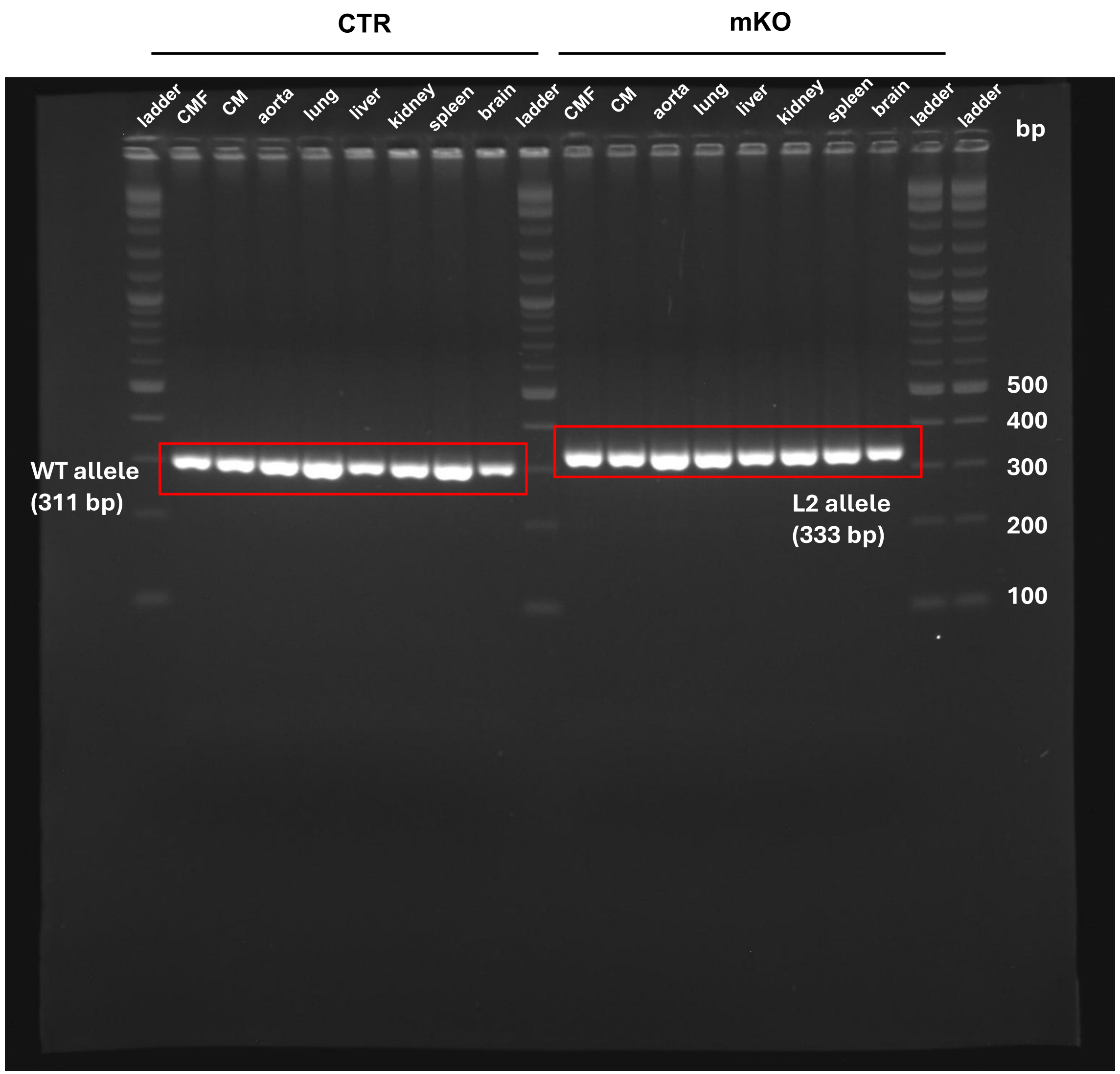

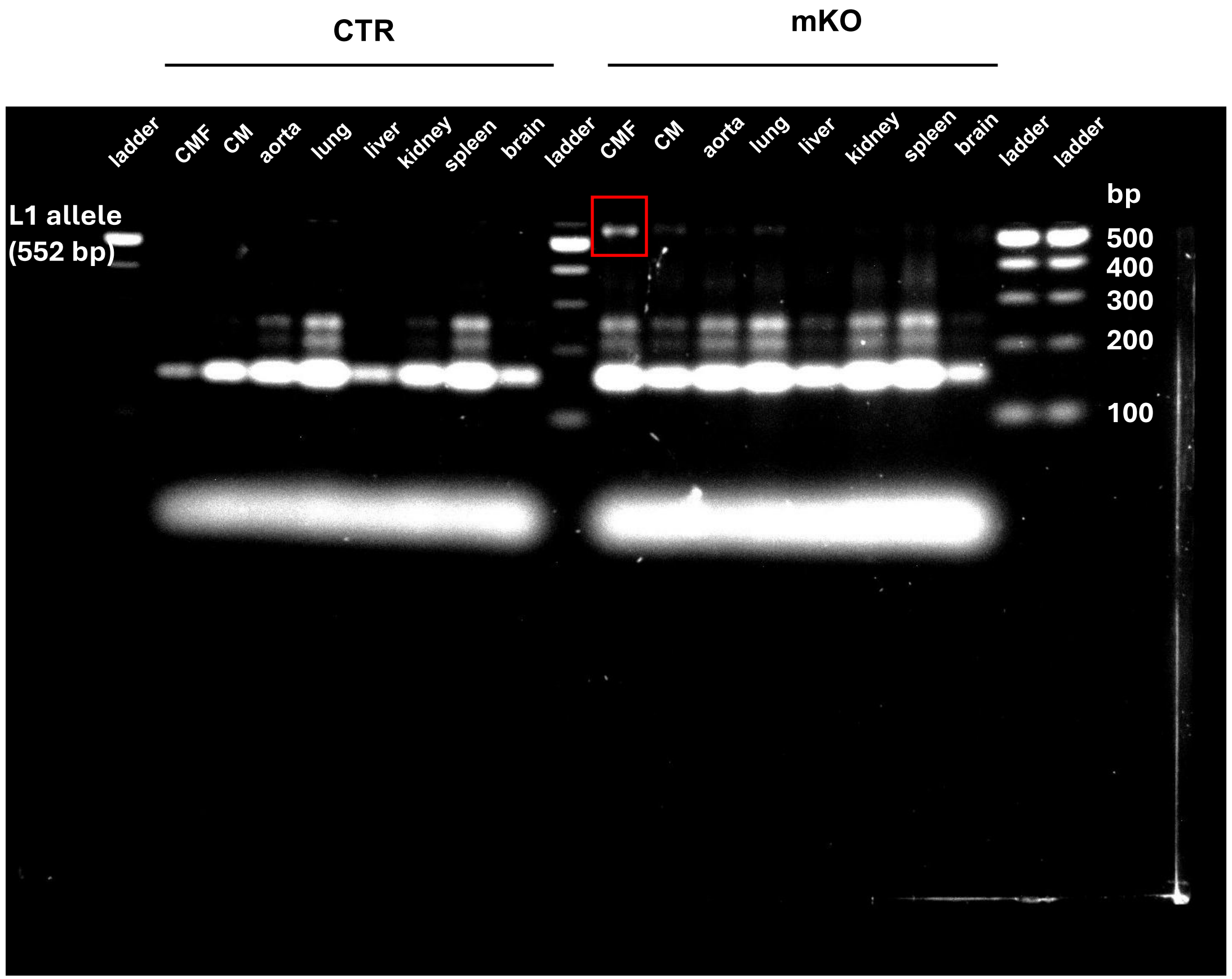

Supplement: Supplementary file 2 [file jciinsight-11-195805-s138.pdf]
